# Exposure to nanoceria impacts larval survival, life history traits and fecundity of *Aedes aegypti*

**DOI:** 10.1371/journal.pntd.0008654

**Published:** 2020-09-25

**Authors:** Mona Doshi, Alexander Bosak, Craig J. Neal, Nour Isis, Udit Kumar, Aadithya Jeyaranjan, Tamil Selvan Sakthivel, Sushant Singh, Alicia Willenberg, Robert B. Hines, Sudipta Seal, Bradley J. Willenberg

**Affiliations:** 1 University of Central Florida College of Medicine, Department of Internal Medicine, Orlando, Florida, United States of America; 2 University of Central Florida College of Engineering, Department of Materials Science and Engineering and Advanced Materials Processing and Analysis Center, Orlando, Florida, United States of America; 3 University of Central Florida College of Medicine, Department of Population Health Sciences, Orlando, Florida, United States of America; 4 University of Central Florida College of Medicine, NanoScience Technology Center, Orlando, Florida, United States of America; International Atomic Energy Agency, AUSTRIA

## Abstract

Effectively controlling vector mosquito populations while avoiding the development of resistance remains a prevalent and increasing obstacle to integrated vector management. Although, metallic nanoparticles have previously shown promise in controlling larval populations via mechanisms which are less likely to spur resistance, the impacts of such particles on life history traits and fecundity of mosquitoes are understudied. Herein, we investigate the chemically well-defined cerium oxide nanoparticles (CNPs) and silver-doped nanoceria (AgCNPs) for larvicidal potential and effects on life history traits and fecundity of *Aedes (Ae*.*) aegypti* mosquitoes. When 3^rd^ instar larvae were exposed to nanoceria in absence of larval food, the mortality count disclosed significant activity of AgCNPs over CNPs (57.8±3.68% and 17.2±2.81% lethality, respectively) and a comparable activity to Ag^+^ controls (62.8±3.60% lethality). The surviving larvae showed altered life history traits (e.g., reduced egg hatch proportion and varied sex ratios), indicating activities of these nanoceria beyond just that of a larvicide. In a separate set of experiments, impacts on oocyte growth and egg generation resulting from nanoceria-laced blood meals were studied using confocal fluorescence microscopy revealing oocytes growth-arrest at 16-24h after feeding with AgCNP-blood meals in some mosquitoes, thereby significantly reducing average egg clutch. AgCNPs caused ~60% mortality in 3^rd^ instar larvae when larval food was absent, while CNPs yielded only ~20% mortality which contrasts with a previous report on green-synthesized nanoceria and highlights the level of detail required to accurately report and interpret such studies. Additionally, AgCNPs are estimated to contain much less silver (0.22 parts per billion, ppb) than the amount of Ag^+^ needed to achieve comparable larvicidal activity (2.7 parts per million, ppm), potentially making these nanoceria ecofriendly. Finally, this work is the first study to demonstrate the until-now-unappreciated impacts of nanoceria on life history traits and interference with mosquito egg development.

## Author summary

Viral diseases like Zika and dengue fevers that are vectored by mosquitoes cause illness and death in populations worldwide. Chemical pesticides are the frontline tools used to control mosquitoes, however, development of resistance by the vectors to these agents overtime ultimately reduces effectiveness. Hence, the development of new pesticides that do not spur the development of resistance is of paramount importance. We therefore developed and characterized various types of well-defined cerium oxide nanoparticles (nanoceria) and studied the impacts on *Aedes (Ae*.*) aegypti* mosquitoes resulting from exposure to these novel agents. We found that nanoceria not only have larvicidal potential, but also alter the life history traits (e.g., reduced egg hatch proportion and altered sex ratio) of the subsequent adult mosquitoes exposed to these nanoparticles during the larval stage. One of the most interesting and important findings of our study is that exposure of female *Ae*. *aegypti* mosquitoes to nanoceria via blood meals significantly reduced egg clutches apparently via arresting oocyte growth, thereby interfering with the ultimate development and production of eggs. Thus, this study reveals until-now-unappreciated impacts of nanoceria exposure on *Ae*. *aegypti* mosquitoes and helps inform how these kinds of agents can be potentially deployed in control strategies.

## Introduction

Rapid and effective management of vector mosquito species is critical to public health due to the tremendous burden the diseases these insects transmit impose on populations worldwide. In order to control mosquito populations and thus limit the spread of diseases such as Zika, dengue and chikungunya, control specialists employ a variety of techniques which act to either kill, repel or suppress mosquitoes. With the ever-increasing prevalence of pesticide resistance to compounds such as pyrethroids, novel techniques of control such as the employment of Wolbachia endosymbionts [1–3] or Sterile Insect Technique (SIT) [4–6] have become essential. Unfortunately, the effective utilization of these methods often necessitates mass-rearing and the subsequent release of thousands to millions of insects [7]. New methods that are less labor-intensive must be developed to bolster the “toolkits” of mosquito control professionals around the world.

Larvicide and adulticide agents have historically been comprised of hormonal analogues [8–10] or potent sodium channel blockers that disrupt development or inhibit neuronal signaling, respectively [11,12]. While effective, resistance to these control agents is both prevalent and increasing due to their widespread use [8,13–15]. Metallic nanoparticles and metal oxides have long been studied in the medical and biotechnology fields due to their ease of synthesis and redox activities [16,17]. Their use within the field of mosquito control though has been limited with most focus being on silver nanoparticles (AgNPs) with effective concentrations being reported in the low ppm range [18]. Previously used AgNPs purportedly work via detoxifying enzyme oxidation/inactivation, enzyme activity reduction, general cellular protein denaturation and gene expression dysregulation [16,19,20]. Cerium oxide nanoparticles (nanoceria; CNPs) have been extensively researched in varied biomedical applications, from cell culture to *in vivo* studies in mammalian models [21–27]. The general bioactivity observed for CNPs is often ascribed to their chemical interaction with the local, biological environment and their catalytic response to (radical) oxygen species [28–30]. In particular, catalysis occurs at surface defects, such as oxygen vacancies, and is highly dependent on physicochemical conditions [31–35]. Incorporation of the known larvicidal agent, silver, into the CNP structure may allow for an increased total larvicidal response. Specifically, it may be that ceria and silver could function as co-catalysts which impact mosquito health conditions via unique reaction pathways affecting endogenous biochemical substrates. Similar effects have been observed for industrial applications of metal catalysts on cerium oxide supports [36]. Drawing from previous studies in other biological systems [37–40] and one study performed with *Ae*. *aegypti* larvae and pupae [41], CNPs suggest a substantial potential for use as vector mosquito larvicides and adulticides.

Presently, we have synthesized, characterized and assessed CNPs and AgCNPs with the goal of determining insecticidal activities. Additionally, we discovered exposure to these agents affected *Ae*. *aegypti* fecundity, life history traits and egg clutch. We have thoroughly characterized CNPs and AgCNPs utilizing standard material characterization techniques to best understand how these particles affect mosquitoes. From our studies, we have shown similar kill rates in AgCNP-exposed conditions to those with higher titers of free Ag^+^. Additionally, through fluorescence microscopy, we were able to confirm the presence of nanoparticles within mosquitoes (larvae). Mosquitoes that were not killed by the nanoparticles exhibited altered life history traits including reductions in egg clutch size, decreases in egg hatch proportions and altered sex ratios depending on whether they were treated as larvae or adults.

This study serves as an important step in expanding the use of inorganic nanoparticles as control agents. The amount of silver utilized to induce larval death is reduced in AgCNP versus free silver ion (0.22ppb vs. 4.2ppm, respectively). Activating silver for larval killing, through incorporation with cerium oxide nanomaterial, will greatly reduce the silver amount(s) available for release into the environment, thus improving environmental conscientiousness while retaining efficacy as tools for mosquito control personnel. Further, we have shown that mosquitoes (both larvae and adults) that do not die following exposure see reductions in key life history traits such as total eggs oviposited or hatch proportions. This method of metal-modified nanomaterial treatment may provide a new, non-lethal control measure that ideally would abrogate concerns surrounding spurred resistance.

## Methods

### Synthesis of non-doped cerium oxide nanoparticles (CNP)

Particles were synthesized via a well-established method [42]. 1.48g of cerium nitrate hexa-hydrate (99.9%; Sigma Aldrich) were dissolved in 50mL of dH_2_O and stirred for 30min to ensure complete pre-cursor dissolution. Then, 5mL of 28–30% ammonium hydroxide (NH_4_OH, ACS reagent; Sigma Aldrich) was added dropwise to the solution under vigorous stirring and left stirring for 4h. The solutions were then washed three times by centrifugal separation and solvent replacement with dH_2_O. The resulting solution was ultra-sonicated for 30min and left to stand overnight at room temperature allowing larger, less colloidally stable particles to sediment. The following day, sedimented particles were discarded and the stable, dispersed phase particles were used without further modification.

### Synthesis of silver-doped ceria nanoparticles (AgCNP)

Particles were synthesized via a variation and modification of a published protocol [43]. 160mM cerium nitrate hexa-hydrate and 40mM silver nitrate (99.9%; Sigma Aldrich) solutions were prepared separately prior to combining in equal volumes to yield final concentrations of 80mM and 20mM, respectively. 10mL of 400mM sodium hydroxide (NaOH, ACS reagent; Sigma) solution (oxidizer) were added dropwise under vigorous stirring (450rpm) for 20min. Reagent counter-ions were removed from solution and the solution pH was reduced to <4 via centrifugation and washing with dH_2_O (solution was deep black). From here, the solution was ultra-sonicated for 20min and left to stand at room temperature overnight. Suspended particles were decanted from this solution carefully leaving behind sedimented material.

### Chemical treatment of CNP and AgCNP formulations

To remove any free/un-reacted silver ions and secondary nanoscale silver phases from the AgCNP formulation, the decanted phase was diluted to 2/3 initial strength with 28–30% NH_4_OH and stirred vigorously overnight. Solution coloration changed from black to a pale-yellow color over the 24h stir time, indicating dissolution [44]. The particle suspensions were then centrifuged at 10,000rpm for 8min and washed with dH_2_O with this step repeated three times. Washing removed the degraded silver components and ammonia (NH_3_) as well as returned the sample pH to acidic conditions (~3.5 pH). CNP particles were treated in a similar manner and attained comparable pH. Particles were left to stand for one day prior to use in biological experiments.

To promote particle colloidal stability prior to biological experimentation, the particle suspensions were ultra-sonicated with micro-probe ultra-sonication. Micro-probe ultra-sonication (3min with 10s on and 20s off pulse increments; average power density: 13W; Misonix Ultra-sonicator) was determined to impart total particle stability at 1mg/mL for >6h, as determined via UV-Vis measurements (measured at 292nm over time).

### X-Ray photoelectron spectroscopy (XPS)

Samples were deposited dropwise onto cleaned, cut silicon wafers ([100] grain orientation) in 20μL increments and allowed to air dry. Measurements were collected using a Thermo ESCALAB-250Xi spectrometer at room temperature in ultra-high vacuum chamber, <7×10^−9^ mbar, with a monochromatic Al-Kα radiation source, operating at 300W (15kV, 20mA) with a 250μm spot size. A flood gun was used for all measurements to provide charge compensation to the samples during measurement (to prevent sample charging, damage during measurement and modulating peak characters). 3–5 scans were acquired and averaged for each element (carbon, oxygen, silver and cerium). Measurement analysis and instrument operation were performed using Avantage software from Thermo. The C 1s peak at 284.6eV was used as a reference for binding energy calibration.

### Inductively coupled plasma-mass spectrometry (ICP-MS)

For evaluating the level of free silver ion contaminants, 1mL of 1mg/mL nanoceria solution was centrifuged three times at 13,000rpm for 30min. The resultant supernatant was transferred to a new centrifuge tube and was sent to Huffman Hazen Laboratories (a division of Hazen Research, Inc., Golden, CO) for ICP-MS evaluation using their established protocols.

### High-resolution transmission electron microscopy (HR-TEM)

Measurements were performed at the University of Florida Research Service Centers Herbert Wertheim College of Engineering. Samples, diluted with de-ionized (DI) water, were added as a single drop onto lacey carbon support film grids (Ted Pella #01894). Sample drying was performed by holding grids over a 100°C hotplate until dry. Samples were allowed to cool to room temperature prior to plasma cleaning (30s in Ar/O_2_ plasma environment) to remove potential carbonaceous environmental contaminants. HR-TEM was performed using an FEI Tecnai F20 TEM.

### Raman spectroscopy

Samples were ultra-sonicated for 15min and deposited dropwise onto clean borosilicate microscope slides in 200μL increments. Measurements were performed using a Renishaw RM 1000B Micro-Raman spectmeter with an Argon laser at 514nm excitation. Acquisition intensity was optimized by referencing and indexing wavenumber against a silicon standard.

### X-ray diffraction (XRD)

Samples in petri dishes were dried overnight under vacuum in an oven at ~60°C. Powders were obtained by scraping dried particles with a laboratory spatula and subsequent fine grinding with mortar and pestle. Powders were added to steel substrates and flattened prior to measurement. Measurements were performed using a PANalytical Empyrean powder X-ray diffractometer. A 1.8kW copper X-ray tube was used, with wavelength of 1.54Å, as a source with theta goniometer for powder incident diffraction.

### Mosquito rearing

*Aedes aegypti* eggs originally obtained from the United States Department of Agriculture-Agricultural Research Service, Center for Medical, Agricultural and Veterinary Entomology (USDA-ARS-CMAVE, Gainesville, FL) were continuously generated as previously described [45]. Briefly, eggs were collected and wrapped in Kimwipe tissue paper (Kimberly-Clark; Irving, TX) and kept in the dark within air-tight containers with a small cup of DI water. For each batch, 800 eggs (by weight; ~8mg) were brushed off the cards and transferred to a glass vial containing 7.5mL larval food in DI water (60:40; liver powder (MP Biologics, Santa Ana, CA) and brewer’s yeast (Insectsales.com)). The eggs were shaken vigorously and dispersed into 3L of DI water. Larval trays were incubated at 29–30°C and noted as day zero (d0) and larval food added at d3 (7.5mL), d4 and d5 (10mL). At d6 or when over 50% of mosquitoes had pupated, the larvae/pupae were poured and rinsed over a 500μm strainer and transferred to an 8oz. (50cm^2^ surface area) cup with 200mL DI water. The majority of the mosquitoes emerged within 24h and this counted as day post-emergence (DPE) one. 10% sucrose was provided *ad libitum* via a saturated cotton ball placed atop rearing cages (8×8” Bioquip, Rancho Dominguez, CA).

### UV-Vis spectroscopy

For the determination of absorbance spectra, a DU 800 spectrophotometer (Beckman Coulter, Brea, CA) was used. Wavelength scans from 200-800nm were acquired at 1200nm per minute using a 1-cm path length quartz cuvette. Spectra were analyzed in OriginPro 9.0 software (OriginLab Corporation, Northampton, MA).

### Fluorescence spectroscopy

A PTI QuantaMaster 400 spectrofluorometer equipped with a near-infrared InGaAs detector (Horiba Scientific; Kyoto, Japan) was used for fluorescence detection. Fluorescence spectra were acquired in a 1-cm path length quartz cuvette using an excitation wavelength of 405nm with a halogen bulb and slits set at 3nm. Emission was collected from 425nm-700nm at an acquisition time of 0.1s/nm with slits set at 3nm. Spectra were analyzed in OriginPro 9.0 software (OriginLab Corporation, Northampton, MA).

### Confocal fluorescence imaging of larvae

To confirm nanoceria uptake by larvae, 3^rd^ instar *Ae*. *aegypti* larvae were incubated with nanoceria for 4h followed by overnight fixation with 10% neutral buffered formalin (NBF). Then the larvae were transferred to fresh phosphate buffered saline (PBS). For imaging, larvae were put delicately in a drop of PBS on a slide and imaged with a Zeiss 710 laser scanning confocal microscope at 10× magnification using Zen 2010 (Zeiss; Jena, Germany) software and 405nm laser as the excitation wavelength.

### Larvicidal properties assessment of nanoceria

Refer to S1 Flowchart and S1 Dataset for better understanding of the flow of experimental design and figures. To assess the larvicidal properties of nanoparticles, 3^rd^ instar *Ae*. *aegypti* larvae were separated into groups of 10 larvae per cup with 40mL of DI water. In order to further test whether the presence of the larval food had any effect on the nanoparticles, 3^rd^ instar larvae were treated with nanoparticles both with and without the presence of larval food in the rearing water. Conditions are outlined in Table 1. For conditions with larval food, 100μL of larval food was added after the administration of nanoparticles or vehicle control (DI water). For conditions without larval food, food was added either 24h or 48h after administration of nanoparticles. Control groups with no nanoparticles served as negative controls for conditions with and without larval food. Positive controls for larvicidal activity consisted of treating larvae with 4.2mg/L silver nitrate (AgNO_3_) in the presence of larval food. All conditions contained six (6) or nine (9) individual replicates that were pooled into three emergence cups of 20 or 30 larvae/pupae respectively on d6 of experimentation as more than 50% of surviving larvae had pupated by this time point. Mosquitoes were allowed to emerge as adults, were fed 10% sucrose *ad libitum* and the numbers of dead larvae/pupae, remaining larvae/pupae and emerged adults were recorded on d13.

**Table 1:** Experimental conditions for larvicidal, adulticidal and fecundity studies using nanoceria.

Condition concetration/ Experiment	Control	CNP	AgCNP	AgNO_3_	CNP+AgNO_3_	AgNP
**Larval Treatment (with food and food withheld for 24 or 48h)**	0mM	0.010mM	0.010mM	4.2mg/L	N/A	N/A
**Adult Treatment through Sucrose Feeding Solution**	0mM	0.1mM**/** 0.5mM**/** 1mM	0.1mM/ 0.5mM/ 1mM	4.2mg/L**/** 8.5mg/L	N/A	N/A
**Adult Treatment through blood** **(bulk experiment)**	0mM	1mM	1mM	4.2mg/L	1mM CNP + 4.2mg/L (AgNO_3_)	N/A
**Adult Treatment through blood (Individual experiment)**	0mM	1mM	1mM	4.2mg/L	N/A	1mM

### Fecundity study after nanoceria treatment on larvae

After recording the number of adults that emerged from control or nanoparticle-treated conditions, mosquitoes were provided with 10% sucrose (average 5 DPE), after which they were starved for 18h. Mosquitoes were provided a heated blood meal (mechanically defibrinated bovine blood) for 1h and 24h post blood meal (PBM), oviposition cards were introduced into the individual cups. Five (5) days PBM, oviposition cards were collected, wrapped in Kimwipe tissue and stored in a box within an incubator for two (2) days. The eggs were then transferred into airtight containers with a small cup of DI water for humidity and kept in the dark. Approximately two (2) weeks later, egg weight was recorded by brushing off the eggs in a tared weighing plate and recorded on a Mettler College150 digital scale (Mettler Toledo; Columbus, OH). Images of eggs were acquired on a stereoscopic microscope equipped with an AmScope MU1803 digital camera (Irvine, CA) at identical magnification.

To assess the effects larval exposure to nanoparticles had on subsequent progeny, 100 (or all when less than 100) eggs from each group were counted and placed in 600μL of larval food within a glass vial. After shaking the vial vigorously, the eggs were transferred to and incubated in 200mL DI water in a specimen cup for hatching. Upon following the rearing protocol described above, the numbers of dead and remaining larvae/pupae, adults emerged and their sex was recorded. To determine if nanoparticle exposure during the larval stage affected the subsequent progeny size, random females from each condition were imaged and had their bodily measurements recorded. For measuring body length, the region from mid thorax to the end of the last segment was taken into consideration, while width of the body was measured at the fourth segment. For wing measurement, the whole length of the wing was taken into consideration. Wing width was measured at the center of the wing.

### Adulticidal properties assessment of nanoparticles

Three (3) DPE *Ae*. *aegypti* females were cold anaesthetized and sorted into groups of 50 for testing of adulticidal properties of nanoparticles. Mosquitoes were starved overnight on 5 DPE and fed nanoparticles within a previously developed sugar-based mosquito feeding solution (10% sucrose, 15mM NaCl, 1mM Na_2_CO_3_, 1μM MgCl_2_ and 5μM ATP) [45]. Experimental conditions are outlined in Table 1. 400μL of nanoparticle solution or control were transferred into glass membrane feeders (Chemglass; Vineland, NJ) with parafilm stretched across the bottom and 37°C water was circulated through the jackets of the glass feeders to maintain temperature. Mosquitoes were fed for 1h, transferred back into the rearing incubator and fed 10% sucrose *ad libitum* for 5d, at which time the number of live/dead mosquitoes was assessed.

### Administration of nanoceria in adult mosquitoes through blood meal (bulk experiment)

To test whether nanoparticles affected egg production in adults who had yet to be exposed, 5 DPE adult *Ae*. *aegypti* females were fed 37°C mechanically-defibrinated bovine blood containing nanoparticles (**Table 1**). After 1h, mosquitoes were visually confirmed to have engorged a blood meal prior to being transferred to an incubator and placed within cups with oviposition cards. Five (5) days PBM, the oviposition cards were collected, stored and assessed as described above. The fecundity of the resultant progeny was also assessed as described above.

### Administration of nanoceria in adult mosquitoes through blood meal (individual experiment)

Five (5) DPE female *Ae*. *aegypti* adult mosquitoes were sorted into specimen cups in groups of 50. After being starved overnight, mosquitoes were provided a meal of 37°C defibrinated bovine blood with or without nanoparticles. Nanoparticles or AgNO_3_ were added to blood immediately prior to feeding and sonicated for 20min to ensure even suspension (**Table 1**). After 1h of feeding, mosquitoes were cold anaesthetized and placed individually into oviposition cups. Only mosquitoes which had fed, as confirmed by visual inspection, were used for experimentation. Mosquitoes were allowed five (5) days PBM to oviposit eggs.

For the generation of the fluorescent microscopy atlas of egg growth timeline, only control blood fed (i.e., no nanoparticles) mosquitoes were utilized. Blood fed mosquitoes were placed into cups in groups of three (3), provided sugar *ad libitum* and dissected at 0h, 2h, 4h, 6h, 8h, 12h, 16h, 20h, 24h, 28h, 36h and 48h time points. Ovaries from each time point were fixed and stained as described below.

### Dissection, fixation and staining of ovaries

At the conclusion of each respective experimental time point, mosquitoes were cold anaesthetized and immediately dissected. After removing the legs and wings to minimize debris, a 10μL drop of phosphate buffered saline (PBS) was placed at the rear of the mosquito. Next, under a microscope, the second to last abdominal segment was gripped with a pair of fine forceps and, while holding the bottom of the thorax with another pair of forceps, teased away from the body and into the PBS. The ovaries were carefully dissected away, placed within a fresh 10μL PBS drop to wash any debris and then transferred to a 60μL drop of 10% neutral buffered formalin (NBF) placed atop a glass coverslip. Tissues were fixed for 30min at room temperature before being rinsed 3× with PBS. Following fixation, ovaries were permeabilized with 0.5% Triton-X 100 for 30min at room temperature. Next, tissues were rinsed 3× with PBS before being stained with NucBlue Live Ready Probe and ActinGreen 488 ReadyProbes (Invitrogen; Carlsbad, CA) for 2h protected from light. Ovaries were once again rinsed 3× with PBS before being placed in a 12μL drop of ProLong Glass (Invitrogen; Carlsbad, CA) placed atop a glass slide and covered with a 12mm glass coverslip (12-545-80 12CIR.-1, Fisherbrand; Hampton, NH). All samples were imaged on a Zeiss 710 laser scanning confocal microscope at 10× and 63× magnification using Zen 2010 software (Zeiss; Jena, Germany). The excitation wavelengths used for NucBlue and ActinGreen 488 were 405nm and 488nm, respectively. Z-stacks of both channels were overlaid and maximum intensity z-projections were created using ImageJ version 1.52b (National Institutes of Health; Bethesda, MD).

### Statistical analysis

The experimental repetitions are detailed in the figure captions for each corresponding graph. A number of outcomes consisting of proportions (displayed as percentages) and discrete/continuous outcomes were evaluated. For the analysis of proportions, population proportion estimates with standard errors for each group were obtained. The chi-square test was used to evaluate if at least two proportions differed between the groups. If the chi-square test for an overall difference was statistically significant, all pairwise differences were then evaluated. Tukey-style post-hoc multiple comparisons adjustment was used to adjust p-values in the manner described by Zar [46].

For the analysis of discrete/continuous outcomes, evaluation of whether the data met normality assumptions was performed. For comparisons when all groups had ≤6 cups, normality was not assessed and the Kruskal-Wallis test was used to evaluate if at least two medians were statistically different. If the Kruskal-Wallis test was significant, all pairwise differences were then tested. Post-hoc multiple comparisons adjustment was used to adjust p-values using Dunn’s test as described by Zar [47]. For discrete or continuous outcomes which met sample size requirements, normality was assessed a number of ways including: comparison of means/medians, Q-Q (quantile-quantile) plots, the Shapiro-Wilk normality test, and tests of skewness and kurtosis [48]. If the data were deemed to have a non-normal distribution, the Kruskal-Wallis test with post-hoc multiple comparisons adjustment as described above was used to assess group differences. If data met the normality assumption, population mean estimates with standard errors were obtained for each group. Analysis of variance (ANOVA) was used to assess whether at least two population mean estimates were different. If the ANOVA F-statistic was significant, post-hoc Tukey-Kramer multiple comparisons adjustment was used to evaluate all pairwise comparisons. Finally, for the comparison of eggs produced per female according to group (Fig 11), both nonparametric and parametric tests were used. Several females in some groups produced no eggs which rendered the data non-normal. Thus, nonparametric hypothesis testing was used. The data was then restricted to females that produced eggs (i.e., zeros were removed), which rendered the data normal. Thus, ANOVA followed by t-tests comparing each group with the control group was performed. No multiple comparisons adjustment was implemented because the data was altered (by removing the zeros), and these were *a priori* hypothesis tests. Statistical significance was defined as α = 0.05. Analysis was performed with SAS version 9.4 (SAS Institute Inc., Cary, NC) with figures produced by Microsoft Excel 2016 (Redmond, WA). (Analysis files for all the figure: S1 File–S7 File)

## Results

### X-ray photoelectron spectroscopy (XPS) and inductively coupled plasma—mass spectrometry (ICP-MS)

**Fig 1:**
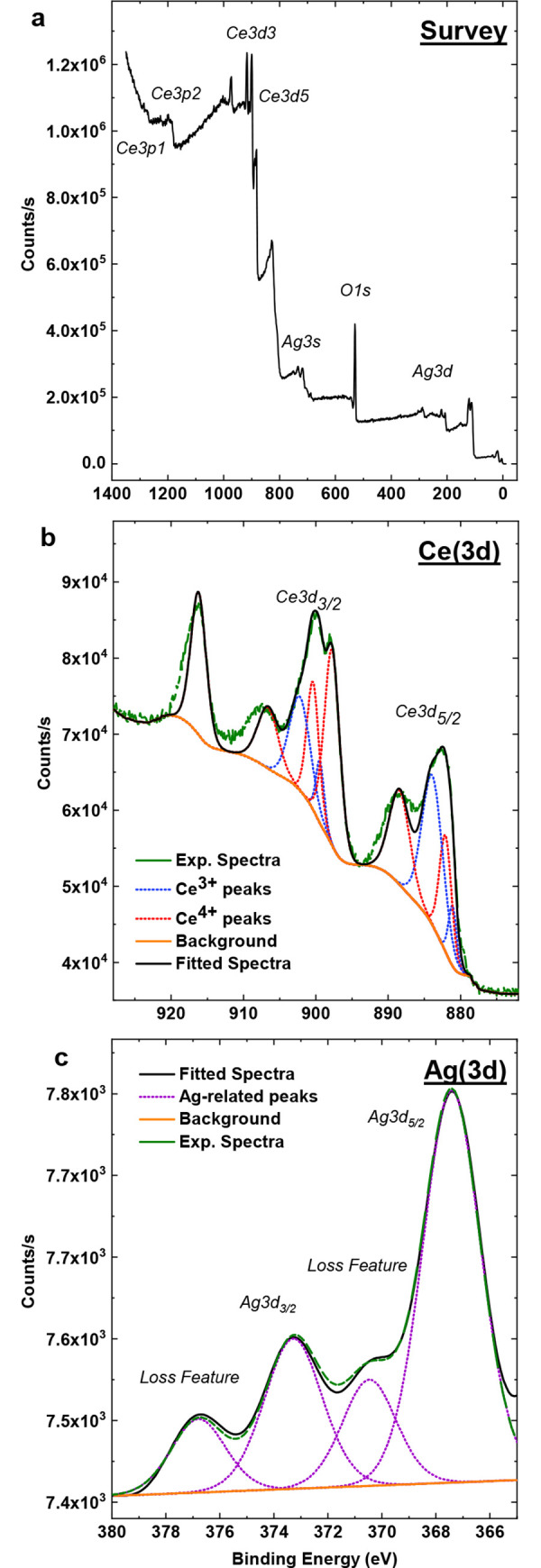
High resolution X-ray Photoelectron Spectroscopy (XPS) of AgCNP. (**a**) Survey, (**b**) fitted cerium (Ce3d), and (**c**) silver (Ag3d) spectra demonstrate silver-fraction and cerium chemical state. Cerium spectrum (**b**) peaks at 879.7, 888.1, 902.0, and 916.5eV are ascribed to Ce^3+^ states while 897.7, 882.0, 884.1, 899.7, 903.5, and 906.5eV are attributed to Ce^4+^ character. Peak area integration for both redox states suggests 37.4% Ce^3+^ fraction. Silver fraction with AgCNPs was on average ~0.72at% according to peak fitting over cerium, silver, and oxygen related peaks in survey spectrum. (**c**) Ag3d spin-orbit states at 367.5eV and 373.4eV peak broadening and shifts connote presence of metallic and non-zero oxidation state. Loss of peaks at 370.3eV and 376.8eV are related to zero-valence silver states.

XPS is commonly used to evaluate the surface chemistry of solid material samples. Signals from this spectroscopy arise from electron emissions and are particular to material elemental compositions. Emission events occur due to electron transitions from specific core states and, thereby, provide chemical information such as redox state (via binding energy and peak(s) character) and coordination environment (e.g., oxygen coordination with metals or as hydroxide; via binding energy shifting). The x-rays used have a penetration depth of ~5 nm, which is of the order of the nanoparticle size, allowing approximate determination of particle chemical composition (i.e., atomic %—at%—of silver, cerium and oxygen) and distribution of redox states (ratio between Ce^3+^ and Ce^4+^) (**Fig 1**) [49]. XPS analysis showed 0.72at% silver incorporation for AgCNPs (**Table 2**). Critical to our studies is the measurement of silver species released due to desorption from or dissolution of AgCNPs. ICP-MS, using inductively coupled plasma to atomize the sample and create atomic ions, can detect metals and many non-metals in liquid samples at very low concentrations. Therefore, solid phase nanoparticles were pelleted via centrifugation and the aqueous supernatant was subjected to ICP-MS analysis, as shown in Table 2. This data suggests the presence of negligible amounts of silver contaminants/dissociation products (i.e., free silver ion or silver nanoclusters) in AgCNP solution. The ratio of Ce^3+^ to Ce^4+^ redox states within the material was calculated as 34.7% Ce^3+^ for AgCNPs. This calculation was produced by dividing the sum of Ce^3+^-specific peak integrals by the total integrated signal for cerium within the spectra (details of this calculation are provided in S1 Calculations). Peak breadth and position for Ag3d signals suggest presence of both zero and non-zero valence state silver; with this distinction possibly related to the position of silver species relative to the cerium oxide lattice.

**Table 2:** ICP-MS and XPS analysis of chemically well-defined nanoparticles post-treatment to remove free silver. Data shows that negligible free silver remains in the solutions, indicating observed effects on mosquitoes are due to incorporated silver and not free silver ion or metal. The at% of silver doped inside nanoceria was markedly lower than the level predicated (only 0.72at% silver was incorporated in AgCNP when the predicted amount was 20%).

	ICP-MS (Ag^+^ content in supernatant, post-synthesis treatment (ppm))	XPS (at% of Ag doped post-synthesis treatment)
**AgCNP**	0.003	0.72% (0.22ppb)

### High resolution–transmission electron microscopy (HR-TEM)

**Fig 2:**
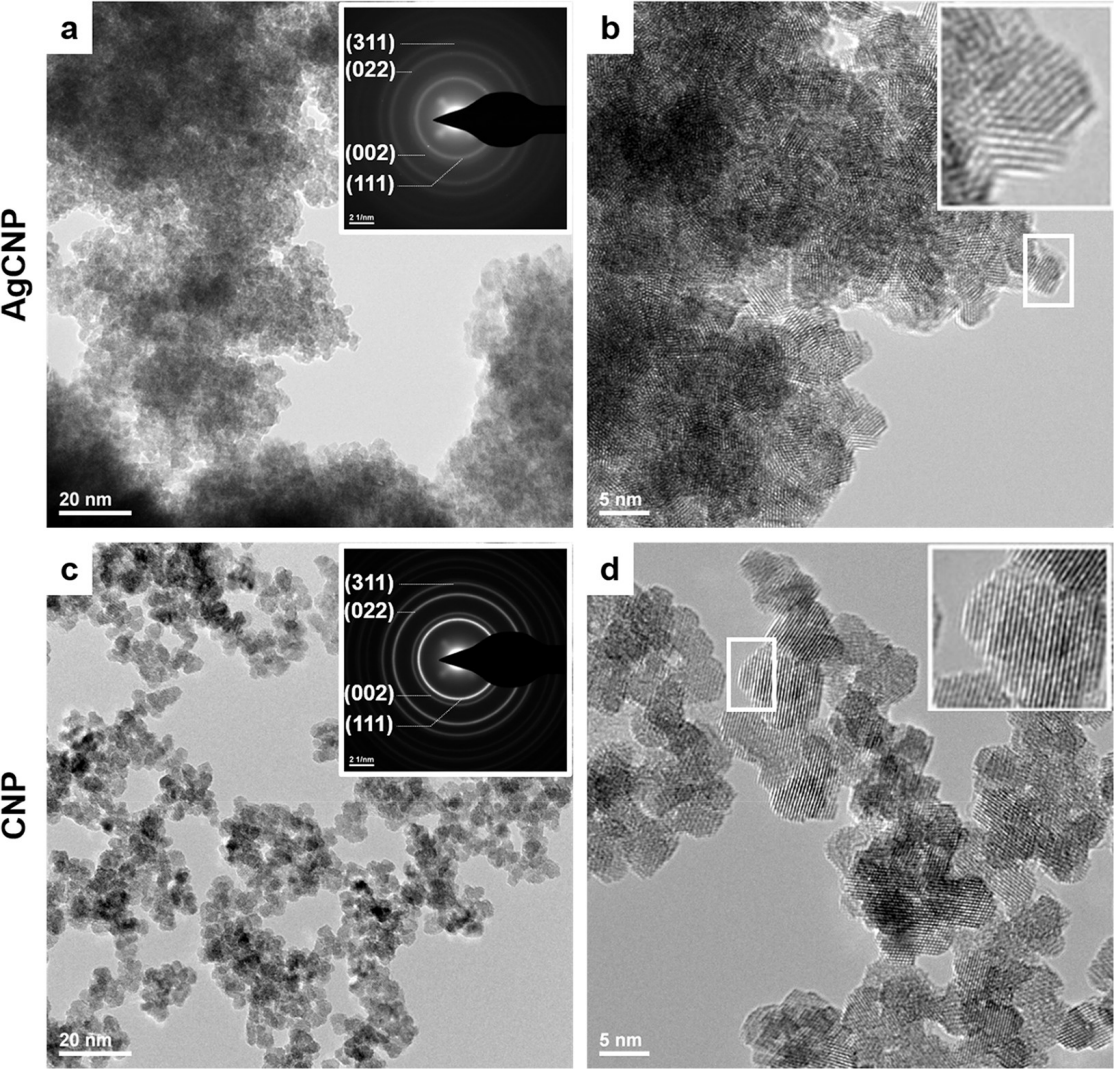
Representative HR-TEM micrographs of nanoceria. (**a,b**) AgCNP and (**c,d**) CNP at 145,000× and 400,000×, respectively. Selected area electron diffraction inset (**a,c**) show ring structure characteristic of nanostructured materials. **b,d** insets evidence lattice fringes of highly crystalline particles.

Transmission electron microscopy allows visualization of solid materials with a resolution of ~0.1nm. Using an electron diffraction probe and resultant diffraction patterns, HR-TEM micrographs evidence particle crystallinity, morphology, and non-solvated dimensions. HR-TEM images are presented for both CNPs and AgCNPs showing 5-7nm particles (**Fig 2**). CNPs show clearly defined regular surfaces and limited aggregation. AgCNPs appear largely similar to CNPs character (e.g., morphology and particle size). However, the particles may be more prone to aggregation, though this is complicated by the high particle concentration in the sample images. Further, the surfaces of these particles appear slightly less regular as compared to CNPs. It may be that surface etching occurred due to the high concentration of NH_4_OH to solubilize contaminant silver nanoparticles and the presence of surface adsorbed or doped silver leeching from the particles during the treatment. All particle images show a high degree of crystallinity as evidenced by clear and regular lattice fringes as well as characteristic ring patterns in selected area electron diffraction (SAED).

### Raman spectroscopy

**Fig 3:**
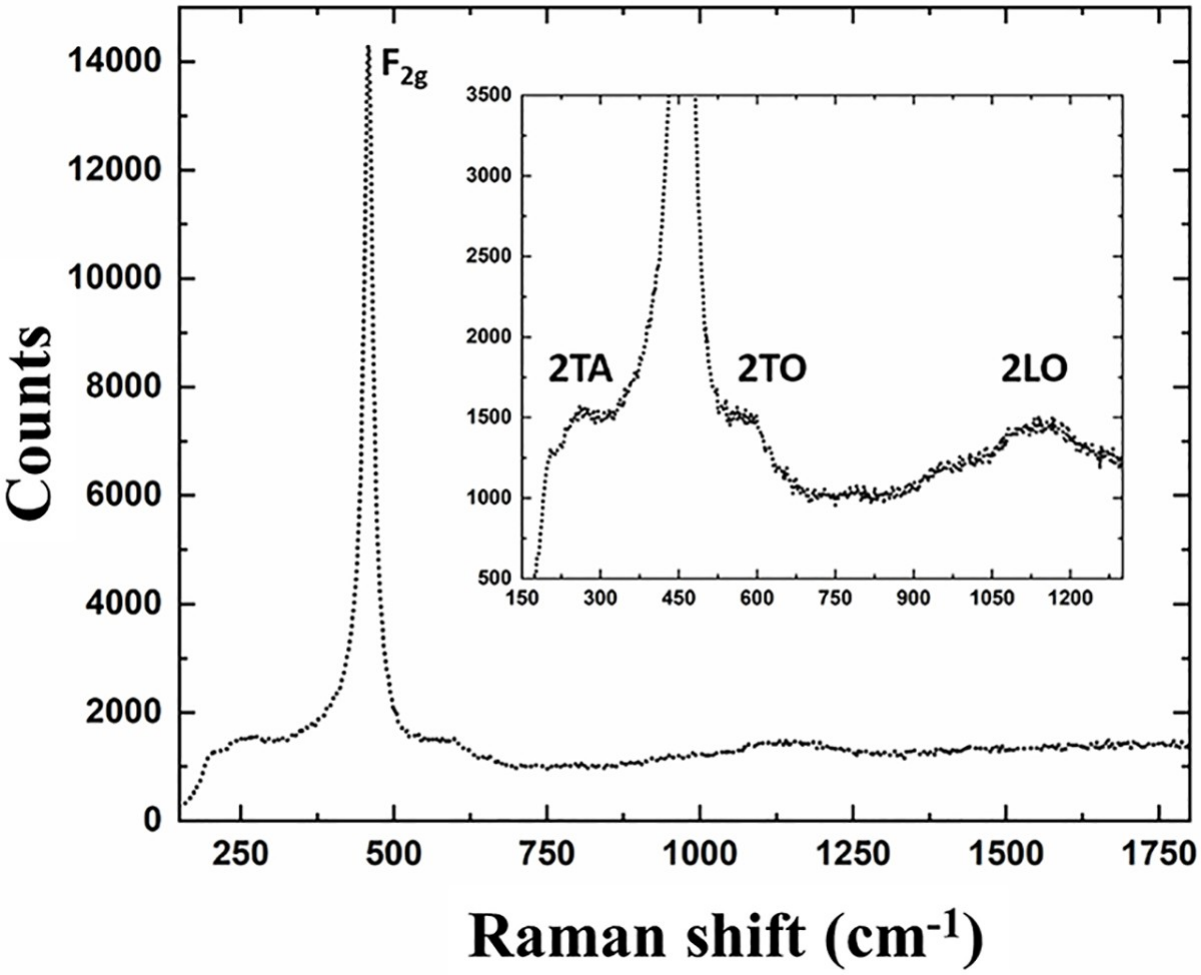
Raman spectroscopy for AgCNP. Prominent *F*_2g_ peak (465cm^-1^) characteristic of Ce-O-Ce breathing mode. Line broadening (FWHM: 20.74cm^-1^) is substantial and indicates low crystallite diameter (nanocrystalline features). 2TA (265 cm^-1^), 2TO (597 cm^-1^), and 2LO (1142 cm^-1^) second order Raman peaks emerge following chemical treatment and suggest surface/interfacial modification.

Raman spectroscopy uses light signals produced via inelastic Raman scattering as structural information. Herein, Raman signals were used to characterize changes to phonon modes particular to the cerium oxide crystal/defect structures. The obtained spectrum for AgCNP **(Fig 3**) shows characteristic Raman shift peaks for CeO_2_ [50]. Specifically, the spectrum is dominated by the first order Ce-O-Ce symmetric vibration (breathing) mode at ~465cm^-1^ [51]. Additional second order phonon peaks are also observable with largely *F*_2g_ and *E*_g_ symmetry character. These second order phonons are well-pronounced, relative to ceria nanoparticles produced by a comparable method (generally, only the 465cm^-1^ is well-resolved). Additionally, the Raman line width for AgCNP is broad (full width half maximum: ~20.74cm^-1^ at the F_2g_ peak) with slight asymmetry. Significant line broadening is common for nano-scale phases (inhomogeneous strain broadening due to phonon confinement [50]) and suggests small crystallite diameter as well as a concomitant increase in grain boundary volume for the sample.

### X-Ray diffraction (XRD)

**Fig 4:**
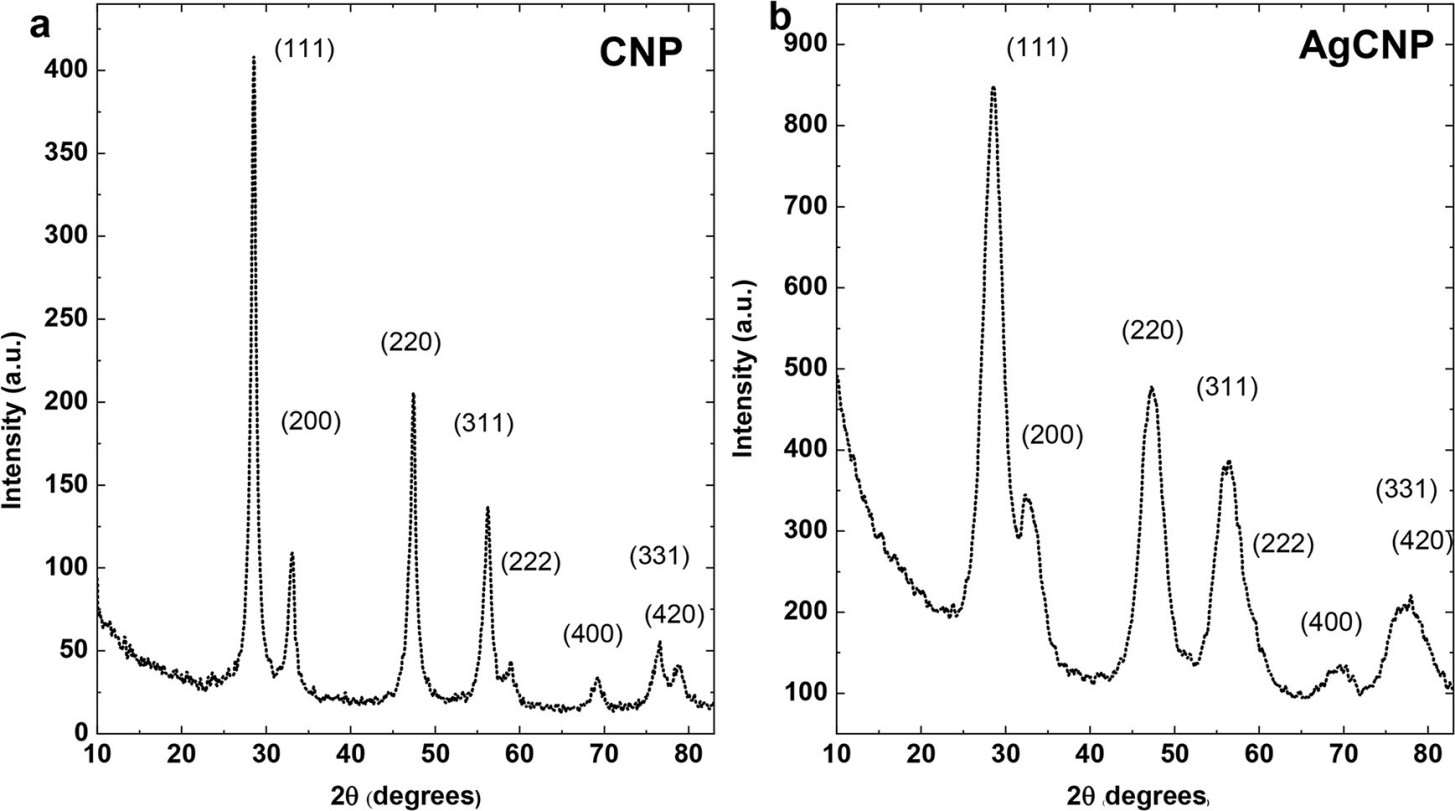
X-ray Diffraction (XRD) measurement via CuKα irradiation. (**a**) CNP and (**b**) AgCNP; peak indices are common to cerium oxide fluorite structure ($\mathrm{Fm}\bar{3}m$ space group). Peaks in (**b**) show substantially increased broadening relative to those in (**a**) (0.668 to 3.342 FWHM at (111)) highlighting a potential decrease in crystallite diameter due to susceptibility to chemical treatment. All peaks match with ceria (CeO_2_) reference pattern, ICSD:186923, ICDD:98-018-6923.

XRD uses X-rays to probe the crystal structure of solid samples by passing the X-rays through the lattice structure and experience diffraction upon interaction with atoms at lattice points, leading to characteristic signal patterns. Herein, XRD is used to confirm presence of cerium oxide phases and absence of silver (oxide) phases. XRD for AgCNP was performed and compared with CNP (**Fig 4**). Peak indices confirmed fluorite ceria structure (space group $\mathrm{Fm}\bar{3}m$) and were comparable between CNP and AgCNP [51]. In particular, no additional peaks were observed (e.g., related to silver/silver oxide phases) following chemical treatment. However, full width half maximum measurements show substantial broadening of AgCNP (calculated using (111) peaks; 0.668 to 3.342 Δ2θ⁰, ~4× scaling of peak breadth).

### Larvicidal impact of nanoceria on 3^rd^ instar *Aedes aegypti* larvae

**Fig 5:**
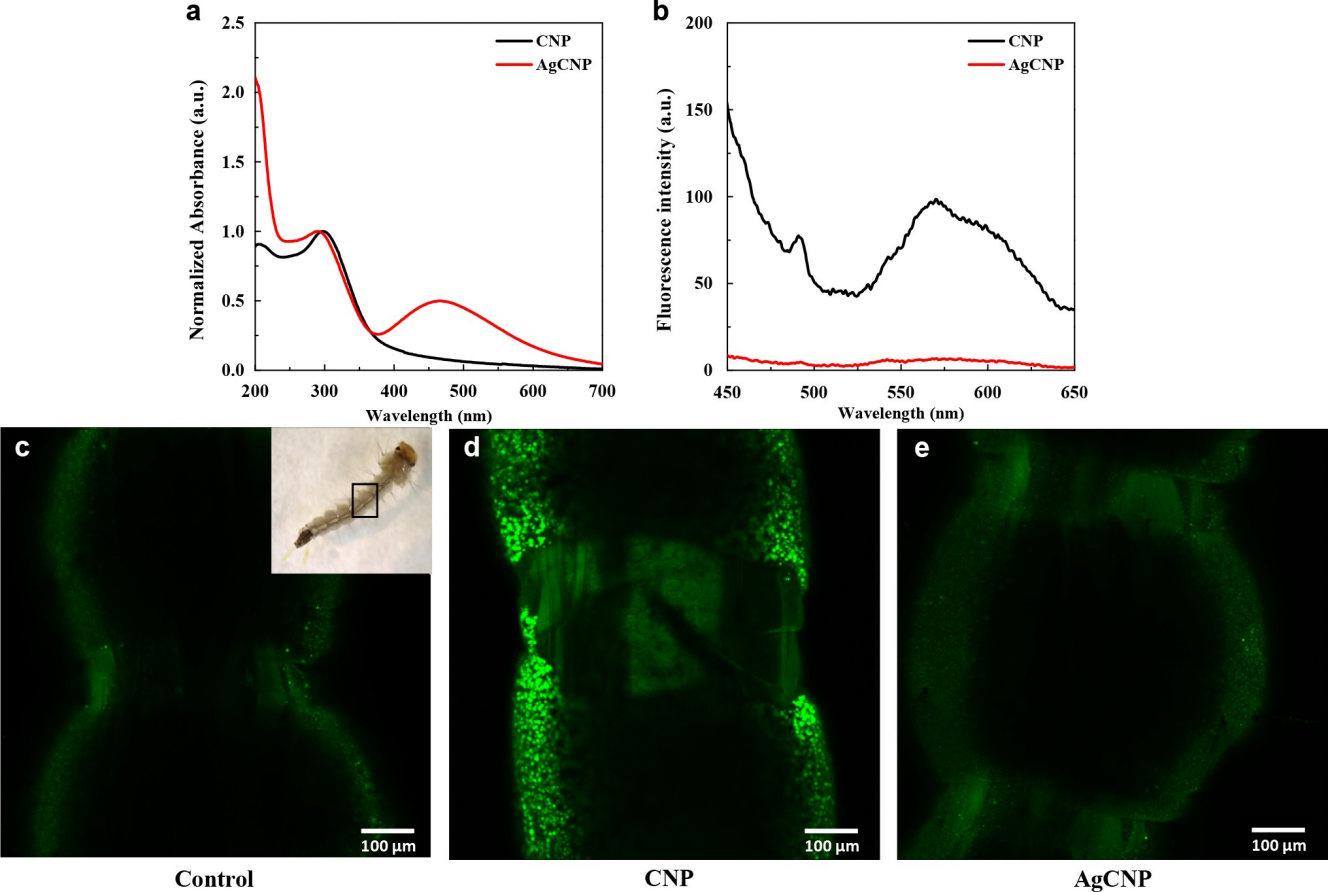
Optical properties of nanoceria. (**a**) Absorbance spectra and (**b**) emission spectra of CNP (black line) and AgCNP (red line) depicting λ_max-absorption_ of CNP at 300nm and λ_max-amission_ at 570nm. Doping with Ag quenches the emission of CNP, though AgCNP show absorbance at 466nm in addition to the 300nm peak. (**c-e**) Confocal fluorescence microscopy of larvae exposed to nanoceria. (**c**) Gut of control larva (No nanoceria), (**d**) gut of larva treated with CNPs and (**e**) gut of larva treated with AgCNPs. Bright fluorescence is seen in the larva treated with CNPs; objective lens: 10×, excitation laser: 405nm.

CNPs and AgCNPs were studied for their pesticidal properties against *Ae*. *aegypti* larvae. Optical properties of nanoceria were exploited to confirm their consumption by 3^rd^ instar larvae. The absorption spectrum of CNPs shows λ_max-absorption_ at 300nm, while the emission spectrum shows λ_max-emission_ at 570 nm (**Fig 5A and 5B**). Doping with Ag quenched emission of nanoceria, though there is absorption in the visible spectrum with λ_max-absorption_ at 466nm, in addition to the 300nm peak. Confocal fluorescence images (**Fig 5C–5E**) show bright fluorescence emanating from the gut of a larva exposed to CNPs. As AgCNP has no fluorescence, larvae exposed to AgCNPs do not show fluorescence in the gut.

**Fig 6:**
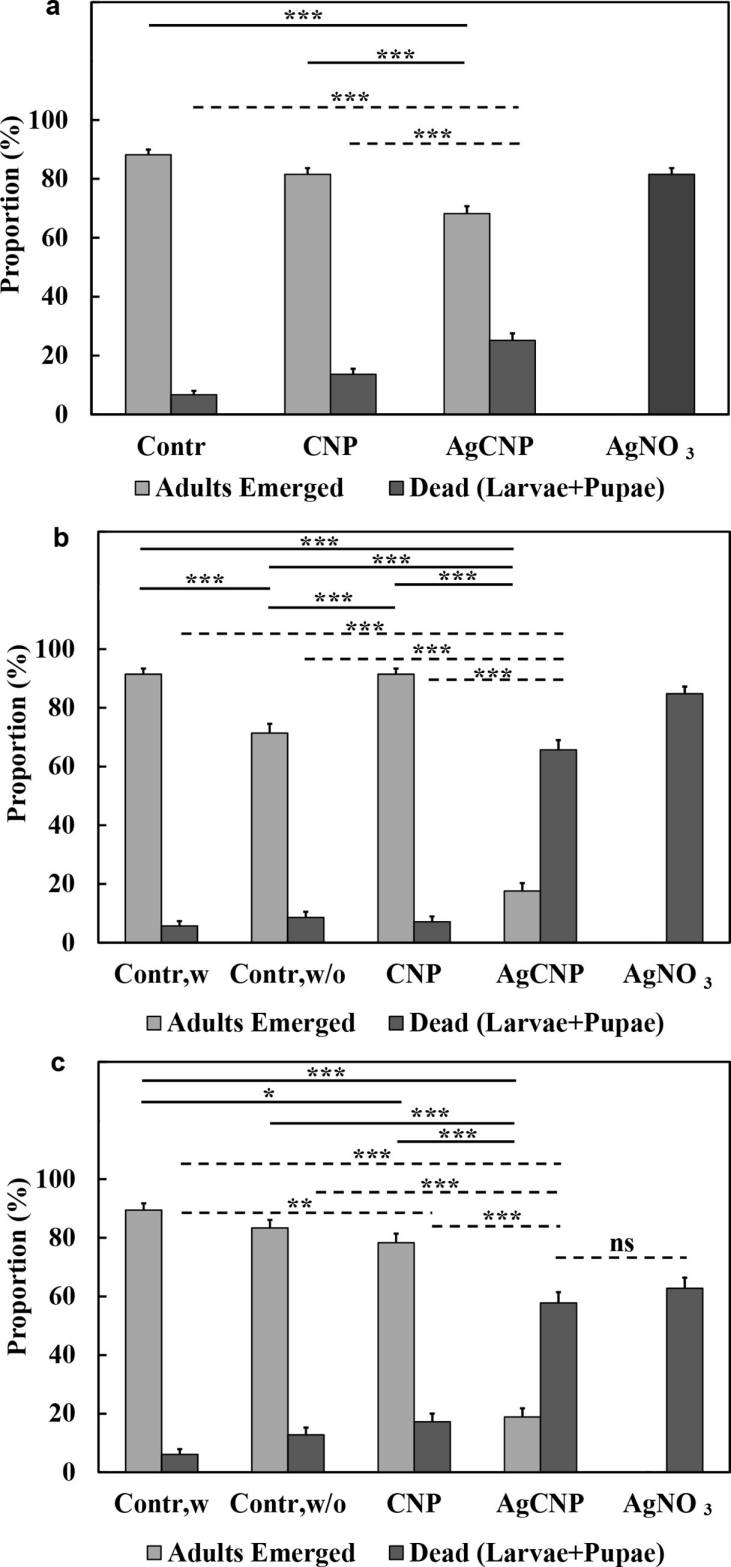
Larvicidal activity of nanoparticles. (**a**) When larval food was present in the rearing water, AgCNPs showed significant larvicidal effect when compared to control (p-value <0.001). When larval food was withheld for either (**b**) 24h or (**c**) 48h following treatment with AgCNPs, the observed larvicidal activity was even greater compared to control (p-value <0.001). The highest larvicidal activity was observed in the positive control, 4.2mg/L AgNO_3_ (2.7mg/L Ag^+^). No adults emerged in this positive control condition in any of the experiments. Further, the mean proportion of dead larvae and pupae for the AgNO_3_ condition was significantly different from all other conditions in all experiments (p-value <0.001, comparison lines not shown) except for the AgCNP condition in (**c**), which was not significantly different (ns). Contr,w: control, larval food present; Contr,w/o: control, larval food withheld. *: p-value <0.05, **: p-value <0.01, ***: p-value <0.001. Solid lines indicate significantly different comparisons between the mean proportions of adults emerged in different treatment groups. Dashed lines indicate significantly different comparisons between the mean proportions of dead larvae + pupae in different treatment groups, as well as one not significantly different comparison marked as ns. Error bars = SEP. All experiments were repeated at least three (3) times with three (3) cups of 20–30 pooled larvae each, yielding at least nine (9) cups in total for each experimental condition.

When larval food was present in rearing water during the nanoparticle exposure (**Fig 6A**), AgCNP and AgNO_3_ had significantly greater larvicidal impact on 3^rd^ instar *Ae*. *aegypti* larvae in comparison to the control group (25.2±2.39% and 81.5±2.14% larvae dead, respectively, p-values <0.001). All other pairwise comparisons between experimental groups were also significantly different (p-values <0.001). Believing the presence of abundant organic matter (i.e., larval food) may blunt the nanoceria larvicidal activity, experiments were performed where food was withheld for 24h and 48h side-by-side with the appropriate controls for each condition. Now in the AgCNP groups compared to their respective 24h and 48h food-withheld controls, the mean proportions of larvae killed were 65.7±3.28% and 57.8±3.68% (p-values <0.001) (**Fig 6B and 6C**). Controls for each food-withheld condition as well as all CNP conditions, had only 5–20% larvae/pupae death with 70–90% adult emergence. As a positive larvicidal control, 4.2mg/L AgNO_3_ (2.7mg/L Ag^+^) was used and resulted in 60–80% larval death with 0% adult emergence in all conditions.

### Impacts on fecundity and life history traits following nanoceria exposure of larvae

**Fig 7:**
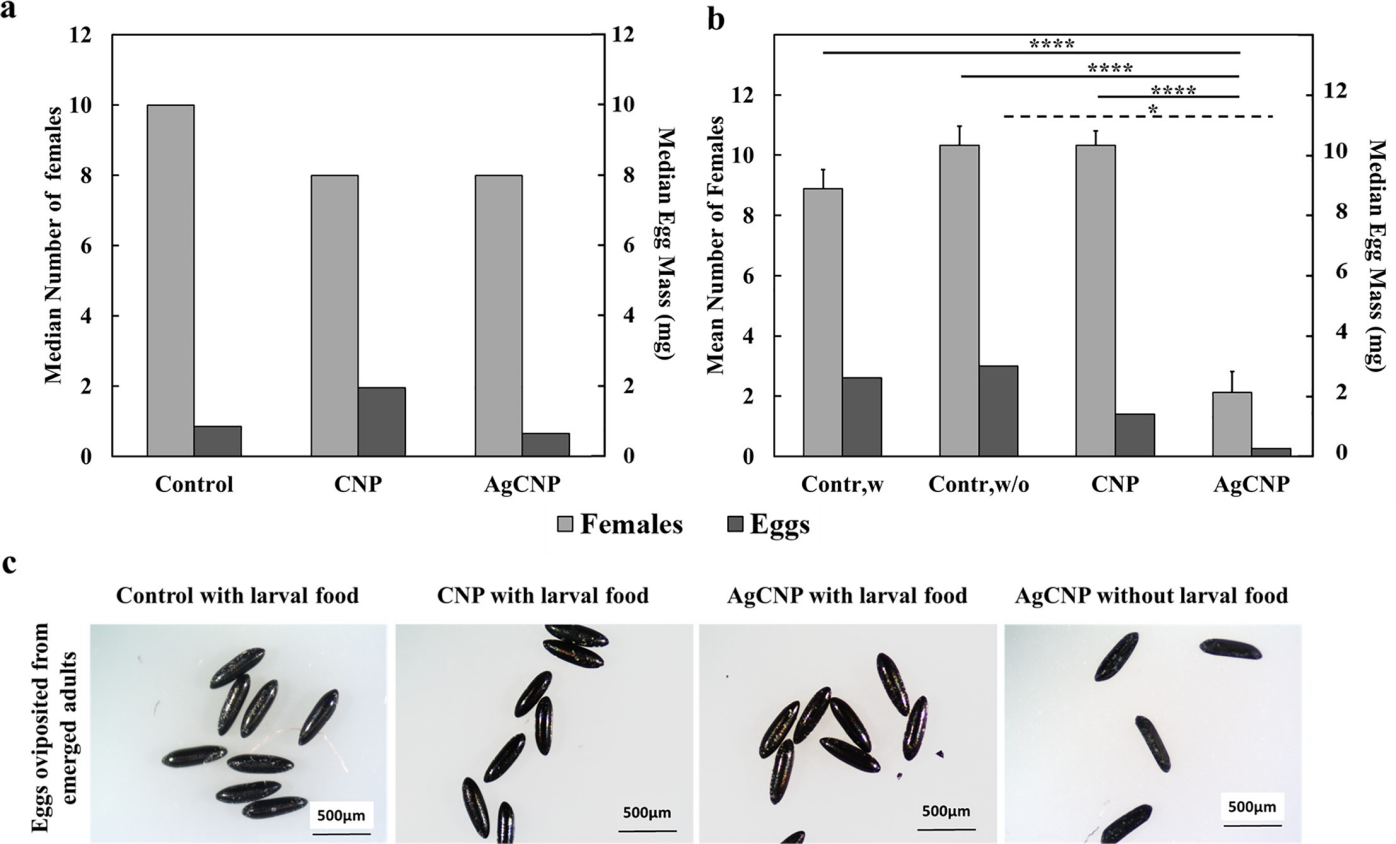
Effects on fecundity of nanoparticle-treated *Ae*. *aegypti* larvae. Third (3^rd^) instar larvae that were treated with nanoparticles in Fig 6 experiments were then allowed to emerge as adults and were given a blood meal five days post emergence; the resultant eggs were collected, weighed and characterized. (**a**) Median numbers of females emerged from nanoparticle-treated larvae with food in the rearing water (**Fig 6A**) and median egg masses produced from these females were not significantly different from control or each other. (**b**) When larval food was withheld for 48h following nanoparticle exposure (**Fig 6C**), there was a significant decrease in the mean number of females that emerged in the AgCNP group compared to both control and CNP-treated conditions (p-value <0.0001). Correspondingly, the observed median egg mass in the AgCNP-treated condition was significantly lower compared to the food-withheld control group (p-value <0.05). (**c**) Representative images of eggs from each group showed that eggs from the AgCNP-treated condition where larval food was withheld appeared collapsed in comparison (scale bar = 500μm). Contr,w: control, larval food present; Contr,w/o: control, larval food withheld. *: p-value <0.05, ****: p-value <0.0001. Solid lines indicate significantly different comparisons between the mean number of females in different treatment groups. Dashed lines indicate significantly different comparisons between the median egg masses in different treatment groups. Error bars = SEM. Experiments for (**a**) were repeated twice with three (3) cups of females each, yielding six (6) cups in total for each experimental condition. Experiments for (**b**) were repeated three (3) times with three (3) cups of females each, yielding nine (9) cups in total for each experimental condition.

**Fig 8:**
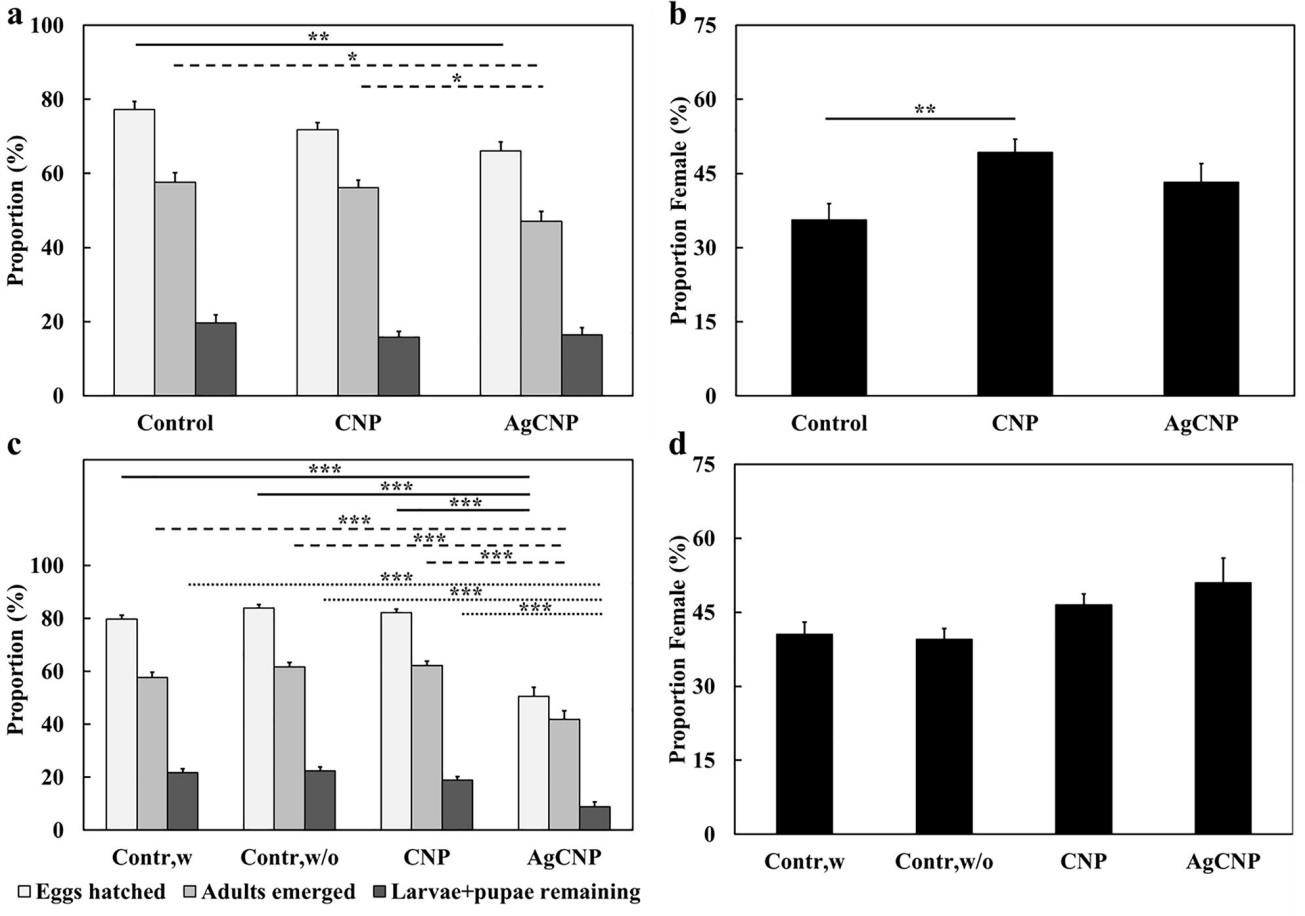
Effects on hatch proportions and the proportion of females (sex ratio) in the progeny of nanoparticle-treated *Ae*. *aegypti* larvae. Eggs produced from mosquitoes treated with nanoceria during the 3^rd^ larval instar stage from Fig 7 experiments were harvested and used to produce the next generation of mosquitoes. (**a**) Mean proportions of eggs hatched, adults emerged and larvae + pupae remaining when larval food was present during nanoparticle exposure (**Fig 7A**). Compared to control, the mean proportion of eggs hatched and adults emerged in the AgCNP-treated group was significantly lower (p-values <0.01 and 0.05, respectively). Mean proportion of adults emerged in the AgCNP group was also significantly lower than that of the CNP treatment group (p-value <0.05). (**b**) Mean proportion of females (sex ratio) observed in the adults emerged in (**a**); this proportion was significantly higher in CNP-treated group compared to control (p-value <0.01). (**c**) Mean proportions of eggs hatched, adults emerged and larvae + pupae remaining when larval food was withheld for 48h during nanoparticle exposure (**Fig 7B**). In the AgCNP treatment group, all studied mean proportions were significantly lower than the corresponding mean proportions in all other groups (p-value <0.001). (**d**) Mean proportion of females (sex ratio) observed in the adults emerged in (**c**); the mean sex ratios of all groups were not significantly different from each other. Contr,w: control, larval food present; Contr,w/o: control, larval food withheld. *: p-value <0.05, **: p-value <0.01, ***: p-value <0.001. Solid lines in (**a**) and (**c**) indicate significantly different comparisons between the mean proportions of eggs hatched in different treatment groups, while in (**b**), the solid line indicates a significantly different mean proportion of females between groups. Dashed lines indicate significantly different comparisons between the mean proportions of adults emerged in different treatment groups. Dotted lines indicate significantly different comparisons between the mean proportions of larvae + pupae remaining in different treatment groups. Error bars = SEP. Experiments for (**a**) and (**b**) were repeated twice with three (3) cups of eggs each, yielding six (6) cups in total for each experimental condition. Experiments for (**c**) and (**d**) were repeated three (3) times with three (3) cups of eggs each, yielding nine (9) cups in total for each experimental condition.

**Fig 9:**
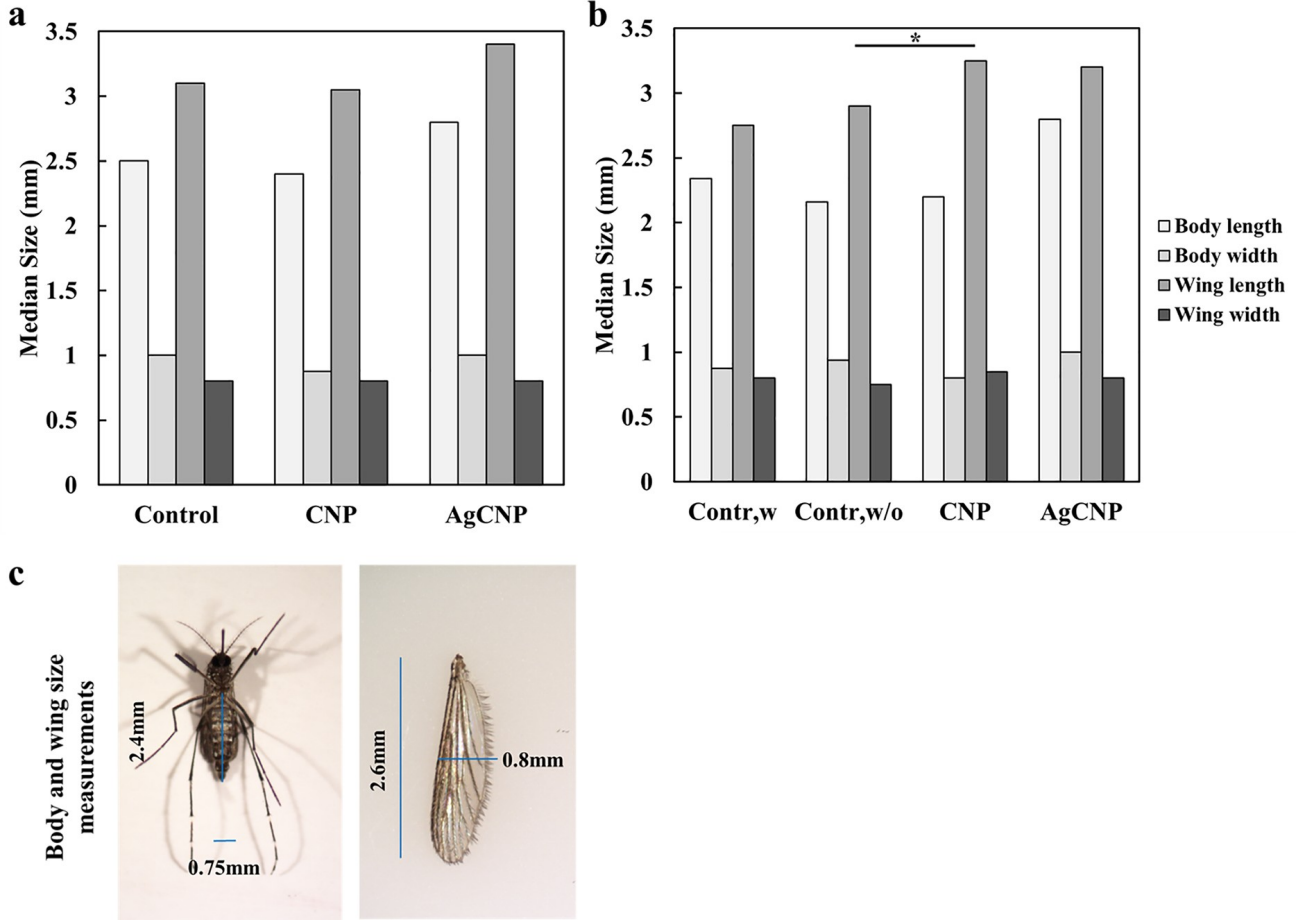
Effects on progeny life history traits of nanoparticle-treated *Ae*. *aegypti* larvae. Wing and body measurements of female progeny from eggs of mosquitoes treated with nanoceria in the 3^rd^ instar larval stage (**Fig 8**) were analyzed to assess nanoceria effects on life history traits. (**a**) Median body and wing measurements of female progeny when larval food was present during nanoparticle exposure (**Fig 8B**); all measurements in all groups were not significantly different. (**b**) Median body and wing measurements of female progeny when larval food was withheld for 48h during nanoparticle exposure (**Fig 8D**). All measurements in all groups of this experiment were not significantly different except for the median wing length of the CNP-treated group, which was larger than the corresponding control food-withheld group (p-value <0.05). (**c**) Representative images of a mosquito and wing overlaid with blue lines indicating corresponding body and wing measurements. Contr,w: control, larval food present; Contr,w/o: control, larval food withheld. *: p-value <0.05. Solid line in (**b**) indicates a significantly different comparison between the medians of wing length between groups. Experiments for (**a**) were repeated twice with three (3) cups of mosquitoes each, yielding six (6) cups in total for each experimental condition. Experiments for (**b**) were repeated three (3) times with two (2) cups of mosquitoes each, yielding six (6) cups in total for each experimental condition.

Adults that emerged from all conditions were provided a blood meal and allowed to oviposit eggs. No significant effects on oviposited egg masses were observed in conditions where larval food was present (**Fig 7A**, p-value = 0.16). However, when larval food was withheld, the average masses of eggs produced from females emerged from AgCNP-exposed larvae were significantly lower than controls (**Fig 7B**, p-value <0.0001). Images of eggs from AgCNP-exposed larvae in food-withheld conditions showed many eggs with a collapsed morphology (**Fig 7C**). Speaking further to the viability of these eggs, in the case of food-present experiments, significant difference was observed in the mean proportions of eggs hatched between AgCNPs and control groups (**Fig 8A**, p-value <0.01). However, in food-withheld experiments, the mean proportion of eggs hatched in the AgCNP-treated group reduced significantly to 50.6±3.23% compared to the food-withheld control mean proportion of 84±1.30% (**Fig 8C**, p-value <0.001). Nanoceria exposure in the presence of larval food showed that the mean proportion of females in the CNP-treated group was significantly higher than the control (**Fig 8B**, p-value <0.01).When experiments were carried out in food-withheld conditions, the mean proportions of females in the resultant progeny of all groups were similar (**Fig 8D**). The wing and body dimensions of female mosquitoes were measured to assess the effect of larval nanoceria exposure on resultant progeny size (**Fig 9).** When nanoceria treatment was performed in presence of food (**Fig 9A**), the dimensions of the progeny in all groups were similar. However, under food withheld conditions (**Fig 9B**), the median wing length in the CNP-treated group was greater than the corresponding control (p-value <0.05).

### Life history traits of female *Aedes aegypti* mosquitoes after exposure to nanoceria

**Fig 10:**
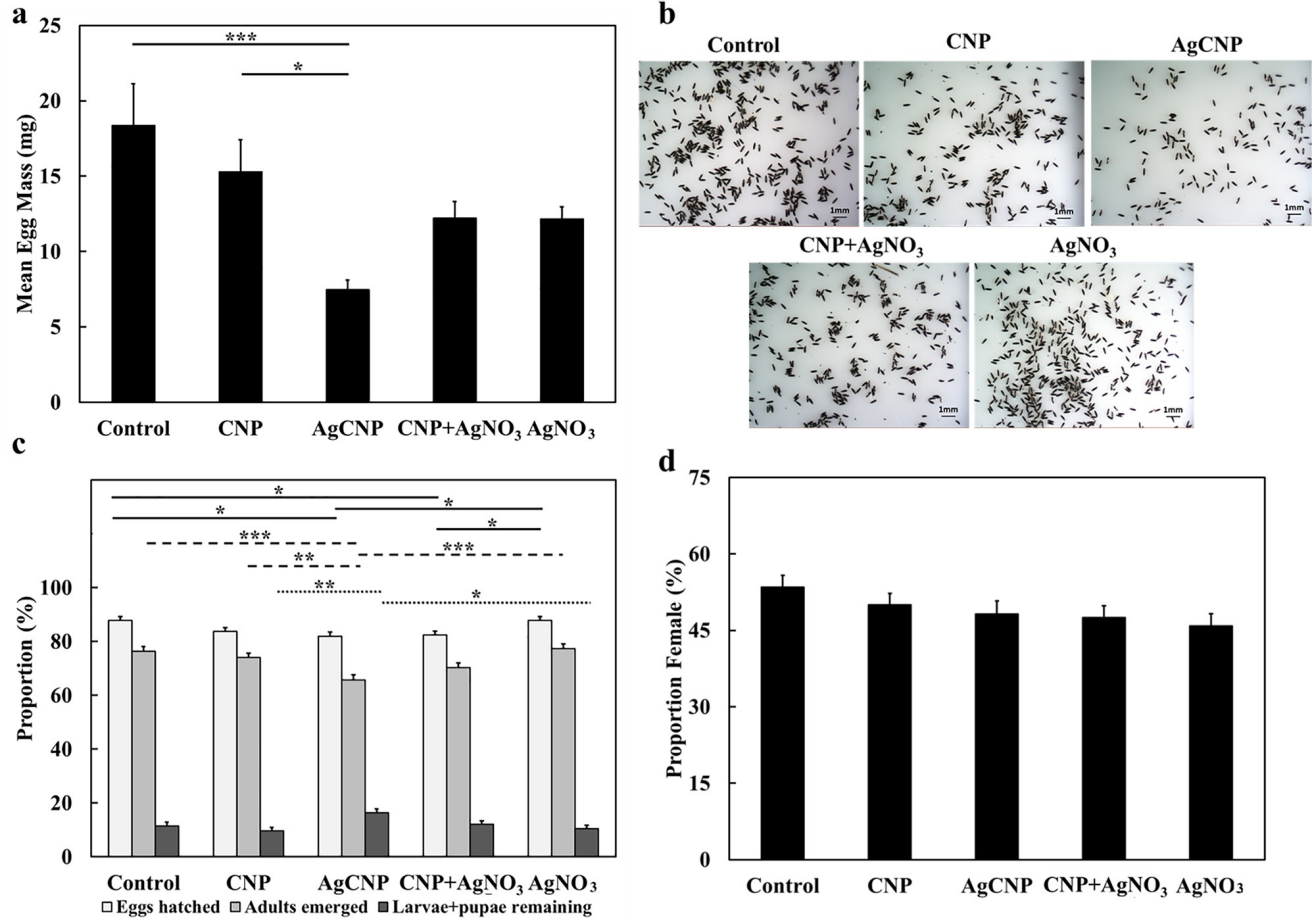
Effects of nanoparticles on fecundity of *Ae*. *eagypti* female mosquitoes when administered via a blood meal (bulk experiment). Nanoparticles were fed to *Ae*. *aegypti* females in bulk via a blood meal and subsequent fecundity studies were performed. (**a**) Mean egg masses produced from female mosquitoes fed nanoceria. Females in the AgCNP-treated group produced a significantly lower mean mass of eggs compared to the control and CNP-treatment groups (p-values <0.001 and 0.05, respectively). (**b)** Representative images of eggs oviposited by control and nanoceria-treated females; morphology of eggs in all the groups appears similar. (**c**) Mean proportions of eggs hatched, adults emerged and larvae + pupae remaining. The mean proportions of eggs hatched in the AgCNP and CNP + AgNO_3_ groups were significantly lower than that of the control and AgNO_3_ groups (p-values <0.05). The mean proportion of adults emerged in the AgCNP group was significantly lower compared to those of the control, AgNO_3_, and CNP treatment groups (p-values <0.001, 0.001 and 0.01, respectively). The mean proportion of larvae + pupae remaining in the AgCNP group was significantly lower than those of CNP and AgNO_3_ groups (p-values <0.01 and 0.05, respectively). (**d**) Mean proportion of females (sex ratio) observed in the adults emerged in (**c**); the mean sex ratios of all groups were not significantly different form each other. *: p-value <0.05, **: p-value <0.01, ***: p-value <0.001. Solid lines in (**a**) indicate significantly different comparisons between the mean egg masses in different treatment groups, while in (**c**), the solid lines indicate significantly different comparisons between mean proportions of eggs hatched in different treatment groups. Dashed lines indicate significantly different comparisons between the mean proportions of adults emerged in different treatment groups. Dotted lines indicate significantly different comparisons between the mean proportions of larvae + pupae remaining in different treatment groups. Error bars in (**a**) = SEM, Error bars in (**c**) and (**d**) = SEP. All Experiments were repeated three (3) times with 1–3 cups of 50 mosquitoes each (**a**) or 100 eggs each (**c**) yielding 6–7 cups in total for each experimental condition.

The dose dependent administration of CNPs, AgCNPs and AgNO_3_ in a sucrose-based feeding solution had no pesticidal effect on adult female *Ae*. *aegypti* mosquitoes with approximately 80% of these mosquitoes surviving 5-days post treatment for all conditions (**S1 Fig**). A significant reduction in egg clutch was observed when AgCNPs were administered to mosquitoes via a blood meal. When 50 mosquitoes fed on warmed blood with and without nanoparticles through a membrane feeder, AgCNP-exposed mosquitoes produced only 7.5±0.63mg of eggs on average compared to a mean egg mass of 18.4±2.76mg for the control group (**Fig 10A**, p-value <0.001). CNP-treated adults showed no significant reduction in clutch size compared to control, but the mean egg mass for this group was significantly larger compared to the AgCNP group (**Fig 10A**, p-value <0.05). The mean egg masses of the CNP + AgNO_3_ and AgNO_3_ treatment groups were not significantly different from any of the other groups. Representative images of eggs collected from this experiment indicate that the egg morphology is normal and similar in all conditions (**Fig 10B**), unlike that of the larval exposure experiment where eggs in the AgCNP group appeared collapsed (**Fig 7C**).

The mean proportion of eggs hatched in the AgCNP-treated group was significantly lower than the control and AgNO_3_ group mean proportions (**Fig 10C**, p-value <0.05). The mean proportion of adults emerged in the AgCNP group was also significantly lower than those of the control and AgNO_3_ groups (p-values <0.001) and that of the CNP group (**Fig 10C**, p-value <0.01). Further, the mean proportion of larvae + pupae remaining in the AgCNP-treated group was significantly lower than those in the CNP and AgNO_3_ groups (**Fig 10C**, p-values <0.01 and 0.05, respectively). All of these significant reductions observed for AgCNP exposure were smaller however than those observed in the larval food-withheld studies (**Fig 8C**). Also dissimilar from larval food-withheld experimental results, there were no significant variations observed in the mean proportions of females in the subsequent progeny from adults treated with nanoceria (**Fig 10D**).

### Effect of nanoceria exposure via blood meal on egg production of individual female *Aedes aegypti*

**Fig 11:**
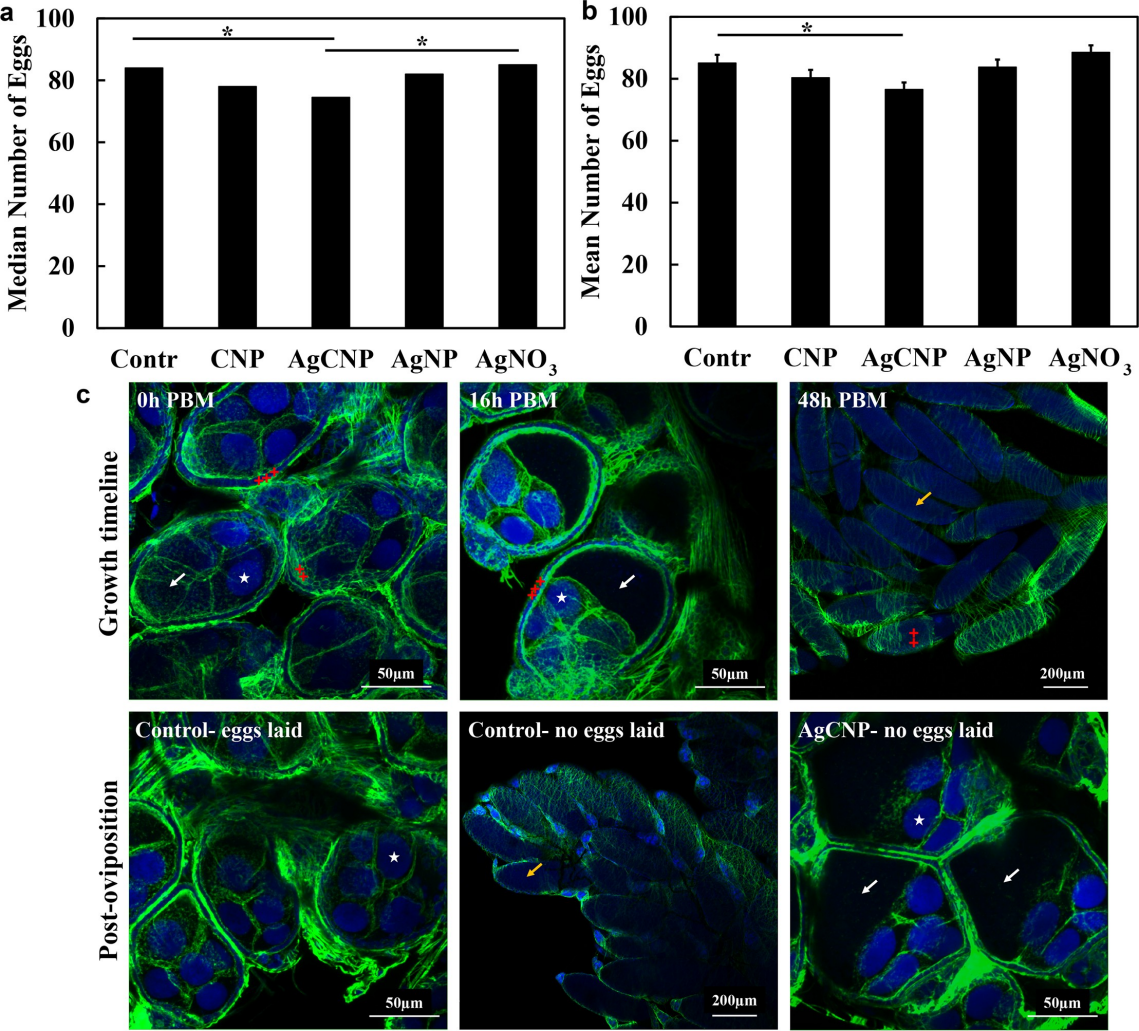
Effect of nanoceria exposure via blood meal on egg production of individual female *Ae*. *aegypti*. Nanoparticle-laced blood meals were fed to *Ae*. *aegypti* and well-fed mosquitoes were placed individually into separate oviposition cups. Egg clutches were counted and ovaries were imaged with confocal microscopy. (**a**) Median egg clutch sizes for individual mosquitoes; cups with zero (0) eggs were included in this graph and analysis. The median egg clutch of the AgCNP treatment group was significantly lower than those of the control and AgNO_3_ groups (p-values <0.05). (**b**) Mean egg clutch sizes for individual mosquitoes; cups with zero eggs were excluded from this graph and analysis. The mean egg clutch of the AgCNP-treated group was significantly lower compared to that of the control (p-value <0.05). (**c**) Representative confocal images of dissected ovaries stained with NucBlue (blue) and ActinGreen 488 (green); excitation lasers: NucBlue channel– 405nm, ActinGreen 488 channel– 488nm. Z-stacks were processed and overlaid using ImageJ. The upper image series shows a typical egg **growth timeline** from 0 to 48h post-blood meal (PBM) for control mosquitoes. Ovarioles at 0h PBM (maximum intensity z-projection image) are in the pre-vitellogenic stage with barely discernible oocytes (white arrow). Oocytes start accumulating vitellogenin resulting in increased size (white arrow, 16h PBM, maximum intensity z-projection image) while the nurse cells (white stars) are polarized in the direction of secondary follicles. At 48h PBM, fully-grown eggs are seen in the ovaries (yellow arrow) ready to be oviposited. The follicular cells (red crosses) start shedding at this point. The lower left image shows representative 5-days **post-oviposition** ovarioles of a control mosquito (eggs laid), complete with secondary follicles having entered the pre-vitellogenic stage. The lower middle image shows fully-developed eggs retained in an ovary from a representative control mosquito that laid no eggs. A representative maximum intensity z-projection image of ovarioles from an AgCNP-exposed mosquito is presented in the lower right panel and shows oocytes (white arrows) with arrested growth that appear similar in size to control ovarioles at approximately the 16h PBM timepoint. *: p-value <0.05. Solid lines indicate significantly different comparisons between median egg clutches in (**a**) and mean egg clutches in (**b**) in different treatment groups. Error bars in (**b**) = SEM. The experiment was repeated three (3) times, each with 25–30 cups of individual mosquitoes, yielding 83–90 cups in total for each experimental condition.

To study the effect of nanoceria on egg clutch for individual mosquitoes, multiple cups containing 50 female mosquitoes were fed warm blood meals laced with nanoparticles. Well-fed mosquitoes were then separated into individual cups containing germination paper for oviposition for an initial total of 90 mosquitoes for each treatment. Eggs on the germination cards were counted five days PBM to determine the egg clutch (**Fig 11A and 11B**). Some mosquitoes in each experimental condition oviposited zero (0) eggs: Control = 1, CNP = 3, AgCNP = 8, AgNP = 4, AgNO_3_ = 8. These zero-egg producing mosquitoes were included for Fig 11A, in which the median egg clutches are plotted and a nonparametric statistical analysis was performed. The median egg clutch of 74.5 eggs for the AgCNP group was significantly less than the median egg clutches of control and AgNO_3_ groups 84 and 85 eggs, respectively (p-values <0.05). In Fig 11B, the zero-egg producing mosquitoes were not included and parametric statistical analysis was performed. The mean clutch size of the AgCNP group was 76.5±2.28 eggs, which was significantly less than the mean control clutch size of 85.1±2.66 eggs (p-value <0.05). The maximum intensity z-projections of ovarioles/ovaries from mosquitoes which failed to oviposit showed a very interesting phenomenon occurring specifically in the ovarioles of AgCNP-exposed mosquitoes (**Fig 11C**). When the z-projections from those mosquitoes were compared to the z-projections of ovarioles at different time points in the egg-growth timeline of control mosquitoes, it was revealed that oocytes in the AgCNP-exposed mosquitoes appeared to have suffered a growth arrest by or before 16h PBM.

## Discussion

AgCNPs were synthesized and characterized to establish a chemically well-defined, mono-dispersion for use as a mosquito control agent. Materials characterizations (**Figs 1–4.**) show a homogeneous particle diameter, morphology, and putative chemical composition suggesting suitability for use as a biological reagent.

In addition to potential bioactive redox effects mediated by AgCNP-incorporated silver species, materials characterization also evidences several particle features which could confer strong bioactivity and chemical reactivity. These features include: presence of oxygen vacancy-related (point defect) highly redox-active Ce^3+^ species (37.4% Ce^3+^; **Fig 1.**), high topology surface character (under-coordination of surface atoms, dangling bonds; **Figs 2 and 3.**), and low grain size (greater crystallite surface area relative to volume and grain boundary volume as well as intra-granular stress fields and phonon confinement; **Figs 2–4.**). Many biomedical studies have determined Ce^3+^ content to be the principle component in nanoceria bioactivity. In future studies, we intend to investigate the relationship between AgCNP silver content and these Ce^3+^ sites as well as their influence on bioactivity. A putative relationship between these features is suggested by the low stability of silver in the cerium oxide lattice. This instability (i.e., high energy state) will necessarily have high reactivity (due to limited bond stabilization) and is evidenced in our study as the observed low-doping efficiency (~4%; experimental Ag_at%_: ~0.72, theoretical Ag_mol%_: 20, respectively) and is explained by the Hume-Rothery rules for solid solvent-solute solubility. Specifically, the significant difference in ionic size and valence between cerium and silver will limit silver incorporation. However, this inefficiency did not limit the nanoparticle utility in our pesticidal application. The behavior of nanoceria in agricultural/ecological studies is generally demonstrated as a protective against oxidative stress induced pathology; with a related preservation of native plant physiology. However, some studies have noted the modification of plant nutrient compositions and suggest additional studies into the long-term effects of these particles on the biological environment. Several studies have noted, however, the dissolution of CNPs in soils by organic ligands (redox-active species and moderate acids) [52–57]

Based on the XPS data, the total amount of silver in AgCNPs is calculated to be 0.22ppb, which is dramatically lower than the required concentration of 4.2ppm in the AgNO_3_ positive control (i.e., 2.7ppm free Ag^+^) for comparable activity. These data indicate an approximate four orders of magnitude increase in bioactivity for AgCNP over the Ag^+^ positive control. Additionally, the preliminary ICP-MS results indicate that free Ag^+^ in AgCNP suspensions is negligible. Together, these data suggest that the observed biological activity of AgCNP against *Ae*. *aegypti* does not result from free Ag^+^.

While exposure of 3^rd^ instar *Ae aegypti* larvae to AgCNPs in the presence and absence of larval food resulted in death, CNP exposure under these same conditions did not. The exact mode of larvicidal action of AgNPs, Ag^+^ and by extension AgCNPs is unknown. However, it has been asserted for AgNPs and Ag^+^ that this activity is due to irreversible binding of proteins by Ag^+^ resulting in the formation of complexes and protein denaturation through the reduction of Ag^+^ to metallic silver by the oxidizable groups of living tissue (**Chemical equation for Ag**^**+**^ **reduction reaction**) [16,58].

$${Ag}^{+}+e^{-}\overset{yields}{\to }{Ag}^{0}$$

**Chemical equation for Ag**^**+**^ **reduction reaction:** Reduction of silver ions to metallic silver. [58]

The observations for CNPs are contradictory to a previous report showing larvicidal activity from “green synthesized” cerium oxide nanoparticles [41]. The precise reasons for these contradictory results are unclear, but it is possible that the green synthesized nanoparticles have carbonaceous residues that contribute to the larvicidal activity presented in the Gopinath study. Also unclear from this previous study is if organic material, such as larval food, was present in the rearing water during the experimental time course. Here, we observed that the absence of food during the treatments accentuated the nanoceria larvicidal activity. Further study as to the mechanism of how larval food modulates AgCNP larvicidal activity is required but is beyond the scope of the present work. One possibility is that components of the food form mollifying organic “coronae” around AgCNPs [59]. No adulticidal effect was seen from any of the tested nanoceria however, AgCNPs reduced the production of eggs when administered in *Ae*. *aegypti* adult females through a blood meal. This difference in activity against adults and larvae suggest that larvae are more susceptible to the potential oxidative and/or fixative properties of AgCNPs.

Life history traits are commonly defined as the “size at birth; growth pattern; age and size at maturity; number, size, and sex of offspring; age-, stage- or size-specific reproductive effort; age-, stage- or size-specific rates of survival; and lifespan” [60]. Altering any number of these traits can have a profound effect on a population and as such they are ideal targets for measures which seek to control nuisance organisms such as vector mosquito species. Exposure of *Ae*. *aegypti* mosquitoes to nanoceria at both larval and adult stages changed one or more life history traits. Larvae and adults exposed to AgCNPs had significant reductions in the mass of eggs oviposited. When female mosquitoes with developed pre-vitellogenic ovarioles were exposed to AgCNPs, the resultant oviposited eggs hatched at a lower proportion compared to controls. The proportion of adults emerged from these hatched eggs was also significantly lower and thus provides another level of mosquito population control. Eggs oviposited by mosquitoes exposed to AgCNPs during the 3^rd^ instar had a significantly reduced egg-hatch proportion, which was most profound when larval food was withheld for 48h during the exposure. Given that the average egg masses normalized to the total numbers of corresponding female mosquitoes-emerged for both the control and AgCNP groups were similar, the reduction in egg-yield shown in Fig 7B for AgCNP exposure is likely due to the fact that there were fewer mosquitoes overall (i.e., fewer emerged) to lay eggs. Resultant progeny had similar body and wing sizes as well as sex ratios in essentially all experimental conditions. Noted exceptions were found only in the sex ratio and wing length data for CNP larval exposure with and without food, respectively. These results collectively indicate that nanoceria exposure during the 3^rd^ instar larval stage alters life history traits of *Ae*. *aegypti* to a greatest extent than adult exposure, which suggests differential impacts on the developing ovaries during this larval stage versus fully-mature ovaries at the pre-vitellogenic stage. The precise mechanisms underlying these observed significant differences are unknown and beyond the scope of the present studies, but could be due in part to alterations in gene expression, such as the ecdysone receptor, which has been shown to be affected by AgNP exposure in *Chironomus riparius* [61].

The developmental course and maturation of ovaries in mosquitoes are well-described in previous studies [62–64]. Briefly, ovary development begins in the 3^rd^ instar larval stage [62]. After emergence, the development continues until ovaries reach pre-vitellogenic arrest. Within two hours of a blood meal, the ovaries resume the developmental process by secretion of neural hormones called insulin-like peptides and ovary ecdysteroidogenic hormones [63,64]. Oocytes, which are barely discernible, begin to grow in size and uptake vitellogenin synthesized in the fat bodies. By 8h, ovarioles begin to polarize with the nurse cells moving towards the germarium and secondary follicles and the oocytes occupying the majority of space within the ovarioles. By 24h, the ovarioles start to elongate and accumulate yolk protein and, by 48h the oocytes have completely elongated. The chorion then forms, shedding of follicular cells begins; eggs are now ready to be fertilized and oviposited [62–64]. We observed that when *Ae*. *aegypti* females are exposed to AgCNPs via blood meal, oocytes of some mosquitoes undergo growth arrest—apparently ~16h PBM—that contributes to significant reduction in egg clutch. The cause(s) of this apparent growth arrest is unknown. Perhaps AgCNPs impact ecdysone receptor functions [64,65] and/or reactive oxygen species (ROS)-mediated signaling [66].

The reduction in egg clutch following AgCNPs exposure was variable suggesting some AgCNP batch-to-batch dependence. Though XPS data suggest the doping level of silver in AgCNPs is ~0.72at%, it is beyond any measurement methods to detect if silver doping is homogeneous throughout a given sample at the single/individual nanoparticle scale. Because of the surface properties of nanoceria and the nature of the oxidative dissolution-based chemical treatment/processing, it is possible that nanoparticle suspensions may have variance in dispersion, resulting in varying dosage of bioactive AgCNPs. Subsequent studies will seek to define and deconvolute dissolution kinetics of silver nanoparticles, nanoceria-incorporated silver micro-phases, and ceria surface etching processes to optimize the chemical treatment/processing towards producing homogenous, monodisperse solutions.

Pest control seeks to manage nuisance and medically important insects through various means such as chemical deployment and habitat management [67]. The focus of this study was to investigate the larvicidal and adulticidal potential of cerium oxide nanoparticles, both pure (CNPs) and silver-doped (AgCNPs), against *Ae*. *aegypti*, as well as the impacts of these nanoceria on life history traits including fecundity. Other such metal/metal oxide nanoparticles have previously shown promising insecticidal activity and chronic effects on reproduction and health of the progeny of exposed insects [20,68]. This study reports for the first time that exposure of *Ae*. *aegypti* females to AgCNPs via blood meal results in an apparent arrest of oocyte maturation in some mosquitoes and reduces the egg clutch overall. Further, it was found that AgCNPs exerted larvicidal activity, especially in the absence of organic matter (e.g., larval food) in the rearing water. In contrast to a previous study [41], CNPs showed no larvicidal activity and neither type of nanoceria demonstrated adulticidal activity. Importantly, the activities found for AgCNPs were not a result of free Ag^+^. Further, these novel nanoceria contained only ~0.22ppb Ag, which is significantly below the 0.1ppm listed secondary standard of the United States Environmental Protection Agency for drinking water [69] as well as the levels of Ag reported to exert desired effects on target insects (>1ppm) [70–72]. Given the findings of this study that the presence of food (i.e., organic matter) reduced the nanoparticle larvicidal activity, nanoceria such as AgCNPs would likely best be employed for larval control in environments with low organic material and/or in situations where repeated applications are feasible. Additionally, controlled-release formulations or devices that deliver agents in sustained manners over prolonged periods of time may also enable nanoceria larviciding strategies [73,74]. For approaches involving adult mosquitoes, toxic sugar baits [75,76] containing nanoceria could potentially be used to leverage the observed reduction in egg clutch resulting from AgCNP feeding. Overall, this study marks a significant development in mosquito control with further implications for pest biology, namely, the influence of inorganic nanoparticles on insect reproductive health and bioactivities beyond general metal toxicity-type effects.

## Supporting information

S1 Flowchart Experimental workflow for Figs 6–9. Red arrows indicate the listed subfigures of a given main figure. Black arrows indicate the sequential flow of mosquitoes and/or eggs from a given prior experiment/figure into the listed succeeding experiment/figure.
(PDF)

S1 Dataset Raw data file for experiments 6–11 and supplementary figure. (XLSX)

S1 Fig Adulticidal effects of nanoparticles on *Ae*. *aegypti* female mosquitoes. Adult *Ae*. *aegypti* females were fed sugar-based feeding solutions with varying concentrations of nanoceria. None of the mean proportions of the mosquitoes alive in any group were significantly different. Experiment was performed with 2–3 cups of 50 mosquitoes each for each experimental condition. Error bars = SEP.
(PDF)

S1 Calculations Molarity calculations for concentrations of nanoceria, numerical calculations for Ag^+^ in positive control and Ag content in AgCNPs. (PDF)

S1 File Statistical analysis for Figure in S1 Fig. (PDF)

S2 File Statistical analysis for Fig 6. (PDF)

S3 File Statistical analysis for Fig 7. (PDF)

S4 File Statistical analysis for Fig 8. (PDF)

S5 File Statistical analysis for Fig 9. (PDF)

S6 File Statistical analysis for Fig 10. (PDF)

S7 File Statistical analysis for Fig 11. (PDF)
