# Supplementary material for: Exposure to nanoceria impacts larval survival, life history traits and fecundity of Aedes aegypti
Source: PLoS Negl Trop Dis. 2020 Sep 25;14(9):e0008654. doi: 10.1371/journal.pntd.0008654 (PMC7540862; doi:10.1371/journal.pntd.0008654)

Experimental Flowchart: Experimental workflow for figures 6-9. Red arrows indicate the listed subfigures of a given main figure. Black arrows indicate the sequential flow of mosquitoes and/or eggs from a given prior experiment/figure into the listed succeeding experiment/figure.

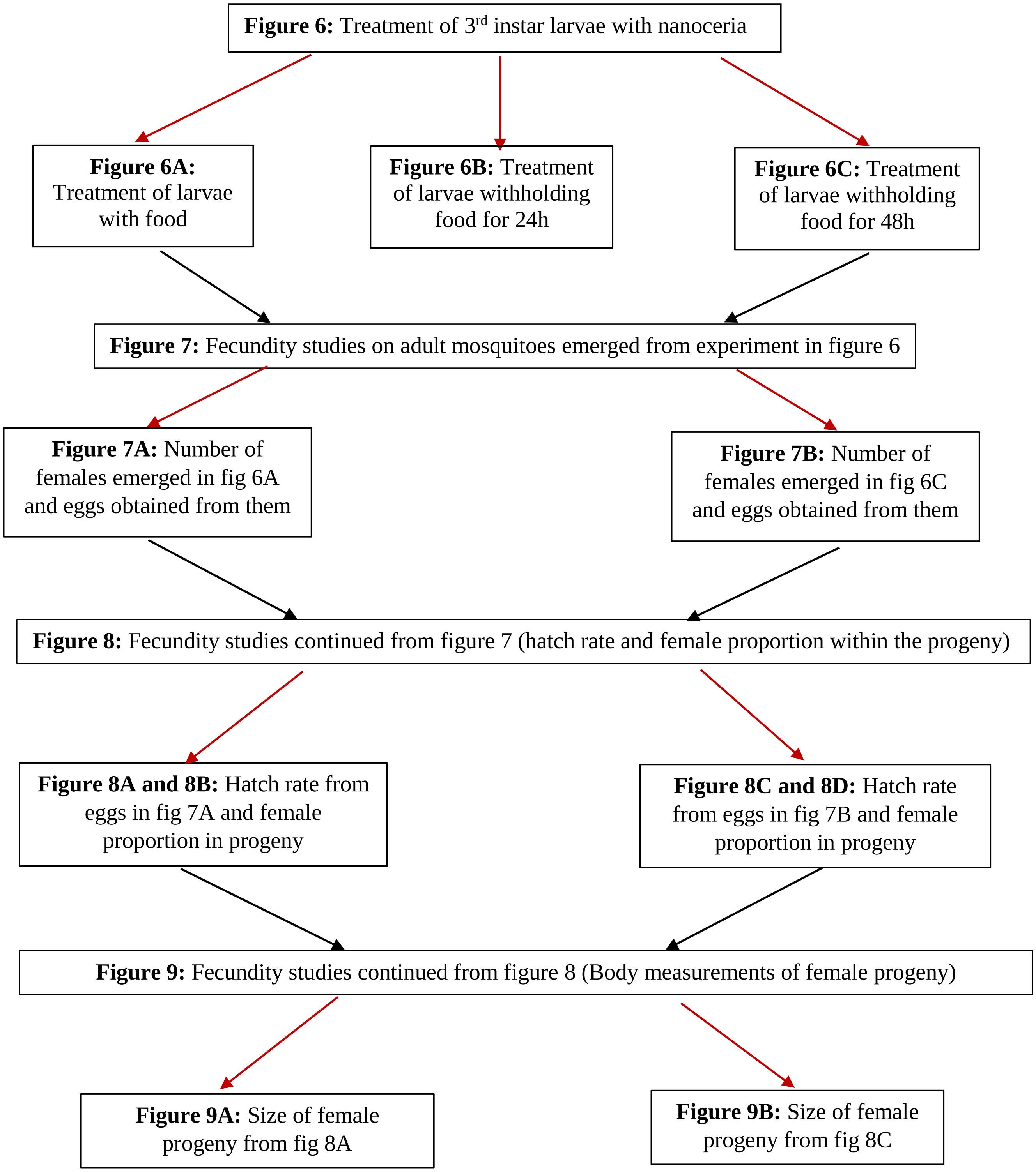

Supplement: S1 Flowchart — Red arrows indicate the listed subfigures of a given main figure. Black arrows indicate the sequential flow of mosquitoes and/or eggs from a given prior experiment/figure into the listed succeeding experiment/figure. (PDF) [file pntd.0008654.s001.pdf]
