# Supplementary material for: Exposure to nanoceria impacts larval survival, life history traits and fecundity of Aedes aegypti
Source: PLoS Negl Trop Dis. 2020 Sep 25;14(9):e0008654. doi: 10.1371/journal.pntd.0008654 (PMC7540862; doi:10.1371/journal.pntd.0008654)
Supplement: S1 Fig — Adult Ae. aegypti females were fed sugar-based feeding solutions with varying concentrations of nanoceria. None of the mean proportions of the mosquitoes alive in any group were significantly different. Experiment was performed with 2–3 cups of 50 mosquitoes each for each experimental condition. Error bars = SEP. (PDF) [file pntd.0008654.s003.pdf]

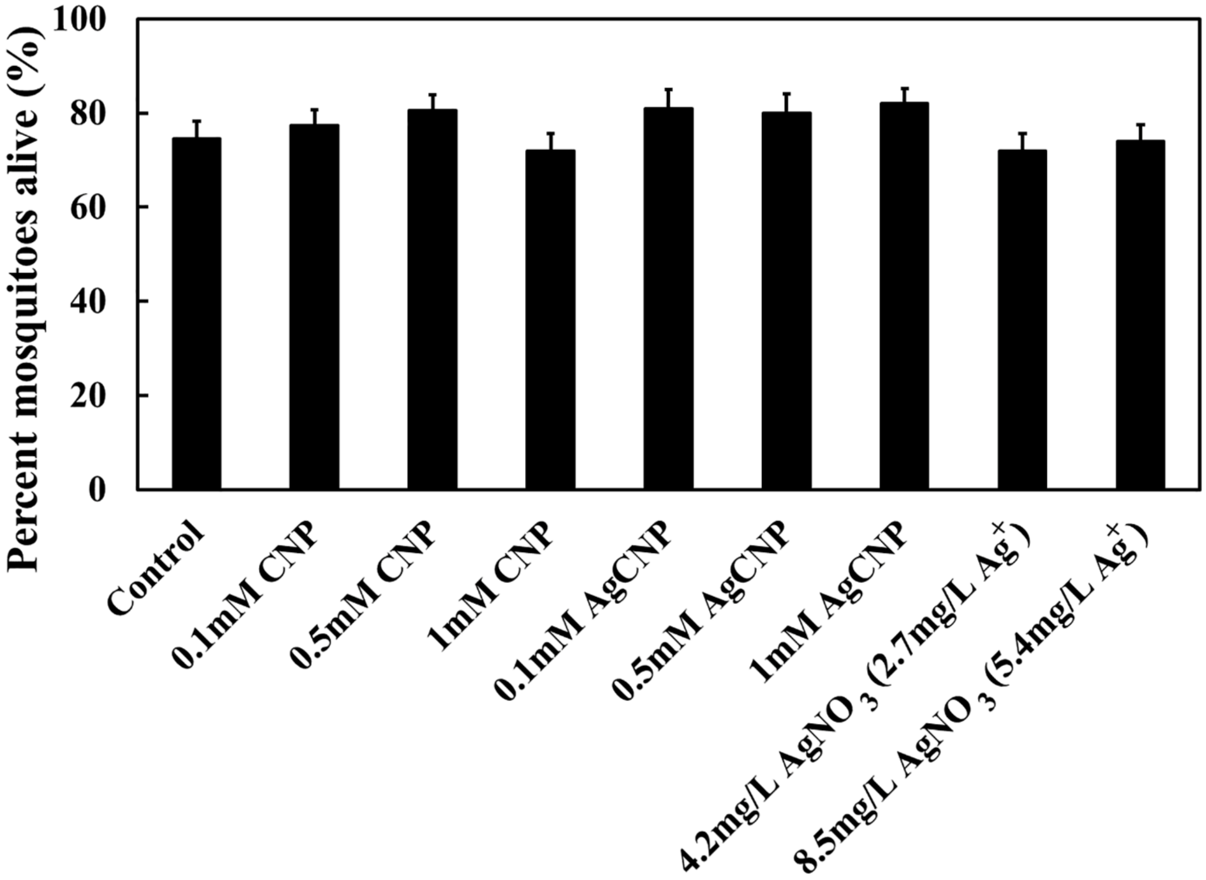

Adulticidal effects of nanoparticles on *Ae. aegypti* female mosquitoes. Adult *Ae. aegypti* females were fed sugar-based feeding solutions with varying concentrations of nanoceria. None of the mean proportions of the mosquitoes alive in any group were significantly different from each other. Experiment was performed with 2-3 cups of 50 mosquitoes each for each experimental condition. Error bars = SEP.
