# Supplementary material for: Exposure to nanoceria impacts larval survival, life history traits and fecundity of Aedes aegypti
Source: PLoS Negl Trop Dis. 2020 Sep 25;14(9):e0008654. doi: 10.1371/journal.pntd.0008654 (PMC7540862; doi:10.1371/journal.pntd.0008654)
Supplement: S1 Calculations — (PDF) [file pntd.0008654.s004.pdf]

Calculation:

1. Concentrations of CNPs:

The molarity of pure phase cerium oxide (CeO_2; molecular weight 172.11 g/mol) in 1 g/L was calculated as,

$$1\,\frac{g}{L} \div 172.11\,\frac{g}{mol} = 5.8 \times 10^{-3}\,\frac{moles}{L} = 5.8\,mM$$

From this concentration, dilutions were made for larval and adult experiments.

Larval experiment:

0.01 mM CNPs was used in larval experiments; the volume of stock CNP solution required to produce 40 mL (experimental volume):

$$(0.01\,mM \times 40\,mL) \div 5.8\,mM = 0.069\,mL$$

Adult experiment:

1 mM, 0.5 mM and 0.1 mM CNPs was used for adult experiments. Dilutions from stock concentration for these experiments were prepared as such:

a. 1mM CNPs

$$(1mM \times 1mL) \div 5.8\,mM = 0.172\,mL$$

Thus 0.172 mL of 5.8 mM CNPs was added to 0.828 mL water to make 1 mM solution

b. 0.5 mM CNPs

$$(0.5\,mM \times 1mL) \div 5.8\,mM = 0.086\,mL$$

Thus 0.086 mL of 5.8 mM CNPs was added to 0.914 mL water to make 0.5 mM solution

c. 0.1 mM CNPs

$$(0.1\,mM \times 1mL) \div 5.8\,mM = 0.0172\,mL$$

Thus 0.0172 mL of 5.8 mM CNPs was added to 0.9828 mL water to make 0.1 mM solution

2. Concentrations of $AgNO_3$:

Larval experiment:

Firstly, a stock solution of 1 mM $AgNO_3$ was produced as:

$$169.87\,\frac{g}{mol} \times 0.001\,\frac{mol}{L} \times 1\,mL = 0.170\,g\;(AgNO_3)\,|$$

0.025 mM $AgNO_3$ was used in larval experiments and produced by dilution from stock:

$$(0.025\,mM \times 40\,mL) \div 1\,mM = 1\,mL$$

1 mL of 1 mM $AgNO_3$ was added to 39 mL water to make 0.025 mM $AgNO_3$,

Calculating mg/L (ppm) of silver in 0.025 mM $AgNO_3$:

Molecular weight of $AgNO_3$ is 169.87 g/mol; so 1 mM $AgNO_3$ has 169.87 µg/mL of $AgNO_3$

$$0.025 \times 10^{-3}\ \frac{mol\ AgNO_3}{L} \times \frac{1\ mol\ Ag}{1\ mol\ AgNO_3} \times 169.87 \frac{g\ Ag}{mol\ Ag} = 4.24\ \frac{mg}{L}\ or\ ppm$$

The atomic weight of Ag is 107.87 g/mol; so 1 mM has 107.87 µg/mL

$$0.025 \times 10^{-3}\ \frac{mol\ AgNO_3}{L} \times \frac{1\ mol\ Ag}{1\ mol\ AgNO_3} \times 107.87 \frac{g\ Ag}{mol\ Ag} = 2.697\ \frac{mg}{L}\ or\ ppm$$

Adult experiment:

a. 0.025 mM $AgNO_3$

$$(0.025\ mM \times 1\ mL) \div 1\ mM = 0.025\ mL$$

0.025 mL of 1 mM $AgNO_3$ was added to 0.975 mL blood or sugar solution to make 0.025 mM $AgNO_3$,

Per the above calculation, 0.025 mM equates to 4.24 ppm of $AgNO_3$ or 2.697 ppm Ag

b. 0.050 mM $AgNO_3$

$$(0.050\ mM \times 1\ mL) \div 1\ mM = 0.050\ mL$$

0.050 mL of 1 mM $AgNO_3$ was added to 0.950 mL blood or sugar solution to make 0.050 mM $AgNO_3$,

Calculating mg/L (ppm) of silver in 0.050 mM $AgNO_3$ leads to twice the concentration calculated for 0.025 mM (8.48 ppm $AgNO_3$ or 5.394 ppm Ag).

3. Calculation of amount of Ag in doped nanoparticles:

XPS showed that 0.72 atomic percent (at%) of Ag is incorporated in AgCNPs (per Avantage software). The ratio of Ce^{3+} to Ce^{4+} redox states in each formulation was determined analytically. First, a baseline correction was fit to the data using a *smart* baseline function (Avantage). Next, peaks were fit using curves constrained by state-specific properties (e.g. separation between multiplet spin states, relative peak heights for associated peaks split due to spin-orbit coupling). Fitting was optimized by least squares regression (Avantage) and the peaks associated with Ce^{3+} and Ce^{4+} were integrated numerically. Sum peak contributions for each state were then normalized as,

$$[Ce^{3+}] = \frac{\sum Ce^{3+}}{\sum Ce^{3+} + \sum Ce^{4+}} = \frac{\sum(v_0 + v' + u_0 + u')}{\sum[(v_0 + v' + u_0 + u') + (v + v'' + v''' + u + u'' + u''')]}$$

Where 'v' peaks are related to emissions from the Ce3d5/2 and 'u' peaks to Ce3d3/2 multiplets respectively, per literature convention, along with associated satellite peaks (arising from complex, final state emissions). The average Ce^{3+}/Ce^{4+} ratio for the silver-modified ceria formulation was determined as 37.4%. Atomic percent silver (produced using Avantage analysis software, with respect to spectral contributions from O, Ag, and Ce) and relative Ce^{3+} content were subsequently used in describing the silver content per mass of nanomaterial.

We consider cerium and oxygen percentages as arising from proportions of the oxide in each redox state in fixed molar ratios, weighted by the fraction of each cerium redox state. Mass percents of silver were thereby calculated from the molarities of total atom proportions:

$$0.72\ at\%\ Ag \rightarrow 99.28\% \sum(Ce\% + O\%)$$

Given the percent of cerium and oxygen represents contributions from (37.4%) Ce_2O_3 and (62.6%) CeO_2 stoichiometries,

$$\frac{0.72}{0.626(33.09 + 66.18) + 0.374(39.712 + 59.568) + 0.72} = \frac{0.72\ atoms\ of Ag}{100\ atoms\ of\ 'NP'}$$

Converting atomic masses of Ag {107.87}, Ce {140.0}, and O {16.0 g/mol} allows conversion of atomic to mass ratio as:

$$\frac{0.72\{107.87\}}{0.626(33.09\{140.0\} + 66.18\{16.0\}) + 0.374(39.712\{140.0\} + 59.568\{16\}) + 0.72\{107.87\}} = \frac{0.0128\ g\ Ag}{100\ g\ 'NP'}$$

Lastly, using the stock concentration of nanoparticles and the experimental dilution factor (5.8 mM → 0.01 mM: 1.72×10^{-3}) noted in section1:

$$\frac{1\ g\ 'NP'}{1\ L} \times 1.72 \times 10^{-3} \times \frac{0.0128\ g\ Ag}{100\ g\ 'NP'} = \frac{2.2 \times 10^{-7} g}{L} = \frac{0.22 \mu g}{L} = 0.22\ ppb\ Ag$$
