# Supplementary material for: Exposure to nanoceria impacts larval survival, life history traits and fecundity of Aedes aegypti
Source: PLoS Negl Trop Dis. 2020 Sep 25;14(9):e0008654. doi: 10.1371/journal.pntd.0008654 (PMC7540862; doi:10.1371/journal.pntd.0008654)
Supplement: S1 File — (PDF) [file pntd.0008654.s005.pdf]

Figure supplementary 1-% Adult Mosquitos Alive;

The FREQ Procedure

Table of ALIVE by GROUP

Frequency<br>Col Pct	ALIVE	GROUP									
		0.1mM_AgCNP-1-10-T	0.1mM_CNP-T	0.5mM_AgCNP-1-10-T	0.5mM_CNP-T	1mM_AgCNP-1-10-T	1mM_CNP-T	4mg/L_AgNO3	8mg/L_AgNO3	Control	Total
	**Yes**	81<br>81.00	116<br>77.33	80<br>80.00	121<br>80.67	123<br>82.00	108<br>72.00	108<br>72.00	111<br>74.00	112<br>74.67	960
	**No**	19<br>19.00	34<br>22.67	20<br>20.00	29<br>19.33	27<br>18.00	42<br>28.00	42<br>28.00	39<br>26.00	38<br>25.33	290
	**Total**	100	150	100	150	150	150	150	150	150	1250

Statistics for Table of ALIVE by GROUP

Statistic	DF	Value	Prob
**Chi-Square**	8	10.0462	0.2618
**Likelihood Ratio Chi-Square**	8	10.1105	0.2574
**Mantel-Haenszel Chi-Square**	1	4.3478	0.0371
**Phi Coefficient**		0.0896	
**Contingency Coefficient**		0.0893	
**Cramer's V**		0.0896	

Proportion Alive with SE by Group

The FREQ Procedure

GROUP=0.1mM_AgCNP-1-10-T

ALIVE	Frequency	Percent	Cumulative Frequency	Cumulative Percent
**Yes**	81	81.00	81	81.00
**No**	19	19.00	100	100.00

Binomial Proportion	
**ALIVE = Yes**	
**Proportion**	0.8100
**ASE**	0.0392
**95% Lower Conf Limit**	0.7331
**95% Upper Conf Limit**	0.8869
**Exact Conf Limits**	
**95% Lower Conf Limit**	0.7193
**95% Upper Conf Limit**	0.8816

Test of H0: Proportion = 0.5	
**ASE under H0**	0.0500
**Z**	6.2000
**One-sided Pr > Z**	<.0001
**Two-sided Pr > |Z|**	<.0001

Sample Size = 100

Proportion Alive with SE by Group

The FREQ Procedure
GROUP=0.1mM_CNP-T

ALIVE	Frequency	Percent	Cumulative Frequency	Cumulative Percent
Yes	116	77.33	116	77.33
No	34	22.67	150	100.00

Binomial Proportion	
ALIVE = Yes	
Proportion	0.7733
ASE	0.0342
95% Lower Conf Limit	0.7063
95% Upper Conf Limit	0.8403
Exact Conf Limits	
95% Lower Conf Limit	0.6979
95% Upper Conf Limit	0.8376

Test of H0: Proportion = 0.5	
ASE under H0	0.0408
Z	6.6953
One-sided Pr > Z	<.0001
Two-sided Pr > |Z|	<.0001

Sample Size = 150

Proportion Alive with SE by Group

The FREQ Procedure
GROUP=0.5mM_AgCNP-1-10-T

ALIVE	Frequency	Percent	Cumulative Frequency	Cumulative Percent
Yes	80	80.00	80	80.00
No	20	20.00	100	100.00

Binomial Proportion

ALIVE = Yes	
Proportion	0.8000
ASE	0.0400
95% Lower Conf Limit	0.7216
95% Upper Conf Limit	0.8784
**Exact Conf Limits**	
95% Lower Conf Limit	0.7082
95% Upper Conf Limit	0.8733

Test of H0: Proportion = 0.5	
ASE under H0	0.0500
Z	6.0000
One-sided Pr > Z	<.0001
Two-sided Pr > |Z|	<.0001

Sample Size = 100

Proportion Alive with SE by Group

The FREQ Procedure
GROUP=0.5mM_CNP-T

ALIVE	Frequency	Percent	Cumulative Frequency	Cumulative Percent
**Yes**	121	80.67	121	80.67
**No**	29	19.33	150	100.00

Binomial Proportion	
**ALIVE = Yes**	
**Proportion**	0.8067
**ASE**	0.0322
**95% Lower Conf Limit**	0.7435
**95% Upper Conf Limit**	0.8699
**Exact Conf Limits**	
**95% Lower Conf Limit**	0.7343
**95% Upper Conf Limit**	0.8665

Test of H0: Proportion = 0.5	
**ASE under H0**	0.0408
**Z**	7.5118
**One-sided Pr > Z**	<.0001
**Two-sided Pr > |Z|**	<.0001

Sample Size = 150

Proportion Alive with SE by Group

The FREQ Procedure
GROUP=1mM_AgCNP-1-10-T

ALIVE	Frequency	Percent	Cumulative Frequency	Cumulative Percent
**Yes**	123	82.00	123	82.00
**No**	27	18.00	150	100.00

Binomial Proportion	
**ALIVE = Yes**	
**Proportion**	0.8200
**ASE**	0.0314
**95% Lower Conf Limit**	0.7585
**95% Upper Conf Limit**	0.8815
**Exact Conf Limits**	
**95% Lower Conf Limit**	0.7490
**95% Upper Conf Limit**	0.8779

Test of H0: Proportion = 0.5	
**ASE under H0**	0.0408
**Z**	7.8384
**One-sided Pr > Z**	<.0001
**Two-sided Pr > |Z|**	<.0001

Sample Size = 150

Proportion Alive with SE by Group

The FREQ Procedure
GROUP=1mM_CNP-T

ALIVE	Frequency	Percent	Cumulative Frequency	Cumulative Percent
Yes	108	72.00	108	72.00
No	42	28.00	150	100.00

Binomial Proportion	
ALIVE = Yes	
Proportion	0.7200
ASE	0.0367
95% Lower Conf Limit	0.6481
95% Upper Conf Limit	0.7919
Exact Conf Limits	
95% Lower Conf Limit	0.6409
95% Upper Conf Limit	0.7902

Test of H0: Proportion = 0.5	
ASE under H0	0.0408
Z	5.3889
One-sided Pr > Z	<.0001
Two-sided Pr > |Z|	<.0001

Sample Size = 150

Proportion Alive with SE by Group

The FREQ Procedure
GROUP=4mg/L_AgNO3

ALIVE	Frequency	Percent	Cumulative Frequency	Cumulative Percent
Yes	108	72.00	108	72.00
No	42	28.00	150	100.00

Binomial Proportion	
ALIVE = Yes	
Proportion	0.7200
ASE	0.0367
95% Lower Conf Limit	0.6481
95% Upper Conf Limit	0.7919
Exact Conf Limits	
95% Lower Conf Limit	0.6409
95% Upper Conf Limit	0.7902

Test of H0: Proportion = 0.5	
ASE under H0	0.0408
Z	5.3889
One-sided Pr > Z	<.0001
Two-sided Pr > |Z|	<.0001

Sample Size = 150

Proportion Alive with SE by Group

The FREQ Procedure
GROUP=8mg/L_AgNO3

ALIVE	Frequency	Percent	Cumulative Frequency	Cumulative Percent
Yes	111	74.00	111	74.00
No	39	26.00	150	100.00

Binomial Proportion	
ALIVE = Yes	
Proportion	0.7400
ASE	0.0358
95% Lower Conf Limit	0.6698
95% Upper Conf Limit	0.8102
Exact Conf Limits	
95% Lower Conf Limit	0.6621
95% Upper Conf Limit	0.8081

Test of H0: Proportion = 0.5	
ASE under H0	0.0408
Z	5.8788
One-sided Pr > Z	<.0001
Two-sided Pr > |Z|	<.0001

Sample Size = 150

Proportion Alive with SE by Group

The FREQ Procedure
GROUP=Control

ALIVE	Frequency	Percent	Cumulative Frequency	Cumulative Percent
**Yes**	112	74.67	112	74.67
**No**	38	25.33	150	100.00

Binomial Proportion	
**ALIVE = Yes**	
**Proportion**	0.7467
**ASE**	0.0355
**95% Lower Conf Limit**	0.6771
**95% Upper Conf Limit**	0.8163
**Exact Conf Limits**	
**95% Lower Conf Limit**	0.6693
**95% Upper Conf Limit**	0.8141

Test of H0: Proportion = 0.5	
**ASE under H0**	0.0408
**Z**	6.0421
**One-sided Pr > Z**	<.0001
**Two-sided Pr > |Z|**	<.0001

Sample Size = 150
