# Supplementary material for: Exposure to nanoceria impacts larval survival, life history traits and fecundity of Aedes aegypti
Source: PLoS Negl Trop Dis. 2020 Sep 25;14(9):e0008654. doi: 10.1371/journal.pntd.0008654 (PMC7540862; doi:10.1371/journal.pntd.0008654)
Supplement: S2 File — (PDF) [file pntd.0008654.s006.pdf]

Figure 6a- % Adults Emerged by Group;

Chi-Square Analysis for a 2x4 Figure

The FREQ Procedure

Figure of EMERGED by GROUP

Frequency / Col Pct	EMERGED	GROUP				
		Control	CNP	AgCNP	AgNO3	Total
	Yes	291	269	225	0	785
		88.18	81.52	68.18	0.00	
	No	39	61	105	330	535
		11.82	18.48	31.82	100.00	
	Total	330	330	330	330	1320

Statistics for Figure of EMERGED by GROUP

Statistic	DF	Value	Prob
Chi-Square	3	674.0038	<.0001
Likelihood Ratio Chi-Square	3	813.7595	<.0001
Mantel-Haenszel Chi-Square	1	528.1898	<.0001
Phi Coefficient		0.7146	
Contingency Coefficient		0.5814	
Cramer's V		0.7146	

Sample Size = 1320

Proportion Emerged with SE by Group

The FREQ Procedure
GROUP=1=Contro

EMERGED	Frequency	Percent	Cumulative Frequency	Cumulative Percent
Yes	291	88.18	291	88.18
No	39	11.82	330	100.00

Binomial Proportion	
EMERGED = Yes	
Proportion	0.8818
ASE	0.0178
95% Lower Conf Limit	0.8470
95% Upper Conf Limit	0.9166
Exact Conf Limits	
95% Lower Conf Limit	0.8420
95% Upper Conf Limit	0.9146

Test of H0: Proportion = 0.5	
ASE under H0	0.0275
Z	13.8721
One-sided Pr > Z	<.0001
Two-sided Pr > |Z|	<.0001

Sample Size = 330

Proportion Emerged with SE by Group

The FREQ Procedure
GROUP=2=CNP

EMERGED	Frequency	Percent	Cumulative Frequency	Cumulative Percent
**Yes**	269	81.52	269	81.52
**No**	61	18.48	330	100.00

Binomial Proportion	
**EMERGED = Yes**	
**Proportion**	0.8152
**ASE**	0.0214
**95% Lower Conf Limit**	0.7733
**95% Upper Conf Limit**	0.8570
**Exact Conf Limits**	
**95% Lower Conf Limit**	0.7690
**95% Upper Conf Limit**	0.8556

Test of H0: Proportion = 0.5	
**ASE under H0**	0.0275
**Z**	11.4500
**One-sided Pr > Z**	<.0001
**Two-sided Pr > |Z|**	<.0001

Sample Size = 330

Proportion Emerged with SE by Group

The FREQ Procedure
GROUP=3=AgCNP

EMERGED	Frequency	Percent	Cumulative Frequency	Cumulative Percent
Yes	225	68.18	225	68.18
No	105	31.82	330	100.00

Binomial Proportion	
EMERGED = Yes	
Proportion	0.6818
ASE	0.0256
95% Lower Conf Limit	0.6316
95% Upper Conf Limit	0.7321
Exact Conf Limits	
95% Lower Conf Limit	0.6286
95% Upper Conf Limit	0.7318

Sample Size = 330

Proportion Emerged with SE by Group

The FREQ Procedure
GROUP=4=AgNO3

EMERGED	Frequency	Percent	Cumulative Frequency	Cumulative Percent
**No**	330	100.00	330	100.00

Binomial Proportion	
**EMERGED = No**	
**Proportion**	1.0000
**ASE**	0.0000
**95% Lower Conf Limit**	1.0000
**95% Upper Conf Limit**	1.0000
**Exact Conf Limits**	
**95% Lower Conf Limit**	0.9889
**95% Upper Conf Limit**	1.0000

Sample Size = 330

Chi-Square Analysis for a 2x4 Figure

Tukey Style Multiple Comparisons of Proportions

Compare	Diff	SE	q	q(.05)	Conclude
1 vs 4	67.65	1.58	42.82	3.633	R
1 vs 3	14.17	1.58	8.97	3.633	R
1 vs 2	5.33	1.58	3.37	3.633	A
2 vs 4	62.33	1.58	39.45	3.633	R
2 vs 3	8.84	1.58	5.59	3.633	R
3 vs 4	53.49	1.58	33.85	3.633	R

Reference: Biostatistical Analysis, Fourth Edition, Jerrold Zar, 1999, p564.

Tukey Style Multiple Comparisons of Proportions

Compare	Diff	SE	q	q(.01)	Conclude
1 vs 4	67.65	1.58	42.82	4.403	R
1 vs 3	14.17	1.58	8.97	4.403	R
1 vs 2	5.33	1.58	3.37	4.403	A
2 vs 4	62.33	1.58	39.45	4.403	R
2 vs 3	8.84	1.58	5.59	4.403	R
3 vs 4	53.49	1.58	33.85	4.403	R

Reference: Biostatistical Analysis, Fourth Edition, Jerrold Zar, 1999, p564.

Tukey Style Multiple Comparisons of Proportions

Compare	Diff	SE	q	q(.001)	Conclude
1 vs 4	67.65	1.58	42.82	5.309	R
1 vs 3	14.17	1.58	8.97	5.309	R
1 vs 2	5.33	1.58	3.37	5.309	A
2 vs 4	62.33	1.58	39.45	5.309	R
2 vs 3	8.84	1.58	5.59	5.309	R
3 vs 4	53.49	1.58	33.85	5.309	R

Reference: Biostatistical Analysis, Fourth Edition, Jerrold Zar, 1999, p564.

Figure 6a- % Larvae dead by Group;

Chi-Square Analysis for a 2x4 Figure

The FREQ Procedure

Frequency<br>Col Pct	Figure of LARVAE_DEAD by GROUP					
	LARVAE_DEAD	GROUP				
		Control	CNP	AgCNP	AgNO3	Total
	Yes	22<br>6.67	45<br>13.64	83<br>25.15	269<br>81.52	419
	No	308<br>93.33	285<br>86.36	247<br>74.85	61<br>18.48	901
	Total	330	330	330	330	1320

Statistics for Figure of LARVAE_DEAD by GROUP

Statistic	DF	Value	Prob
Chi-Square	3	529.6343	<.0001
Likelihood Ratio Chi-Square	3	537.0773	<.0001
Mantel-Haenszel Chi-Square	1	424.0440	<.0001
Phi Coefficient		0.6334	
Contingency Coefficient		0.5351	
Cramer's V		0.6334	

Sample Size = 1320

Larvae Dead with SE by Group

The FREQ Procedure
GROUP=1=Contro

LARVAE_DEAD	Frequency	Percent	Cumulative Frequency	Cumulative Percent
**Yes**	22	6.67	22	6.67
**No**	308	93.33	330	100.00

Binomial Proportion	
**LARVAE_DEAD = Yes**	
**Proportion**	0.0667
**ASE**	0.0137
**95% Lower Conf Limit**	0.0398
**95% Upper Conf Limit**	0.0936
**Exact Conf Limits**	
**95% Lower Conf Limit**	0.0422
**95% Upper Conf Limit**	0.0992

Test of H0: Proportion = 0.5	
**ASE under H0**	0.0275
**Z**	-15.7438
**One-sided Pr < Z**	<.0001
**Two-sided Pr > |Z|**	<.0001

Sample Size = 330

Larvae Dead with SE by Group

The FREQ Procedure
GROUP=2=CNP

LARVAE_DEAD	Frequency	Percent	Cumulative Frequency	Cumulative Percent
**Yes**	45	13.64	45	13.64
**No**	285	86.36	330	100.00

Binomial Proportion	
**LARVAE_DEAD = Yes**	
**Proportion**	0.1364
**ASE**	0.0189
**95% Lower Conf Limit**	0.0993
**95% Upper Conf Limit**	0.1734
**Exact Conf Limits**	
**95% Lower Conf Limit**	0.1012
**95% Upper Conf Limit**	0.1782

Test of H0: Proportion = 0.5	
**ASE under H0**	0.0275
**Z**	-13.2116
**One-sided Pr < Z**	<.0001
**Two-sided Pr > |Z|**	<.0001

Sample Size = 330

Larvae Dead with SE by Group

The FREQ Procedure
GROUP=3=AgCNP

LARVAE_DEAD	Frequency	Percent	Cumulative Frequency	Cumulative Percent
**Yes**	83	25.15	83	25.15
**No**	247	74.85	330	100.00

Binomial Proportion	
**LARVAE_DEAD = Yes**	
**Proportion**	0.2515
**ASE**	0.0239
**95% Lower Conf Limit**	0.2047
**95% Upper Conf Limit**	0.2983
**Exact Conf Limits**	
**95% Lower Conf Limit**	0.2056
**95% Upper Conf Limit**	0.3019

Test of H0: Proportion = 0.5	
**ASE under H0**	0.0275
**Z**	-9.0279
**One-sided Pr < Z**	<.0001
**Two-sided Pr > |Z|**	<.0001

Sample Size = 330

Larvae Dead with SE by Group

The FREQ Procedure
GROUP=4=AgNO3

LARVAE_DEAD	Frequency	Percent	Cumulative Frequency	Cumulative Percent
Yes	269	81.52	269	81.52
No	61	18.48	330	100.00

Binomial Proportion	
LARVAE_DEAD = Yes	
Proportion	0.8152
ASE	0.0214
95% Lower Conf Limit	0.7733
95% Upper Conf Limit	0.8570
Exact Conf Limits	
95% Lower Conf Limit	0.7690
95% Upper Conf Limit	0.8556

Test of H0: Proportion = 0.5	
ASE under H0	0.0275
Z	11.4500
One-sided Pr > Z	<.0001
Two-sided Pr > |Z|	<.0001

Sample Size = 330

Chi-Square Analysis for a 2x4 Figure

Tukey Style Multiple Comparisons of Proportions

Compare	Diff	SE	q	q(.05)	Conclude
4 vs 1	49.36	1.58	31.24	3.633	R
4 vs 2	42.71	1.58	27.03	3.633	R
4 vs 3	34.32	1.58	21.72	3.633	R
3 vs 1	15.04	1.58	9.52	3.633	R
3 vs 2	8.39	1.58	5.31	3.633	R
2 vs 1	6.65	1.58	4.21	3.633	R

Reference: Biostatistical Analysis, Fourth Edition, Jerrold Zar, 1999, p564.

Chi-Square Analysis for a 2x4 Figure

Tukey Style Multiple Comparisons of Proportions

Compare	Diff	SE	q	q(.01)	Conclude
4 vs 1	49.36	1.58	31.24	4.403	R
4 vs 2	42.71	1.58	27.03	4.403	R
4 vs 3	34.32	1.58	21.72	4.403	R
3 vs 1	15.04	1.58	9.52	4.403	R
3 vs 2	8.39	1.58	5.31	4.403	R
2 vs 1	6.65	1.58	4.21	4.403	A

Reference: Biostatistical Analysis, Fourth Edition, Jerrold Zar, 1999, p564.

Chi-Square Analysis for a 2x4 Figure

Tukey Style Multiple Comparisons of Proportions

Compare	Diff	SE	q	q(.001)	Conclude
4 vs 1	49.36	1.58	31.24	5.309	R
4 vs 2	42.71	1.58	27.03	5.309	R
4 vs 3	34.32	1.58	21.72	5.309	R
3 vs 1	15.04	1.58	9.52	5.309	R
3 vs 2	8.39	1.58	5.31	5.309	R
2 vs 1	6.65	1.58	4.21	5.309	A

Reference: Biostatistical Analysis, Fourth Edition, Jerrold Zar, 1999, p564.

Figure 6b- % Adults Emerged by Group;

Chi-Square Analysis for a 2x4 Figure

The FREQ Procedure

Frequency<br>Col Pct	EMERGED	GROUP					
		1=Control_with	2=Control_without	3=CNP	4=AgCNP	5=AgNO3	Total
	Yes	192	150	192	37	0	571
		91.43	71.43	91.43	17.62	0.00	
	No	18	60	18	173	210	479
		8.57	28.57	8.57	82.38	100.00	
	Total	210	210	210	210	210	1050

Figure of EMERGED by GROUP

Statistics for Figure of EMERGED by GROUP

Statistic	DF	Value	Prob
Chi-Square	4	621.7024	<.0001

Sample Size = 1050

Adults Emerged with SE by Group

The FREQ Procedure
GROUP=1=Control_with

EMERGED	Frequency	Percent	Cumulative Frequency	Cumulative Percent
Yes	192	91.43	192	91.43

EMERGED	Frequency	Percent	Cumulative Frequency	Cumulative Percent
No	18	8.57	210	100.00

Binomial Proportion	
EMERGED = Yes	
Proportion	0.9143
ASE	0.0193
95% Lower Conf Limit	0.8764
95% Upper Conf Limit	0.9521
Exact Conf Limits	
95% Lower Conf Limit	0.8679
95% Upper Conf Limit	0.9484

Test of H0: Proportion = 0.5	
ASE under H0	0.0345
Z	12.0071
One-sided Pr > Z	<.0001
Two-sided Pr > |Z|	<.0001

Sample Size = 210

Adults Emerged with SE by Group

The FREQ Procedure
GROUP=2=Control_without

EMERGED	Frequency	Percent	Cumulative Frequency	Cumulative Percent
Yes	150	71.43	150	71.43
No	60	28.57	210	100.00

Binomial Proportion	
EMERGED = Yes	
Proportion	0.7143
ASE	0.0312
95% Lower Conf Limit	0.6532
95% Upper Conf Limit	0.7754
Exact Conf Limits	
95% Lower Conf Limit	0.6481
95% Upper Conf Limit	0.7743

Test of H0: Proportion = 0.5	
ASE under H0	0.0345
Z	6.2106
One-sided Pr > Z	<.0001
Two-sided Pr > |Z|	<.0001

Sample Size = 210

Adults Emerged with SE by Group

The FREQ Procedure
GROUP=3=CNP

EMERGED	Frequency	Percent	Cumulative Frequency	Cumulative Percent
**Yes**	192	91.43	192	91.43
**No**	18	8.57	210	100.00

Binomial Proportion	
**EMERGED = Yes**	
**Proportion**	0.9143
**ASE**	0.0193
**95% Lower Conf Limit**	0.8764
**95% Upper Conf Limit**	0.9521
**Exact Conf Limits**	
**95% Lower Conf Limit**	0.8679
**95% Upper Conf Limit**	0.9484

Test of H0: Proportion = 0.5	
**ASE under H0**	0.0345
**Z**	12.0071
**One-sided Pr > Z**	<.0001
**Two-sided Pr > |Z|**	<.0001

Sample Size = 210

Adults Emerged with SE by Group

The FREQ Procedure
GROUP=4=AgCNP

EMERGED	Frequency	Percent	Cumulative Frequency	Cumulative Percent
Yes	37	17.62	37	17.62
No	173	82.38	210	100.00

Binomial Proportion	
EMERGED = Yes	
Proportion	0.1762
ASE	0.0263
95% Lower Conf Limit	0.1247
95% Upper Conf Limit	0.2277
Exact Conf Limits	
95% Lower Conf Limit	0.1272
95% Upper Conf Limit	0.2346

Test of H0: Proportion = 0.5	
ASE under H0	0.0345
Z	-9.3849
One-sided Pr < Z	<.0001
Two-sided Pr > |Z|	<.0001

Sample Size = 210

Adults Emerged with SE by Group

The FREQ Procedure
GROUP=5=AgNO3

EMERGED	Frequency	Percent	Cumulative Frequency	Cumulative Percent
**No**	210	100.00	210	100.00

Binomial Proportion	
**EMERGED = No**	
**Proportion**	1.0000
**ASE**	0.0000
**95% Lower Conf Limit**	1.0000
**95% Upper Conf Limit**	1.0000
**Exact Conf Limits**	
**95% Lower Conf Limit**	0.9826
**95% Upper Conf Limit**	1.0000

Test of H0: Proportion = 0.5	
**ASE under H0**	0.0345
**Z**	14.4914
**One-sided Pr > Z**	<.0001
**Two-sided Pr > |Z|**	<.0001

Sample Size = 210

Tukey Style Multiple Comparisons of Proportions

Compare	Diff	SE	q	q(.001)	Conclude
3 vs 5	70.1	1.97	35.58	5.484	R
3 vs 4	47.85	1.97	24.29	5.484	R
3 vs 2	15.16	1.97	7.69	5.484	R
3 vs 1	0	1.97	0	5.484	A
1 vs 5	70.1	1.97	35.58	5.484	R
1 vs 4	47.85	1.97	24.29	5.484	R
1 vs 2	15.16	1.97	7.69	5.484	R
2 vs 5	54.94	1.97	27.89	5.484	R
2 vs 4	32.7	1.97	16.6	5.484	R
4 vs 5	22.24	1.97	11.29	5.484	R

Reference: Biostatistical Analysis, Fourth Edition, Jerrold Zar, 1999, p564.

Figure 6b- % Larvae dead by Group;

Chi-Square Analysis for a 2x5 Figure

The FREQ Procedure

Frequency<br>Col Pct	LARVAE_DEAD	GROUP					
		1=Control_with	2=Control_without	3=CNP	4=AgCNP	5=AgNO3	Total
	Yes	12<br>5.71	18<br>8.57	15<br>7.14	138<br>65.71	178<br>84.76	361
	No	198<br>94.29	192<br>91.43	195<br>92.86	72<br>34.29	32<br>15.24	689
	Total	210	210	210	210	210	1050

Figure of LARVAE_DEAD by GROUP

Statistics for Figure of LARVAE_DEAD by GROUP

Statistic	DF	Value	Prob
Chi-Square	4	535.2138	<.0001
Likelihood Ratio Chi-Square	4	579.2057	<.0001
Mantel-Haenszel Chi-Square	1	430.8201	<.0001
Phi Coefficient		0.7140	
Contingency Coefficient		0.5811	
Cramer's V		0.7140	

Sample Size = 1050

Larvae Dead with SE by Group

The FREQ Procedure
GROUP=1=Control_with

LARVAE_DEAD	Frequency	Percent	Cumulative Frequency	Cumulative Percent
**Yes**	12	5.71	12	5.71
**No**	198	94.29	210	100.00

Binomial Proportion	
**LARVAE_DEAD = Yes**	
**Proportion**	0.0571
**ASE**	0.0160
**95% Lower Conf Limit**	0.0257
**95% Upper Conf Limit**	0.0885
**Exact Conf Limits**	
**95% Lower Conf Limit**	0.0299
**95% Upper Conf Limit**	0.0977

Test of H0: Proportion = 0.5	
**ASE under H0**	0.0345
**Z**	-12.8352
**One-sided Pr < Z**	<.0001
**Two-sided Pr > |Z|**	<.0001

Sample Size = 210

Larvae Dead with SE by Group

The FREQ Procedure
GROUP=2=Control_without

LARVAE_DEAD	Frequency	Percent	Cumulative Frequency	Cumulative Percent
Yes	18	8.57	18	8.57
No	192	91.43	210	100.00

Binomial Proportion	
LARVAE_DEAD = Yes	
Proportion	0.0857
ASE	0.0193
95% Lower Conf Limit	0.0479
95% Upper Conf Limit	0.1236
Exact Conf Limits	
95% Lower Conf Limit	0.0516
95% Upper Conf Limit	0.1321

Test of H0: Proportion = 0.5	
ASE under H0	0.0345
Z	-12.0071
One-sided Pr < Z	<.0001
Two-sided Pr > |Z|	<.0001

Sample Size = 210

Larvae Dead with SE by Group

The FREQ Procedure
GROUP=3=CNP

LARVAE_DEAD	Frequency	Percent	Cumulative Frequency	Cumulative Percent
Yes	15	7.14	15	7.14
No	195	92.86	210	100.00

Binomial Proportion	
LARVAE_DEAD = Yes	
Proportion	0.0714
ASE	0.0178
95% Lower Conf Limit	0.0366
95% Upper Conf Limit	0.1063
Exact Conf Limits	
95% Lower Conf Limit	0.0405
95% Upper Conf Limit	0.1151

Test of H0: Proportion = 0.5	
ASE under H0	0.0345
Z	-12.4212
One-sided Pr < Z	<.0001
Two-sided Pr > |Z|	<.0001

Sample Size = 210

Larvae Dead with SE by Group

The FREQ Procedure
GROUP=4=AgCNP

LARVAE_DEAD	Frequency	Percent	Cumulative Frequency	Cumulative Percent
**Yes**	138	65.71	138	65.71
**No**	72	34.29	210	100.00

Binomial Proportion	
**LARVAE_DEAD = Yes**	
**Proportion**	0.6571
**ASE**	0.0328
**95% Lower Conf Limit**	0.5929
**95% Upper Conf Limit**	0.7213
**Exact Conf Limits**	
**95% Lower Conf Limit**	0.5887
**95% Upper Conf Limit**	0.7211

Test of H0: Proportion = 0.5	
**ASE under H0**	0.0345
**Z**	4.5544
**One-sided Pr > Z**	<.0001
**Two-sided Pr > |Z|**	<.0001

Sample Size = 210

Larvae Dead with SE by Group

The FREQ Procedure
GROUP=5=AgNO3

LARVAE_DEAD	Frequency	Percent	Cumulative Frequency	Cumulative Percent
**Yes**	178	84.76	178	84.76
**No**	32	15.24	210	100.00

Binomial Proportion	
**LARVAE_DEAD = Yes**	
**Proportion**	0.8476
**ASE**	0.0248
**95% Lower Conf Limit**	0.7990
**95% Upper Conf Limit**	0.8962
**Exact Conf Limits**	
**95% Lower Conf Limit**	0.7917
**95% Upper Conf Limit**	0.8934

Test of H0: Proportion = 0.5	
**ASE under H0**	0.0345
**Z**	10.0750
**One-sided Pr > Z**	<.0001
**Two-sided Pr > |Z|**	<.0001

Sample Size = 210

Tukey Style Multiple Comparisons of Proportions

Compare	Diff	SE	q	q(.001)	Conclude
5 vs 1	52.82	1.97	26.81	5.484	R
5 vs 3	51.18	1.97	25.98	5.484	R
5 vs 2	49.68	1.97	25.22	5.484	R
5 vs 4	12.78	1.97	6.49	5.484	R
4 vs 1	40.04	1.97	20.32	5.484	R
4 vs 3	38.4	1.97	19.49	5.484	R
4 vs 2	36.9	1.97	18.73	5.484	R
2 vs 1	3.14	1.97	1.59	5.484	A
2 vs 3	1.5	1.97	0.76	5.484	A
3 vs 1	1.64	1.97	0.83	5.484	A

Reference: Biostatistical Analysis, Fourth Edition, Jerrold Zar, 1999, p564.

Figure 6c- % Adults Emerged by Group;

Chi-Square Analysis for a 2x5 Figure

The FREQ Procedure

Frequency<br>Col Pct	EMERGED	GROUP: 1=Control_with	2=Control_without	3=CNP	4=AgCNP	5=AgNO3	Total
	Yes	161	150	141	34	0	486
		89.44	83.33	78.33	18.89	0.00	
	No	19	30	39	146	180	414
		10.56	16.67	21.67	81.11	100.00	
	Total	180	180	180	180	180	900

Table: Figure of EMERGED by GROUP

Statistics for Figure of EMERGED by GROUP

Statistic	DF	Value	Prob
Chi-Square	4	496.9315	<.0001
Likelihood Ratio Chi-Square	4	595.7180	<.0001
Mantel-Haenszel Chi-Square	1	428.5893	<.0001
Phi Coefficient		0.7431	
Contingency Coefficient		0.5964	
Cramer's V		0.7431	

Sample Size = 900

Proportion Emerged with SE by Group

The FREQ Procedure
GROUP=1=Control_with

EMERGED	Frequency	Percent	Cumulative Frequency	Cumulative Percent
**Yes**	161	89.44	161	89.44
**No**	19	10.56	180	100.00

Binomial Proportion	
**EMERGED = Yes**	
**Proportion**	0.8944
**ASE**	0.0229
**95% Lower Conf Limit**	0.8496
**95% Upper Conf Limit**	0.9393
**Exact Conf Limits**	
**95% Lower Conf Limit**	0.8401
**95% Upper Conf Limit**	0.9352

Test of H0: Proportion = 0.5	
**ASE under H0**	0.0373
**Z**	10.5841
**One-sided Pr > Z**	<.0001
**Two-sided Pr > |Z|**	<.0001

Sample Size = 180

Proportion Emerged with SE by Group

The FREQ Procedure
GROUP=2=Control_without

EMERGED	Frequency	Percent	Cumulative Frequency	Cumulative Percent
Yes	150	83.33	150	83.33
No	30	16.67	180	100.00

Binomial Proportion	
EMERGED = Yes	
Proportion	0.8333
ASE	0.0278
95% Lower Conf Limit	0.7789
95% Upper Conf Limit	0.8878
Exact Conf Limits	
95% Lower Conf Limit	0.7707
95% Upper Conf Limit	0.8846

Test of H0: Proportion = 0.5	
ASE under H0	0.0373
Z	8.9443
One-sided Pr > Z	<.0001
Two-sided Pr > |Z|	<.0001

Sample Size = 180

Proportion Emerged with SE by Group

The FREQ Procedure
GROUP=3=CNP

EMERGED	Frequency	Percent	Cumulative Frequency	Cumulative Percent
**Yes**	141	78.33	141	78.33
**No**	39	21.67	180	100.00

Binomial Proportion	
**EMERGED = Yes**	
**Proportion**	0.7833
**ASE**	0.0307
**95% Lower Conf Limit**	0.7231
**95% Upper Conf Limit**	0.8435
**Exact Conf Limits**	
**95% Lower Conf Limit**	0.7159
**95% Upper Conf Limit**	0.8412

Test of H0: Proportion = 0.5	
**ASE under H0**	0.0373
**Z**	7.6026
**One-sided Pr > Z**	<.0001
**Two-sided Pr > |Z|**	<.0001

Sample Size = 180

Proportion Emerged with SE by Group

The FREQ Procedure
GROUP=4=AgCNP

EMERGED	Frequency	Percent	Cumulative Frequency	Cumulative Percent
Yes	34	18.89	34	18.89
No	146	81.11	180	100.00

Binomial Proportion	
EMERGED = Yes	
Proportion	0.1889
ASE	0.0292
95% Lower Conf Limit	0.1317
95% Upper Conf Limit	0.2461
Exact Conf Limits	
95% Lower Conf Limit	0.1345
95% Upper Conf Limit	0.2538

Test of H0: Proportion = 0.5	
ASE under H0	0.0373
Z	-8.3480
One-sided Pr < Z	<.0001
Two-sided Pr > |Z|	<.0001

Sample Size = 180

Proportion Emerged with SE by Group

The FREQ Procedure
GROUP=5=AgNO3

EMERGED	Frequency	Percent	Cumulative Frequency	Cumulative Percent
**No**	180	100.00	180	100.00

Binomial Proportion	
**EMERGED = No**	
**Proportion**	1.0000
**ASE**	0.0000
**95% Lower Conf Limit**	1.0000
**95% Upper Conf Limit**	1.0000
**Exact Conf Limits**	
**95% Lower Conf Limit**	0.9797
**95% Upper Conf Limit**	1.0000

Test of H0: Proportion = 0.5	
**ASE under H0**	0.0373
**Z**	13.4164
**One-sided Pr > Z**	<.0001
**Two-sided Pr > |Z|**	<.0001

Sample Size = 180

Tukey Style Multiple Comparisons of Proportions

Compare	Diff	SE	q	q(.05)	Conclude
1 vs 5	67.94	2.13	31.9	3.585	R
1 vs 4	44.96	2.13	21.11	3.585	R
1 vs 3	8.69	2.13	4.08	3.585	R
1 vs 2	5.08	2.13	2.38	3.585	A
2 vs 5	62.87	2.13	29.52	3.585	R
2 vs 4	39.89	2.13	18.73	3.585	R
2 vs 3	3.61	2.13	1.7	3.585	A
3 vs 5	59.25	2.13	27.82	3.585	R
3 vs 4	36.27	2.13	17.03	3.585	R
4 vs 5	22.98	2.13	10.79	3.585	R

Tukey Style Multiple Comparisons of Proportions

Compare	Diff	SE	q	q(.01)	Conclude
1 vs 5	67.94	2.13	31.9	4.603	R
1 vs 4	44.96	2.13	21.11	4.603	R
1 vs 3	8.69	2.13	4.08	4.603	A
1 vs 2	5.08	2.13	2.38	4.603	A
2 vs 5	62.87	2.13	29.52	4.603	R
2 vs 4	39.89	2.13	18.73	4.603	R
2 vs 3	3.61	2.13	1.7	4.603	A
3 vs 5	59.25	2.13	27.82	4.603	R
3 vs 4	36.27	2.13	17.03	4.603	R
4 vs 5	22.98	2.13	10.79	4.603	R

Reference: Biostatistical Analysis, Fourth Edition, Jerrold Zar, 1999, p564.

Tukey Style Multiple Comparisons of Proportions

Compare	Diff	SE	q	q(.001)	Conclude
1 vs 5	67.94	2.13	31.9	5.484	R
1 vs 4	44.96	2.13	21.11	5.484	R
1 vs 3	8.69	2.13	4.08	5.484	A
1 vs 2	5.08	2.13	2.38	5.484	A
2 vs 5	62.87	2.13	29.52	5.484	R
2 vs 4	39.89	2.13	18.73	5.484	R
2 vs 3	3.61	2.13	1.7	5.484	A
3 vs 5	59.25	2.13	27.82	5.484	R
3 vs 4	36.27	2.13	17.03	5.484	R
4 vs 5	22.98	2.13	10.79	5.484	R

Reference: Biostatistical Analysis, Fourth Edition, Jerrold Zar, 1999, p564.

Figure 6c- % Larvae dead by Group;

Chi-Square Analysis for a 2x5 Figure

The FREQ Procedure

Frequency<br>Col Pct	LARVAE_DEAD	Figure of LARVAE_DEAD by GROUP					
		GROUP					
		1=Control_with	2=Control_without	3=CNP	4=AgCNP	5=AgNO3	Total
	Yes	11	23	31	104	113	282

	6.11	12.78	17.22	57.78	62.78	
**No**	169	157	149	76	67	618
	93.89	87.22	82.78	42.22	37.22	
**Total**	180	180	180	180	180	900

Statistics for Figure of LARVAE_DEAD by GROUP

Statistic	DF	Value	Prob
**Chi-Square**	4	239.9091	<.0001
**Likelihood Ratio Chi-Square**	4	250.5727	<.0001
**Mantel-Haenszel Chi-Square**	1	209.4989	<.0001
**Phi Coefficient**		0.5163	
**Contingency Coefficient**		0.4588	
**Cramer's V**		0.5163	

Sample Size = 900

Larvae Dead with SE by Group

The FREQ Procedure
GROUP=1=Control_with

LARVAE_DEAD	Frequency	Percent	Cumulative Frequency	Cumulative Percent
**Yes**	11	6.11	11	6.11
**No**	169	93.89	180	100.00

Binomial Proportion	
LARVAE_DEAD = Yes	
**Proportion**	0.0611
**ASE**	0.0179
**95% Lower Conf Limit**	0.0261
**95% Upper Conf Limit**	0.0961
**Exact Conf Limits**	
**95% Lower Conf Limit**	0.0309
**95% Upper Conf Limit**	0.1067

Test of H0: Proportion = 0.5	
**ASE under H0**	0.0373
**Z**	-11.7766
**One-sided Pr < Z**	<.0001
**Two-sided Pr > |Z|**	<.0001

Sample Size = 180

Larvae Dead with SE by Group

The FREQ Procedure
GROUP=2=Control_without

LARVAE_DEAD	Frequency	Percent	Cumulative Frequency	Cumulative Percent
Yes	23	12.78	23	12.78
No	157	87.22	180	100.00

Binomial Proportion	
LARVAE_DEAD = Yes	
Proportion	0.1278
ASE	0.0249
95% Lower Conf Limit	0.0790
95% Upper Conf Limit	0.1765
Exact Conf Limits	
95% Lower Conf Limit	0.0828
95% Upper Conf Limit	0.1855

Test of H0: Proportion = 0.5	
ASE under H0	0.0373
Z	-9.9878
One-sided Pr < Z	<.0001
Two-sided Pr > |Z|	<.0001

Sample Size = 180

Larvae Dead with SE by Group

The FREQ Procedure
GROUP=3=CNP

LARVAE_DEAD	Frequency	Percent	Cumulative Frequency	Cumulative Percent
Yes	31	17.22	31	17.22
No	149	82.78	180	100.00

Binomial Proportion	
LARVAE_DEAD = Yes	
Proportion	0.1722
ASE	0.0281
95% Lower Conf Limit	0.1171
95% Upper Conf Limit	0.2274
Exact Conf Limits	
95% Lower Conf Limit	0.1201
95% Upper Conf Limit	0.2355

Test of H0: Proportion = 0.5	
ASE under H0	0.0373
Z	-8.7952
One-sided Pr < Z	<.0001
Two-sided Pr > |Z|	<.0001

Sample Size = 180

Larvae Dead with SE by Group

The FREQ Procedure
GROUP=4=AgCNP

LARVAE_DEAD	Frequency	Percent	Cumulative Frequency	Cumulative Percent
**Yes**	104	57.78	104	57.78
**No**	76	42.22	180	100.00

Binomial Proportion	
**LARVAE_DEAD = Yes**	
**Proportion**	0.5778
**ASE**	0.0368
**95% Lower Conf Limit**	0.5056
**95% Upper Conf Limit**	0.6499
**Exact Conf Limits**	
**95% Lower Conf Limit**	0.5021
**95% Upper Conf Limit**	0.6509

Test of H0: Proportion = 0.5	
**ASE under H0**	0.0373
**Z**	2.0870
**One-sided Pr > Z**	0.0184
**Two-sided Pr > |Z|**	0.0369

Sample Size = 180

Larvae Dead with SE by Group

The FREQ Procedure
GROUP=5=AgNO3

LARVAE_DEAD	Frequency	Percent	Cumulative Frequency	Cumulative Percent
Yes	113	62.78	113	62.78
No	67	37.22	180	100.00

Binomial Proportion	
LARVAE_DEAD = Yes	
Proportion	0.6278
ASE	0.0360
95% Lower Conf Limit	0.5572
95% Upper Conf Limit	0.6984
Exact Conf Limits	
95% Lower Conf Limit	0.5527
95% Upper Conf Limit	0.6985

Test of H0: Proportion = 0.5	
ASE under H0	0.0373
Z	3.4286
One-sided Pr > Z	0.0003
Two-sided Pr > |Z|	0.0006

Sample Size = 180

Tukey Style Multiple Comparisons of Proportions

Compare	Diff	SE	q	q(.05)	Conclude
5 vs 1	37.77	2.13	17.73	3.585	R
5 vs 2	31.25	2.13	14.67	3.585	R
5 vs 3	27.71	2.13	13.01	3.585	R
5 vs 4	2.91	2.13	1.37	3.585	A
4 vs 1	34.86	2.13	16.37	3.585	R
4 vs 2	28.34	2.13	13.3	3.585	R
4 vs 3	24.8	2.13	11.64	3.585	R
3 vs 1	10.06	2.13	4.72	3.585	R
3 vs 2	3.54	2.13	1.66	3.585	A
2 vs 1	6.52	2.13	3.06	3.585	A

Reference: Biostatistical Analysis, Fourth Edition, Jerrold Zar, 1999, p564.

Tukey Style Multiple Comparisons of Proportions

Compare	Diff	SE	q	q(.01)	Conclude
5 vs 1	37.77	2.13	17.73	4.603	R
5 vs 2	31.25	2.13	14.67	4.603	R
5 vs 3	27.71	2.13	13.01	4.603	R
5 vs 4	2.91	2.13	1.37	4.603	A
4 vs 1	34.86	2.13	16.37	4.603	R
4 vs 2	28.34	2.13	13.3	4.603	R
4 vs 3	24.8	2.13	11.64	4.603	R
3 vs 1	10.06	2.13	4.72	4.603	R
3 vs 2	3.54	2.13	1.66	4.603	A
2 vs 1	6.52	2.13	3.06	4.603	A

Reference: Biostatistical Analysis, Fourth Edition, Jerrold Zar, 1999, p564.

Tukey Style Multiple Comparisons of Proportions

Compare	Diff	SE	q	q(.001)	Conclude
5 vs 1	37.77	2.13	17.73	5.484	R
5 vs 2	31.25	2.13	14.67	5.484	R
5 vs 3	27.71	2.13	13.01	5.484	R
5 vs 4	2.91	2.13	1.37	5.484	A
4 vs 1	34.86	2.13	16.37	5.484	R
4 vs 2	28.34	2.13	13.3	5.484	R

```
   4 vs 3    24.8      2.13      11.64     5.484     R
   3 vs 1    10.06     2.13      4.72      5.484     A
   3 vs 2    3.54      2.13      1.66      5.484     A
   2 vs 1    6.52      2.13      3.06      5.484     A

 Reference: Biostatistical Analysis, Fourth Edition, Jerrold Zar, 1999, p564.
```
