# Supplementary material for: Exposure to nanoceria impacts larval survival, life history traits and fecundity of Aedes aegypti
Source: PLoS Negl Trop Dis. 2020 Sep 25;14(9):e0008654. doi: 10.1371/journal.pntd.0008654 (PMC7540862; doi:10.1371/journal.pntd.0008654)
Supplement: S3 File — (PDF) [file pntd.0008654.s007.pdf]

# Figure 7a-Number of Females by Group;

The SAS System

The NPAR1WAY Procedure

**Wilcoxon Scores (Rank Sums) for Variable FEMALES Classified by Variable GROUP**

| GROUP | N | Sum of Scores | Expected Under H0 | Std Dev Under H0 | Mean Score |
|---|---|---|---|---|---|
| **1=Control** | 6 | 67.50 | 57.0 | 10.583005 | 11.250000 |
| **2=CNP** | 6 | 53.50 | 57.0 | 10.583005 | 8.916667 |
| **3=AgCNP** | 6 | 50.00 | 57.0 | 10.583005 | 8.333333 |

**Average scores were used for ties.**

**Kruskal-Wallis Test**

| Chi-Square | DF | Pr > ChiSq |
|---|---|---|
| 1.0208 | 2 | 0.6002 |

GROUP=1=Control

**Analysis Variable : FEMALES**

| N | Minimum | Maximum | Mean | Median | Std Dev | Std Error | Lower 95% CL for Mean | Upper 95% CL for Mean |
|---|---|---|---|---|---|---|---|---|
| 6 | 5.0000000 | 13.0000000 | 9.6666667 | 10.0000000 | 2.6583203 | 1.0852547 | 6.8769306 | 12.4564027 |

GROUP=2=CNP

**Analysis Variable : FEMALES**

| N | Minimum | Maximum | Mean | Median | Std Dev | Std Error | Lower 95% CL for Mean | Upper 95% CL for Mean |
|---|---|---|---|---|---|---|---|---|
| 6 | 5.0000000 | 13.0000000 | 8.5000000 | 8.0000000 | 2.8106939 | 1.1474610 | 5.5503577 | 11.4496423 |

GROUP=3=AgCNP

**Analysis Variable : FEMALES**

| N | Minimum | Maximum | Mean | Median | Std Dev | Std Error | Lower 95% CL for Mean | Upper 95% CL for Mean |
|---|---|---|---|---|---|---|---|---|
| 6 | 1.0000000 | 13.0000000 | 7.5000000 | 8.0000000 | 4.8062459 | 1.9621417 | 2.4561542 | 12.5438458 |

## Figure 7a-Egg weight by Group;

The SAS System

The NPAR1WAY Procedure

**Wilcoxon Scores (Rank Sums) for Variable EGGS Classified by Variable GROUP**

| GROUP | N | Sum of Scores | Expected Under H0 | Std Dev Under H0 | Mean Score |
|---|---|---|---|---|---|
| **1=Control** | 6 | 52.50 | 57.0 | 10.643971 | 8.750 |
| **2=CNP** | 6 | 76.50 | 57.0 | 10.643971 | 12.750 |
| **3=AgCNP** | 6 | 42.00 | 57.0 | 10.643971 | 7.000 |

**Average scores were used for ties.**

**Kruskal-Wallis Test**

| Chi-Square | DF | Pr > ChiSq |
|---|---|---|
| 3.6807 | 2 | 0.1588 |

GROUP=1=Control

**Analysis Variable : EGGS**

| N | Minimum | Maximum | Mean | Median | Std Dev | Std Error | Lower 95% CL for Mean | Upper 95% CL for Mean |
|---|---|---|---|---|---|---|---|---|
| 6 | 0 | 4.2000000 | 1.4166667 | 0.8500000 | 1.6690317 | 0.6813793 | -0.3348746 | 3.1682080 |

GROUP=2=CNP

**Analysis Variable : EGGS**

| N | Minimum | Maximum | Mean | Median | Std Dev | Std Error | Lower 95% CL for Mean | Upper 95% CL for Mean |
|---|---|---|---|---|---|---|---|---|
| 6 | 1.6000000 | 2.6000000 | 1.9666667 | 1.9500000 | 0.3502380 | 0.1429841 | 1.5991144 | 2.3342189 |

GROUP=3=AgCNP

**Analysis Variable : EGGS**

| N | Minimum | Maximum | Mean | Median | Std Dev | Std Error | Lower 95% CL for Mean | Upper 95% CL for Mean |
|---|---|---|---|---|---|---|---|---|
| 6 | 0 | 2.1000000 | 0.8666667 | 0.6500000 | 0.7916228 | 0.3231787 | 0.0359095 | 1.6974239 |

## Figure 7b-Number of Females by Group;

Used the normality test for small samples based on calculating the z-score from Skewness as described in: 2012_Normality Tests for Statistical Analysis: A Guide for Non-Statisticians. All sample means failed to reject the null hypothesis that the data come from a normal distribution. Thus, we used ANOVA to evaluate mean differences.

The GLM Procedure

Dependent Variable: FEMALES

| Source | DF | Sum of Squares | Mean Square | F Value | Pr > F |
|---|---|---|---|---|---|
| **Model** | 3 | 380.9789683 | 126.9929894 | 39.46 | <.0001 |
| **Error** | 31 | 99.7638889 | 3.2181900 | | |
| **Corrected Total** | 34 | 480.7428571 | | | |

The SAS System

The MEANS Procedure
GROUP=1=Control_with

**Analysis Variable : FEMALES**

| N | Minimum | Maximum | Mean | Median | Std Dev | Std Error | Lower 95% CL for Mean | Upper 95% CL for Mean |
|---|---|---|---|---|---|---|---|---|
| 9 | 5.0000000 | 11.0000000 | 8.8888889 | 10.0000000 | 1.9002924 | 0.6334308 | 7.4281949 | 10.3495829 |

GROUP=2=Control_without

**Analysis Variable : FEMALES**

| N | Minimum | Maximum | Mean | Median | Std Dev | Std Error | Lower 95% CL for Mean | Upper 95% CL for Mean |
|---|---|---|---|---|---|---|---|---|
| 9 | 7.0000000 | 13.0000000 | 10.3333333 | 10.0000000 | 1.8708287 | 0.6236096 | 8.8952871 | 11.7713796 |

GROUP=3=CNP

**Analysis Variable : FEMALES**

| N | Minimum | Maximum | Mean | Median | Std Dev | Std Error | Lower 95% CL for Mean | Upper 95% CL for Mean |
|---|---|---|---|---|---|---|---|---|
| 9 | 9.0000000 | 13.0000000 | 10.3333333 | 10.0000000 | 1.4142136 | 0.4714045 | 9.2462726 | 11.4203941 |

GROUP=4=AgCNP

**Analysis Variable : FEMALES**

| N | Minimum | Maximum | Mean | Median | Std Dev | Std Error | Lower 95% CL for Mean | Upper 95% CL for Mean |
|---|---|---|---|---|---|---|---|---|
| 8 | 0 | 6.0000000 | 2.1250000 | 2.0000000 | 1.9594095 | 0.6927559 | 0.4868926 | 3.7631074 |

The GLM Procedure
Least Squares Means
Adjustment for Multiple Comparisons: Tukey-Kramer

| GROUP | FEMALES LSMEAN | LSMEAN Number |
|---|---|---|
| 1=Control_with | 8.8888889 | 1 |
| 2=Control_without | 10.3333333 | 2 |
| 3=CNP | 10.3333333 | 3 |
| 4=AgCNP | 2.1250000 | 4 |

**Least Squares Means for effect GROUP**
**Pr > |t| for H0: LSMean(i)=LSMean(j)**
**Dependent Variable: FEMALES**

| i/j | 1 | 2 | 3 | 4 |
|---|---|---|---|---|
| 1 | | 0.3368 | 0.3368 | <.0001 |
| 2 | 0.3368 | | 1.0000 | <.0001 |
| 3 | 0.3368 | 1.0000 | | <.0001 |
| 4 | <.0001 | <.0001 | <.0001 | |

| GROUP | FEMALES LSMEAN | 95% Confidence Limits | |
|---|---|---|---|
| 1=Control_with | 8.888889 | 7.669306 | 10.108471 |
| 2=Control_without | 10.333333 | 9.113751 | 11.552916 |
| 3=CNP | 10.333333 | 9.113751 | 11.552916 |
| 4=AgCNP | 2.125000 | 0.831437 | 3.418563 |

**Figure 7b-Egg weight by Group;**

**AgCNP rejected the normality assumption by the skewness test described above. Thus, we used Kruskal-Wallis which established that at least one median is different.**

**The NPAR1WAY Procedure**

**Analysis of Variance for Variable EGGS**
**Classified by Variable GROUP**

| GROUP | N | Mean |
|---|---|---|
| 1=Control_with | 9 | 2.711111 |
| 2=Control_without | 9 | 2.866667 |
| 3=CNP | 9 | 1.133333 |
| 4=AgCNP | 6 | 0.633333 |

**Kruskal-Wallis Test**

| Chi-Square | DF | Pr > ChiSq |
|---|---|---|
| 11.5990 | 3 | 0.0089 |

GROUP=1=Control_with

**Analysis Variable : EGGS**

| N | Minimum | Maximum | Mean | Median | Std Dev | Std Error | Lower 95% CL for Mean | Upper 95% CL for Mean |
|---|---|---|---|---|---|---|---|---|
| 9 | 0 | 5.4000000 | 2.7111111 | 2.6000000 | 2.2430138 | 0.7476713 | 0.9869780 | 4.4352442 |

GROUP=2=Control_without

**Analysis Variable : EGGS**

| N | Minimum | Maximum | Mean | Median | Std Dev | Std Error | Lower 95% CL for Mean | Upper 95% CL for Mean |
|---|---|---|---|---|---|---|---|---|
| 9 | 1.1000000 | 4.3000000 | 2.8666667 | 3.0000000 | 1.0185774 | 0.3395258 | 2.0837187 | 3.6496146 |

GROUP=3=CNP

**Analysis Variable : EGGS**

| N | Minimum | Maximum | Mean | Median | Std Dev | Std Error | Lower 95% CL for Mean | Upper 95% CL for Mean |
|---|---|---|---|---|---|---|---|---|
| 9 | 0.2000000 | 1.8000000 | 1.1333333 | 1.4000000 | 0.5809475 | 0.1936492 | 0.6867776 | 1.5798891 |

GROUP=4=AgCNP

**Analysis Variable : EGGS**

| N | Minimum | Maximum | Mean | Median | Std Dev | Std Error | Lower 95% CL for Mean | Upper 95% CL for Mean |
|---|---|---|---|---|---|---|---|---|
| 6 | 0 | 2.8000000 | 0.6333333 | 0.2500000 | 1.0782702 | 0.4402020 | -0.4982419 | 1.7649085 |

```
Group sample sizes not equal, or some ranks tied. Performed Dunn's test, alpha=0.05

Comparison group = GROUP_NEW

Compare   Diff      SE        q         q(0.05)    Conclude
------------------------------------------------------------
2 vs  4   15.78     5.09      3.1       2.638    Reject
2 vs  3   10.06     4.55      2.21      2.638    Do not reject
2 vs  1   Do not reject (within non-sig. comparison)
1 vs  4   12.11     5.09      2.38      2.638    Do not reject
1 vs  3   Do not reject (within non-sig. comparison)
3 vs  4   Do not reject (within non-sig. comparison)

Note: "Do not reject (within non-sig. comparison)" indicates that any comparison
within the range of a non-significant comparison must also be non-significant.
```

Reference: Biostatistical Analysis, 4th Edition, J. Zar, 2010.
