# Supplementary material for: Exposure to nanoceria impacts larval survival, life history traits and fecundity of Aedes aegypti
Source: PLoS Negl Trop Dis. 2020 Sep 25;14(9):e0008654. doi: 10.1371/journal.pntd.0008654 (PMC7540862; doi:10.1371/journal.pntd.0008654)
Supplement: S4 File — (PDF) [file pntd.0008654.s008.pdf]

Figure 8a-% Eggs Hatched;

The FREQ Procedure

Table of EGGS_HATCHED_YES by GROUP

Frequency / Col Pct	GROUP			
EGGS_HATCHED_YES	1=Contro	2=CNP	3=AgCNP	Total
Yes	275<br>77.25	431<br>71.83	237<br>66.02	943
No	81<br>22.75	169<br>28.17	122<br>33.98	372
Total	356	600	359	1315

Statistics for Table of EGGS_HATCHED_YES by GROUP

Statistic	DF	Value	Prob
Chi-Square	2	11.1211	0.0038
Likelihood Ratio Chi-Square	2	11.1565	0.0038
Mantel-Haenszel Chi-Square	1	11.1062	0.0009
Phi Coefficient		0.0920	
Contingency Coefficient		0.0916	
Cramer's V		0.0920	

Sample Size = 1315

Proportion Eggs Hatched with SE by Group

The FREQ Procedure
GROUP=1=Contro

EGGS_HATCHED_YES	Frequency	Percent	Cumulative Frequency	Cumulative Percent
**Yes**	275	77.25	275	77.25
**No**	81	22.75	356	100.00

Binomial Proportion	
**EGGS_HATCHED_YES = Yes**	
**Proportion**	0.7725
**ASE**	0.0222
**95% Lower Conf Limit**	0.7289
**95% Upper Conf Limit**	0.8160
**Exact Conf Limits**	
**95% Lower Conf Limit**	0.7253
**95% Upper Conf Limit**	0.8150

Test of H0: Proportion = 0.5	
**ASE under H0**	0.0265
**Z**	10.2820
**One-sided Pr > Z**	<.0001
**Two-sided Pr > |Z|**	<.0001

The FREQ Procedure
GROUP=2=CNP

EGGS_HATCHED_YES	Frequency	Percent	Cumulative Frequency	Cumulative Percent
**Yes**	431	71.83	431	71.83
**No**	169	28.17	600	100.00

Binomial Proportion	
**EGGS_HATCHED_YES = Yes**	
**Proportion**	0.7183
**ASE**	0.0184
**95% Lower Conf Limit**	0.6823
**95% Upper Conf Limit**	0.7543
**Exact Conf Limits**	
**95% Lower Conf Limit**	0.6805
**95% Upper Conf Limit**	0.7540

Test of H0: Proportion = 0.5	
**ASE under H0**	0.0204
**Z**	10.6961
**One-sided Pr > Z**	<.0001
**Two-sided Pr > |Z|**	<.0001

Sample Size = 600

Proportion Eggs Hatched with SE by Group

The FREQ Procedure
GROUP=3=AgCNP

EGGS_HATCHED_YES	Frequency	Percent	Cumulative Frequency	Cumulative Percent
**Yes**	237	66.02	237	66.02
**No**	122	33.98	359	100.00

Binomial Proportion

EGGS_HATCHED_YES = Yes	
**Proportion**	0.6602
**ASE**	0.0250
**95% Lower Conf Limit**	0.6112
**95% Upper Conf Limit**	0.7092
**Exact Conf Limits**	
**95% Lower Conf Limit**	0.6086
**95% Upper Conf Limit**	0.7091

Test of H0: Proportion = 0.5	
**ASE under H0**	0.0264
**Z**	6.0695
**One-sided Pr > Z**	<.0001
**Two-sided Pr > |Z|**	<.0001

Sample Size = 359

Tukey Style Multiple Comparisons of Proportions

Compare	Diff	SE	q	q(.05)	Conclude
1 vs 3	7.15	1.51	4.73	3.314	R
1 vs 2	3.54	1.35	2.62	3.314	A
2 vs 3	3.61	1.35	2.67	3.314	A

Reference: Biostatistical Analysis, Fourth Edition, Jerrold Zar, 1999, p564.

Tukey Style Multiple Comparisons of Proportions

Compare	Diff	SE	q	q(.01)	Conclude
1 vs 3	7.15	1.51	4.73	4.12	R
1 vs 2	3.54	1.35	2.62	4.12	A
2 vs 3	3.61	1.35	2.67	4.12	A

Reference: Biostatistical Analysis, Fourth Edition, Jerrold Zar, 1999, p564.

Figure 8a-% Adults Emerged;

The FREQ Procedure

Table of ADULTS_EMERGED_YES by GROUP

Frequency<br>Col Pct	ADULTS_EMERGED_YES	GROUP			
		1=Control	2=CNP	3=AgCNP	Total
	Yes	205	337	169	711
		57.58	56.17	47.08	
	No	151	263	190	604
		42.42	43.83	52.92	
	Total	356	600	359	1315

Statistics for Table of ADULTS_EMERGED_YES by GROUP

Statistic	DF	Value	Prob
Chi-Square	2	9.9052	0.0071
Likelihood Ratio Chi-Square	2	9.8857	0.0071
Mantel-Haenszel Chi-Square	1	7.9652	0.0048
Phi Coefficient		0.0868	
Contingency Coefficient		0.0865	
Cramer's V		0.0868	

Proportion Adults Emerged with SE by Group

The FREQ Procedure

GROUP=1=Control

ADULTS_EMERGED_YES	Frequency	Percent	Cumulative Frequency	Cumulative Percent
**Yes**	205	57.58	205	57.58
**No**	151	42.42	356	100.00

Binomial Proportion	
**ADULTS_EMERGED_YES = Yes**	
**Proportion**	0.5758
**ASE**	0.0262
**95% Lower Conf Limit**	0.5245
**95% Upper Conf Limit**	0.6272
**Exact Conf Limits**	
**95% Lower Conf Limit**	0.5226
**95% Upper Conf Limit**	0.6278

Test of H0: Proportion = 0.5	
**ASE under H0**	0.0265
**Z**	2.8620
**One-sided Pr > Z**	0.0021
**Two-sided Pr > |Z|**	0.0042

Sample Size = 356

Proportion Adults Emerged with SE by Group

The FREQ Procedure
GROUP=2=CNP

ADULTS_EMERGED_YES	Frequency	Percent	Cumulative Frequency	Cumulative Percent
**Yes**	337	56.17	337	56.17
**No**	263	43.83	600	100.00

Binomial Proportion	
**ADULTS_EMERGED_YES = Yes**	
**Proportion**	0.5617
**ASE**	0.0203
**95% Lower Conf Limit**	0.5220
**95% Upper Conf Limit**	0.6014
**Exact Conf Limits**	
**95% Lower Conf Limit**	0.5209
**95% Upper Conf Limit**	0.6018

Test of H0: Proportion = 0.5	
**ASE under H0**	0.0204
**Z**	3.0210
**One-sided Pr > Z**	0.0013
**Two-sided Pr > |Z|**	0.0025

Sample Size = 600

Proportion Adults Emerged with SE by Group

The FREQ Procedure
GROUP=3=AgCNP

ADULTS_EMERGED_YES	Frequency	Percent	Cumulative Frequency	Cumulative Percent
**Yes**	169	47.08	169	47.08
**No**	190	52.92	359	100.00

Binomial Proportion	
**ADULTS_EMERGED_YES = Yes**	
**Proportion**	0.4708
**ASE**	0.0263
**95% Lower Conf Limit**	0.4191
**95% Upper Conf Limit**	0.5224
**Exact Conf Limits**	
**95% Lower Conf Limit**	0.4182
**95% Upper Conf Limit**	0.5238

Test of H0: Proportion = 0.5	
**ASE under H0**	0.0264
**Z**	-1.1083
**One-sided Pr < Z**	0.1339
**Two-sided Pr > |Z|**	0.2677

Sample Size = 359

Tukey Style Multiple Comparisons of Proportions

Compare	Diff	SE	q	q(.05)	Conclude
1 vs 3	6.02	1.51	3.99	3.314	R
1 vs 2	0.81	1.35	0.6	3.314	A
2 vs 3	5.21	1.35	3.86	3.314	R

Reference: Biostatistical Analysis, Fourth Edition, Jerrold Zar, 1999, p564.

Tukey Style Multiple Comparisons of Proportions

Compare	Diff	SE	q	q(.01)	Conclude
1 vs 3	6.02	1.51	3.99	4.12	A
1 vs 2	0.81	1.35	0.6	4.12	A
2 vs 3	5.21	1.35	3.86	4.12	A

Reference: Biostatistical Analysis, Fourth Edition, Jerrold Zar, 1999, p564.

Figure 8a-% Larvae Pupae;

The FREQ Procedure

Table of LARVAE_PUPAE_YES by GROUP

Frequency / Col Pct	LARVAE_PUPAE_YES	GROUP: 1=Control	2=CNP	3=AgCNP	Total
	Yes	70	95	59	224
		19.66	15.83	16.43	
	No	286	505	300	1091
		80.34	84.17	83.57	
	Total	356	600	359	1315

Statistics for Table of LARVAE_PUPAE_YES by GROUP

Statistic	DF	Value	Prob
Chi-Square	2	2.4443	0.2946
Likelihood Ratio Chi-Square	2	2.3911	0.3025
Mantel-Haenszel Chi-Square	1	1.3103	0.2523
Phi Coefficient		0.0431	
Contingency Coefficient		0.0431	
Cramer's V		0.0431	

Proportion Larvae_Pupae with SE by Group

The FREQ Procedure
GROUP=1=Control

LARVAE_PUPAE_YES	Frequency	Percent	Cumulative Frequency	Cumulative Percent
Yes	70	19.66	70	19.66
No	286	80.34	356	100.00

Binomial Proportion	
LARVAE_PUPAE_YES = Yes	
Proportion	0.1966
ASE	0.0211
95% Lower Conf Limit	0.1553
95% Upper Conf Limit	0.2379
Exact Conf Limits	
95% Lower Conf Limit	0.1566
95% Upper Conf Limit	0.2418

Test of H0: Proportion = 0.5	
ASE under H0	0.0265
Z	-11.4480
One-sided Pr < Z	<.0001
Two-sided Pr > |Z|	<.0001

Sample Size = 356

Proportion Larvae_Pupae with SE by Group

The FREQ Procedure
GROUP=2=CNP

LARVAE_PUPAE_YES	Frequency	Percent	Cumulative Frequency	Cumulative Percent
Yes	95	15.83	95	15.83
No	505	84.17	600	100.00

Binomial Proportion

LARVAE_PUPAE_YES = Yes	
Proportion	0.1583
ASE	0.0149
95% Lower Conf Limit	0.1291
95% Upper Conf Limit	0.1875
**Exact Conf Limits**	
95% Lower Conf Limit	0.1300
95% Upper Conf Limit	0.1900

Test of H0: Proportion = 0.5	
ASE under H0	0.0204
Z	-16.7382
One-sided Pr < Z	<.0001
Two-sided Pr > |Z|	<.0001

Sample Size = 600

Proportion Larvae_Pupae with SE by Group

The FREQ Procedure
GROUP=3=AgCNP

LARVAE_PUPAE_YES	Frequency	Percent	Cumulative Frequency	Cumulative Percent
Yes	59	16.43	59	16.43
No	300	83.57	359	100.00

Binomial Proportion	
LARVAE_PUPAE_YES = Yes	
Proportion	0.1643
ASE	0.0196
95% Lower Conf Limit	0.1260
95% Upper Conf Limit	0.2027
Exact Conf Limits	
95% Lower Conf Limit	0.1275
95% Upper Conf Limit	0.2068

Test of H0: Proportion = 0.5	
ASE under H0	0.0264
Z	-12.7195
One-sided Pr < Z	<.0001
Two-sided Pr > |Z|	<.0001

Sample Size = 359

Figure 8b-% Female;

The FREQ Procedure

Frequency
Col Pct

Table of FEMALE by GROUP

FEMALE	GROUP			
	1=Control	2=CNP	3=AgCNP	Total
Yes	73<br>35.61	166<br>49.26	73<br>43.20	312
No	132<br>64.39	171<br>50.74	96<br>56.80	399
Total	205	337	169	711

Statistics for Table of FEMALE by GROUP

Statistic	DF	Value	Prob
Chi-Square	2	9.6843	0.0079
Likelihood Ratio Chi-Square	2	9.7648	0.0076
Mantel-Haenszel Chi-Square	1	2.7191	0.0992
Phi Coefficient		0.1167	
Contingency Coefficient		0.1159	
Cramer's V		0.1167	

Sample Size = 711

Proportion Female with SE by Group

The FREQ Procedure
GROUP=1=Control

FEMALE	Frequency	Percent	Cumulative Frequency	Cumulative Percent
**Yes**	73	35.61	73	35.61
**No**	132	64.39	205	100.00

Binomial Proportion	
**FEMALE = Yes**	
**Proportion**	0.3561
**ASE**	0.0334
**95% Lower Conf Limit**	0.2905
**95% Upper Conf Limit**	0.4216
**Exact Conf Limits**	
**95% Lower Conf Limit**	0.2906
**95% Upper Conf Limit**	0.4258

Test of H0: Proportion = 0.5	
**ASE under H0**	0.0349
**Z**	-4.1207
**One-sided Pr < Z**	<.0001
**Two-sided Pr > |Z|**	<.0001

Sample Size = 205

Proportion Female with SE by Group

The FREQ Procedure
GROUP=2=CNP

FEMALE	Frequency	Percent	Cumulative Frequency	Cumulative Percent
**Yes**	166	49.26	166	49.26
**No**	171	50.74	337	100.00

Binomial Proportion	
**FEMALE = Yes**	
**Proportion**	0.4926
**ASE**	0.0272
**95% Lower Conf Limit**	0.4392
**95% Upper Conf Limit**	0.5460
**Exact Conf Limits**	
**95% Lower Conf Limit**	0.4380
**95% Upper Conf Limit**	0.5473

Test of H0: Proportion = 0.5	
**ASE under H0**	0.0272
**Z**	-0.2724
**One-sided Pr < Z**	0.3927
**Two-sided Pr > |Z|**	0.7853

Sample Size = 337

Proportion Female with SE by Group

The FREQ Procedure
GROUP=3=AgCNP

FEMALE	Frequency	Percent	Cumulative Frequency	Cumulative Percent
**Yes**	73	43.20	73	43.20
**No**	96	56.80	169	100.00

Binomial Proportion	
**FEMALE = Yes**	
**Proportion**	0.4320
**ASE**	0.0381
**95% Lower Conf Limit**	0.3573
**95% Upper Conf Limit**	0.5066
**Exact Conf Limits**	
**95% Lower Conf Limit**	0.3561
**95% Upper Conf Limit**	0.5102

Test of H0: Proportion = 0.5	
**ASE under H0**	0.0385
**Z**	-1.7692
**One-sided Pr < Z**	0.0384
**Two-sided Pr > |Z|**	0.0769

Sample Size = 169

Tukey Style Multiple Comparisons of Proportions

Compare Diff SE q q(.05) Conclude

Compare	Diff	SE	q	q(.05)	Conclude
2 vs 1	7.9	1.79	4.41	3.314	R
2 vs 3	3.46	1.91	1.81	3.314	A
3 vs 1	4.43	2.1	2.11	3.314	A

Reference: Biostatistical Analysis, Fourth Edition, Jerrold Zar, 1999, p564.

Tukey Style Multiple Comparisons of Proportions

Compare Diff SE q q(.01) Conclude

Compare	Diff	SE	q	q(.01)	Conclude
2 vs 1	7.9	1.79	4.41	4.12	R
2 vs 3	3.46	1.91	1.81	4.12	A
3 vs 1	4.43	2.1	2.11	4.12	A

Reference: Biostatistical Analysis, Fourth Edition, Jerrold Zar, 1999, p564.

Chi-Square Analysis for a 2x3 table

Tukey Style Multiple Comparisons of Proportions

Compare Diff SE q q(.001) Conclude

Compare	Diff	SE	q	q(.001)	Conclude
2 vs 1	7.9	1.79	4.41	5.063	A
2 vs 3	3.46	1.91	1.81	5.063	A
3 vs 1	4.43	2.1	2.11	5.063	A

Reference: Biostatistical Analysis, Fourth Edition, Jerrold Zar, 1999, p564.

Figure 8c-% Eggs Hatched by Group;

The FREQ Procedure

Frequency<br>Col Pct	Table of EGGS_HATCHED_YES by GROUP					
	EGGS_HATCHED_YES	GROUP				
		1=Control_with	2=Control_without	3=CNP	4=AgCNP	Total
	Yes	541<br>79.68	672<br>84.00	695<br>82.25	121<br>50.63	2029
	No	138<br>20.32	128<br>16.00	150<br>17.75	118<br>49.37	534
	Total	679	800	845	239	2563

Statistics for Table of EGGS_HATCHED_YES by GROUP

Statistic	DF	Value	Prob
Chi-Square	3	134.3220	<.0001
Likelihood Ratio Chi-Square	3	112.6797	<.0001
Mantel-Haenszel Chi-Square	1	33.8700	<.0001
Phi Coefficient		0.2289	
Contingency Coefficient		0.2232	
Cramer's V		0.2289	

Sample Size = 2563

The FREQ Procedure
GROUP=1=Control_with

EGGS_HATCHED_YES	Frequency	Percent	Cumulative Frequency	Cumulative Percent
**Yes**	541	79.68	541	79.68
**No**	138	20.32	679	100.00

Binomial Proportion	
**EGGS_HATCHED_YES = Yes**	
**Proportion**	0.7968
**ASE**	0.0154
**95% Lower Conf Limit**	0.7665
**95% Upper Conf Limit**	0.8270
**Exact Conf Limits**	
**95% Lower Conf Limit**	0.7645
**95% Upper Conf Limit**	0.8264

Test of H0: Proportion = 0.5	
**ASE under H0**	0.0192
**Z**	15.4657
**One-sided Pr > Z**	<.0001
**Two-sided Pr > |Z|**	<.0001

Sample Size = 679

Eggs Hatched with SE by Group

The FREQ Procedure
GROUP=2=Control_without

EGGS_HATCHED_YES	Frequency	Percent	Cumulative Frequency	Cumulative Percent
Yes	672	84.00	672	84.00
No	128	16.00	800	100.00

Binomial Proportion	
EGGS_HATCHED_YES = Yes	
Proportion	0.8400
ASE	0.0130
95% Lower Conf Limit	0.8146
95% Upper Conf Limit	0.8654
Exact Conf Limits	
95% Lower Conf Limit	0.8127
95% Upper Conf Limit	0.8647

Test of H0: Proportion = 0.5	
ASE under H0	0.0177
Z	19.2333
One-sided Pr > Z	<.0001
Two-sided Pr > |Z|	<.0001

Sample Size = 800

Eggs Hatched with SE by Group

The FREQ Procedure
GROUP=3=CNP

EGGS_HATCHED_YES	Frequency	Percent	Cumulative Frequency	Cumulative Percent
Yes	695	82.25	695	82.25
No	150	17.75	845	100.00

Binomial Proportion	
EGGS_HATCHED_YES = Yes	
Proportion	0.8225
ASE	0.0131
95% Lower Conf Limit	0.7967
95% Upper Conf Limit	0.8482
Exact Conf Limits	
95% Lower Conf Limit	0.7950
95% Upper Conf Limit	0.8477

Test of H0: Proportion = 0.5	
ASE under H0	0.0172
Z	18.7486
One-sided Pr > Z	<.0001
Two-sided Pr > |Z|	<.0001

Sample Size = 845

Eggs Hatched with SE by Group

The FREQ Procedure
GROUP=4=AgCNP

EGGS_HATCHED_YES	Frequency	Percent	Cumulative Frequency	Cumulative Percent
Yes	121	50.63	121	50.63
No	118	49.37	239	100.00

Binomial Proportion	
EGGS_HATCHED_YES = Yes	
Proportion	0.5063
ASE	0.0323
95% Lower Conf Limit	0.4429
95% Upper Conf Limit	0.5697
Exact Conf Limits	
95% Lower Conf Limit	0.4411
95% Upper Conf Limit	0.5713

Test of H0: Proportion = 0.5	
ASE under H0	0.0323
Z	0.1941
One-sided Pr > Z	0.4231
Two-sided Pr > |Z|	0.8461

Sample Size = 239

Tukey Style Multiple Comparisons of Proportions

Compare	Diff	SE	q	q(.001)	Conclude
2 vs 4	21.03	1.49	14.12	5.309	R
2 vs 1	3.22	1.06	3.03	5.309	A
2 vs 3	1.34	1	1.34	5.309	A
3 vs 4	19.7	1.48	13.31	5.309	R
3 vs 1	1.88	1.04	1.81	5.309	A
1 vs 4	17.82	1.52	11.72	5.309	R

Reference: Biostatistical Analysis, Fourth Edition, Jerrold Zar, 1999, p564.

Figure 8c-% Adults Emerged by Group;

The FREQ Procedure

Table of ADULTS_EMERGED_YES by GROUP

Frequency<br>Col Pct	ADULTS_EMERGED_YES	GROUP				
		1=Control_with	2=Control_without	3=CNP	4=AgCNP	Total
	Yes	392<br>57.73	493<br>61.63	525<br>62.13	100<br>41.84	1510
	No	287<br>42.27	307<br>38.38	320<br>37.87	139<br>58.16	1053
	Total	679	800	845	239	2563

Statistics for Table of ADULTS_EMERGED_YES by GROUP

Statistic	DF	Value	Prob
Chi-Square	3	35.2133	<.0001
Likelihood Ratio Chi-Square	3	34.6482	<.0001
Mantel-Haenszel Chi-Square	1	3.8378	0.0501
Phi Coefficient		0.1172	
Contingency Coefficient		0.1164	
Cramer's V		0.1172	

The FREQ Procedure
GROUP=1=Control_with

ADULTS_EMERGED_YES	Frequency	Percent	Cumulative Frequency	Cumulative Percent
**Yes**	392	57.73	392	57.73
**No**	287	42.27	679	100.00

Binomial Proportion	
**ADULTS_EMERGED_YES = Yes**	
**Proportion**	0.5773
**ASE**	0.0190
**95% Lower Conf Limit**	0.5402
**95% Upper Conf Limit**	0.6145
**Exact Conf Limits**	
**95% Lower Conf Limit**	0.5392
**95% Upper Conf Limit**	0.6148

Test of H0: Proportion = 0.5	
**ASE under H0**	0.0192
**Z**	4.0295
**One-sided Pr > Z**	<.0001
**Two-sided Pr > |Z|**	<.0001

Sample Size = 679

Adults Emerged with SE by Group

The FREQ Procedure
GROUP=2=Control_without

ADULTS_EMERGED_YES	Frequency	Percent	Cumulative Frequency	Cumulative Percent
**Yes**	493	61.63	493	61.63
**No**	307	38.38	800	100.00

Binomial Proportion	
**ADULTS_EMERGED_YES = Yes**	
**Proportion**	0.6163
**ASE**	0.0172
**95% Lower Conf Limit**	0.5826
**95% Upper Conf Limit**	0.6499
**Exact Conf Limits**	
**95% Lower Conf Limit**	0.5815
**95% Upper Conf Limit**	0.6501

Test of H0: Proportion = 0.5	
**ASE under H0**	0.0177
**Z**	6.5761
**One-sided Pr > Z**	<.0001
**Two-sided Pr > |Z|**	<.0001

Sample Size = 800

Adults Emerged with SE by Group

The FREQ Procedure
GROUP=3=CNP

ADULTS_EMERGED_YES	Frequency	Percent	Cumulative Frequency	Cumulative Percent
**Yes**	525	62.13	525	62.13
**No**	320	37.87	845	100.00

Binomial Proportion	
**ADULTS_EMERGED_YES = Yes**	
**Proportion**	0.6213
**ASE**	0.0167
**95% Lower Conf Limit**	0.5886
**95% Upper Conf Limit**	0.6540
**Exact Conf Limits**	
**95% Lower Conf Limit**	0.5876
**95% Upper Conf Limit**	0.6541

Test of H0: Proportion = 0.5	
**ASE under H0**	0.0172
**Z**	7.0522
**One-sided Pr > Z**	<.0001
**Two-sided Pr > |Z|**	<.0001

Sample Size = 845

Adults Emerged with SE by Group

The FREQ Procedure
GROUP=4=AgCNP

ADULTS_EMERGED_YES	Frequency	Percent	Cumulative Frequency	Cumulative Percent
**Yes**	100	41.84	100	41.84
**No**	139	58.16	239	100.00

Binomial Proportion	
**ADULTS_EMERGED_YES = Yes**	
**Proportion**	0.4184
**ASE**	0.0319
**95% Lower Conf Limit**	0.3559
**95% Upper Conf Limit**	0.4810
**Exact Conf Limits**	
**95% Lower Conf Limit**	0.3551
**95% Upper Conf Limit**	0.4837

Test of H0: Proportion = 0.5	
**ASE under H0**	0.0323
**Z**	-2.5227
**One-sided Pr < Z**	0.0058
**Two-sided Pr > |Z|**	0.0116

Sample Size = 239

Tukey Style Multiple Comparisons of Proportions

Compare	Diff	SE	q	q(.001)	Conclude
3 vs 4	11.69	1.48	7.9	5.309	R
3 vs 1	2.57	1.04	2.47	5.309	A
3 vs 2	0.3	1	0.3	5.309	A
2 vs 4	11.39	1.49	7.65	5.309	R
2 vs 1	2.27	1.06	2.14	5.309	A
1 vs 4	9.12	1.52	6	5.309	R

Reference: Biostatistical Analysis, Fourth Edition, Jerrold Zar, 1999, p564.

Figure 8c-% Larvae Pupae by Group;

Chi-Square Analysis for a 2x4 table

The FREQ Procedure

Table of LARVAE_PUPAE_YES by GROUP

Frequency<br>Col Pct	LARVAE_PUPAE_YES	GROUP				
		1=Control_with	2=Control_without	3=CNP	4=AgCNP	Total
	Yes	147<br>21.65	179<br>22.38	160<br>18.93	21<br>8.79	507
	No	532<br>78.35	621<br>77.63	685<br>81.07	218<br>91.21	2056
	Total	679	800	845	239	2563

Statistics for Table of LARVAE_PUPAE_YES by GROUP

Statistic	DF	Value	Prob
Chi-Square	3	23.4730	<.0001
Likelihood Ratio Chi-Square	3	27.0513	<.0001
Mantel-Haenszel Chi-Square	1	14.2410	0.0002
Phi Coefficient		0.0957	
Contingency Coefficient		0.0953	
Cramer's V		0.0957	

Sample Size = 2563

Larvae Pupae with SE by Group

The FREQ Procedure

GROUP=1=Control_with

LARVAE_PUPAE_YES	Frequency	Percent	Cumulative Frequency	Cumulative Percent
**Yes**	147	21.65	147	21.65
**No**	532	78.35	679	100.00

Binomial Proportion	
**LARVAE_PUPAE_YES = Yes**	
**Proportion**	0.2165
**ASE**	0.0158
**95% Lower Conf Limit**	0.1855
**95% Upper Conf Limit**	0.2475
**Exact Conf Limits**	
**95% Lower Conf Limit**	0.1861
**95% Upper Conf Limit**	0.2494

Test of H0: Proportion = 0.5	
**ASE under H0**	0.0192
**Z**	-14.7749
**One-sided Pr < Z**	<.0001
**Two-sided Pr > |Z|**	<.0001

Sample Size = 679

Larvae Pupae with SE by Group

The FREQ Procedure
GROUP=2=Control_without

LARVAE_PUPAE_YES	Frequency	Percent	Cumulative Frequency	Cumulative Percent
**Yes**	179	22.38	179	22.38
**No**	621	77.63	800	100.00

Binomial Proportion	
**LARVAE_PUPAE_YES = Yes**	
**Proportion**	0.2238
**ASE**	0.0147
**95% Lower Conf Limit**	0.1949
**95% Upper Conf Limit**	0.2526
**Exact Conf Limits**	
**95% Lower Conf Limit**	0.1953
**95% Upper Conf Limit**	0.2542

Test of H0: Proportion = 0.5	
**ASE under H0**	0.0177
**Z**	-15.6271
**One-sided Pr < Z**	<.0001
**Two-sided Pr > |Z|**	<.0001

Sample Size = 800

Larvae Pupae with SE by Group

The FREQ Procedure
GROUP=3=CNP

LARVAE_PUPAE_YES	Frequency	Percent	Cumulative Frequency	Cumulative Percent
**Yes**	160	18.93	160	18.93
**No**	685	81.07	845	100.00

Binomial Proportion

LARVAE_PUPAE_YES = Yes	
**Proportion**	0.1893
**ASE**	0.0135
**95% Lower Conf Limit**	0.1629
**95% Upper Conf Limit**	0.2158
**Exact Conf Limits**	
**95% Lower Conf Limit**	0.1635
**95% Upper Conf Limit**	0.2174

Test of H0: Proportion = 0.5	
**ASE under H0**	0.0172
**Z**	-18.0605
**One-sided Pr < Z**	<.0001
**Two-sided Pr > |Z|**	<.0001

Sample Size = 845

Larvae Pupae with SE by Group

The FREQ Procedure
GROUP=4=AgCNP

LARVAE_PUPAE_YES	Frequency	Percent	Cumulative Frequency	Cumulative Percent
**Yes**	21	8.79	21	8.79
**No**	218	91.21	239	100.00

Binomial Proportion	
**LARVAE_PUPAE_YES = Yes**	
**Proportion**	0.0879
**ASE**	0.0183
**95% Lower Conf Limit**	0.0520
**95% Upper Conf Limit**	0.1238
**Exact Conf Limits**	
**95% Lower Conf Limit**	0.0552
**95% Upper Conf Limit**	0.1312

Test of H0: Proportion = 0.5	
**ASE under H0**	0.0323
**Z**	-12.7429
**One-sided Pr < Z**	<.0001
**Two-sided Pr > |Z|**	<.0001

Sample Size = 239

Tukey Style Multiple Comparisons of Proportions

Compare	Diff	SE	q	q(.001)	Conclude
2 vs 4	10.84	1.49	7.28	5.309	R
2 vs 3	2.43	1	2.43	5.309	A
2 vs 1	0.5	1.06	0.47	5.309	A
1 vs 4	10.35	1.52	6.81	5.309	R
1 vs 3	1.94	1.04	1.86	5.309	A
3 vs 4	8.41	1.48	5.68	5.309	R

Reference: Biostatistical Analysis, Fourth Edition, Jerrold Zar, 1999, p564.

Figure 8d-% Female by Group;

Chi-Square Analysis for a 2x5 table

The FREQ Procedure

Table of FEMALE by GROUP

Frequency<br>Col Pct	FEMALE	GROUP				
		1=Control	2=Control	3=CNP	4=AgCNP	Total
	Yes	159<br>40.56	195<br>39.55	244<br>46.48	51<br>51.00	649
	No	233<br>59.44	298<br>60.45	281<br>53.52	49<br>49.00	861
	Total	392	493	525	100	1510

Statistics for Table of FEMALE by GROUP

Statistic	DF	Value	Prob
Chi-Square	3	8.5404	0.0361
Likelihood Ratio Chi-Square	3	8.5192	0.0364
Mantel-Haenszel Chi-Square	1	6.3126	0.0120
Phi Coefficient		0.0752	
Contingency Coefficient		0.0750	
Cramer's V		0.0752	

Sample Size = 1510

Proportion Female with SE by Group

The FREQ Procedure
GROUP=1=Control

FEMALE	Frequency	Percent	Cumulative Frequency	Cumulative Percent
Yes	159	40.56	159	40.56
No	233	59.44	392	100.00

Binomial Proportion	
FEMALE = Yes	
Proportion	0.4056
ASE	0.0248
95% Lower Conf Limit	0.3570
95% Upper Conf Limit	0.4542
Exact Conf Limits	
95% Lower Conf Limit	0.3566
95% Upper Conf Limit	0.4561

Test of H0: Proportion = 0.5	
ASE under H0	0.0253
Z	-3.7376
One-sided Pr < Z	<.0001
Two-sided Pr > |Z|	0.0002

Sample Size = 392

Proportion Female with SE by Group

The FREQ Procedure
GROUP=2=Control

FEMALE	Frequency	Percent	Cumulative Frequency	Cumulative Percent
Yes	195	39.55	195	39.55
No	298	60.45	493	100.00

Binomial Proportion	
FEMALE = Yes	
Proportion	0.3955
ASE	0.0220
95% Lower Conf Limit	0.3524
95% Upper Conf Limit	0.4387
Exact Conf Limits	
95% Lower Conf Limit	0.3521
95% Upper Conf Limit	0.4402

Test of H0: Proportion = 0.5	
ASE under H0	0.0225
Z	-4.6389
One-sided Pr < Z	<.0001
Two-sided Pr > |Z|	<.0001

Sample Size = 493

Proportion Female with SE by Group

The FREQ Procedure
GROUP=3=CNP

FEMALE	Frequency	Percent	Cumulative Frequency	Cumulative Percent
Yes	244	46.48	244	46.48
No	281	53.52	525	100.00

Binomial Proportion	
FEMALE = Yes	
Proportion	0.4648
ASE	0.0218
95% Lower Conf Limit	0.4221
95% Upper Conf Limit	0.5074
Exact Conf Limits	
95% Lower Conf Limit	0.4214
95% Upper Conf Limit	0.5085

Test of H0: Proportion = 0.5	
ASE under H0	0.0218
Z	-1.6148
One-sided Pr < Z	0.0532
Two-sided Pr > |Z|	0.1064

Sample Size = 525

Proportion Female with SE by Group

The FREQ Procedure
GROUP=4=AgCNP

FEMALE	Frequency	Percent	Cumulative Frequency	Cumulative Percent
Yes	51	51.00	51	51.00
No	49	49.00	100	100.00

Binomial Proportion	
FEMALE = Yes	
Proportion	0.5100
ASE	0.0500
95% Lower Conf Limit	0.4120
95% Upper Conf Limit	0.6080
Exact Conf Limits	
95% Lower Conf Limit	0.4080
95% Upper Conf Limit	0.6114

Test of H0: Proportion = 0.5	
ASE under H0	0.0500
Z	0.2000
One-sided Pr > Z	0.4207
Two-sided Pr > |Z|	0.8415

Sample Size = 100

Tukey Style Multiple Comparisons of Proportions

Compare	Diff	SE	q	q(.05)	Conclude
4 vs 2	6.59	2.22	2.97	3.633	A
4 vs 1	6	2.26	2.65	3.633	A
4 vs 3	2.58	2.21	1.17	3.633	A
3 vs 2	4	1.27	3.15	3.633	A
3 vs 1	3.41	1.35	2.53	3.633	A
1 vs 2	0.59	1.37	0.43	3.633	A

Reference: Biostatistical Analysis, Fourth Edition, Jerrold Zar, 1999, p564.

OK, this is interesting. Although the overall chi-square was significant, after adjusting the p-values for multiple comparisons, no comparison met the significance test at the α = 0.05 level.
