# Supplementary material for: Exposure to nanoceria impacts larval survival, life history traits and fecundity of Aedes aegypti
Source: PLoS Negl Trop Dis. 2020 Sep 25;14(9):e0008654. doi: 10.1371/journal.pntd.0008654 (PMC7540862; doi:10.1371/journal.pntd.0008654)
Supplement: S5 File — (PDF) [file pntd.0008654.s009.pdf]

Figure 9a-Body length by Group;

The NPAR1WAY Procedure

Wilcoxon Scores (Rank Sums) for Variable BODY_LENGTH Classified by Variable GROUP

GROUP	N	Sum of Scores	Expected Under H0	Std Dev Under H0	Mean Score
**1=Control**	4	34.0	32.0	7.338094	8.500000
**2=CNP**	6	41.0	48.0	8.129312	6.833333
**3=AgCNP**	5	45.0	40.0	7.822434	9.000000

Average scores were used for ties.

Kruskal-Wallis Test

Chi-Square	DF	Pr > ChiSq
0.7717	2	0.6799

The MEANS Procedure
GROUP=1=Control

Analysis Variable : BODY_LENGTH

N	Minimum	Maximum	Mean	Median	Std Dev	Std Error	Lower 95% CL for Mean	Upper 95% CL for Mean
4	2.2800000	2.8000000	2.5200000	2.5000000	0.2286190	0.1143095	2.1562161	2.8837839

GROUP=2=CNP

Analysis Variable : BODY_LENGTH

N	Minimum	Maximum	Mean	Median	Std Dev	Std Error	Lower 95% CL for Mean	Upper 95% CL for Mean
6	2.3200000	2.4000000	2.3866667	2.4000000	0.0326599	0.0133333	2.3523922	2.4209411

GROUP=3=AgCNP

Analysis Variable : BODY_LENGTH

N	Minimum	Maximum	Mean	Median	Std Dev	Std Error	Lower 95% CL for Mean	Upper 95% CL for Mean
5	2.0000000	2.8000000	2.5440000	2.8000000	0.3683477	0.1647301	2.0866360	3.0013640

Figure 9a-Body width by Group;

The NPAR1WAY Procedure

Wilcoxon Scores (Rank Sums) for Variable BODY_WIDTH Classified by Variable GROUP

GROUP	N	Sum of Scores	Expected Under H0	Std Dev Under H0	Mean Score
**1=Control**	4	42.00	32.0	6.400149	10.500
**2=CNP**	6	40.50	48.0	7.090235	6.750
**3=AgCNP**	5	37.50	40.0	6.822582	7.500

Average scores were used for ties.

Kruskal-Wallis Test

Chi-Square	DF	Pr > ChiSq
2.5512	2	0.2793

The MEANS Procedure

GROUP=1=Control

Analysis Variable : BODY_WIDTH

N	Minimum	Maximum	Mean	Median	Std Dev	Std Error	Lower 95% CL for Mean	Upper 95% CL for Mean
4	1.0000000	1.0000000	1.0000000	1.0000000	0	0	.	.

GROUP=2=CNP

Analysis Variable : BODY_WIDTH

N	Minimum	Maximum	Mean	Median	Std Dev	Std Error	Lower 95% CL for Mean	Upper 95% CL for Mean
6	0.7500000	1.0000000	0.8750000	0.8750000	0.1369306	0.0559017	0.7313001	1.0186999

GROUP=3=AgCNP

Analysis Variable : BODY_WIDTH

N	Minimum	Maximum	Mean	Median	Std Dev	Std Error	Lower 95% CL for Mean	Upper 95% CL for Mean
5	0.4000000	1.0000000	0.8560000	1.0000000	0.2601538	0.1163443	0.5329764	1.1790236

Figure 9a-Wing length by Group;

The NPAR1WAY Procedure

Van der Waerden Scores (Normal) for Variable WING_LENGTH Classified by Variable GROUP

GROUP	N	Sum of Scores	Expected Under H0	Std Dev Under H0	Mean Score
**1=Control**	4	-0.202077	0.0	1.426732	-0.050519
**2=CNP**	6	-2.051709	0.0	1.580567	-0.341951
**3=AgCNP**	5	2.253786	0.0	1.520901	0.450757

Average scores were used for ties.

Van der Waerden One-Way Analysis

Chi-Square	DF	Pr > ChiSq
2.4897	2	0.2880

GROUP=1=Control

Analysis Variable : WING_LENGTH

N	Minimum	Maximum	Mean	Median	Std Dev	Std Error	Lower 95% CL for Mean	Upper 95% CL for Mean
4	2.8000000	3.4000000	3.1000000	3.1000000	0.2581989	0.1290994	2.6891479	3.5108521

GROUP=2=CNP

Analysis Variable : WING_LENGTH

N	Minimum	Maximum	Mean	Median	Std Dev	Std Error	Lower 95% CL for Mean	Upper 95% CL for Mean
6	2.8000000	3.2000000	3.0166667	3.0500000	0.1834848	0.0749074	2.8241112	3.2092221

GROUP=3=AgCNP

Analysis Variable : WING_LENGTH

N	Minimum	Maximum	Mean	Median	Std Dev	Std Error	Lower 95% CL for Mean	Upper 95% CL for Mean
5	2.7000000	3.4000000	3.2600000	3.4000000	0.3130495	0.1400000	2.8712977	3.6487023

Figure 9a-Wing width by Group;

The NPAR1WAY Procedure

Wilcoxon Scores (Rank Sums) for Variable WING_WIDTH Classified by Variable GROUP

GROUP	N	Sum of Scores	Expected Under H0	Std Dev Under H0	Mean Score
**1=Control**	4	29.0	32.0	6.350853	7.250000
**2=CNP**	6	47.0	48.0	7.035624	7.833333
**3=AgCNP**	5	44.0	40.0	6.770032	8.800000

Average scores were used for ties.

Kruskal-Wallis Test

Chi-Square	DF	Pr > ChiSq
0.4085	2	0.8153

The MEANS Procedure
GROUP=1=Control

Analysis Variable : WING_WIDTH

N	Minimum	Maximum	Mean	Median	Std Dev	Std Error	Lower 95% CL for Mean	Upper 95% CL for Mean
4	0.8000000	0.9000000	0.8250000	0.8000000	0.0500000	0.0250000	0.7454388	0.9045612

GROUP=2=CNP

Analysis Variable : WING_WIDTH

N	Minimum	Maximum	Mean	Median	Std Dev	Std Error	Lower 95% CL for Mean	Upper 95% CL for Mean
6	0.8000000	0.9000000	0.8333333	0.8000000	0.0516398	0.0210819	0.7791407	0.8875260

GROUP=3=AgCNP

Analysis Variable : WING_WIDTH

N	Minimum	Maximum	Mean	Median	Std Dev	Std Error	Lower 95% CL for Mean	Upper 95% CL for Mean
5	0.8000000	1.0000000	0.8600000	0.8000000	0.0894427	0.0400000	0.7489422	0.9710578

Figure 9b-Body Length by Group;

For all hypothesis tests in 9b, we will use the nonparametric Kruskal-Wallis test due to small sample size.

The NPAR1WAY Procedure

Analysis of Variance for Variable BODY_LENGTH
Classified by Variable GROUP

GROUP	N	Mean
**1=Control_with**	6	2.346667
**2=Control_without**	6	2.253333
**3=CNP**	6	2.200000
**4=AgCNP**	3	2.866667

Kruskal-Wallis Test

Chi-Square	DF	Pr > ChiSq
6.8614	3	0.0764

Did not reach formal statistical significance so multiple comparisons not indicated.

The MEANS Procedure
GROUP=1=Control_with

Analysis Variable : BODY_LENGTH

N	Minimum	Maximum	Mean	Median	Std Dev	Std Error	Lower 95% CL for Mean	Upper 95% CL for Mean
6	2.0000000	2.8000000	2.3466667	2.3400000	0.2673325	0.1091380	2.0661184	2.6272149

GROUP=2=Control_without

Analysis Variable : BODY_LENGTH

N	Minimum	Maximum	Mean	Median	Std Dev	Std Error	Lower 95% CL for Mean	Upper 95% CL for Mean
6	2.0000000	2.8000000	2.2533333	2.1600000	0.3216623	0.1313181	1.9157694	2.5908973

GROUP=3=CNP

Analysis Variable : BODY_LENGTH

N	Minimum	Maximum	Mean	Median	Std Dev	Std Error	Lower 95% CL for Mean	Upper 95% CL for Mean
6	1.9000000	2.4000000	2.2000000	2.2000000	0.1673320	0.0683130	2.0243958	2.3756042

GROUP=4=AgCNP

Analysis Variable : BODY_LENGTH

N	Minimum	Maximum	Mean	Median	Std Dev	Std Error	Lower 95% CL for Mean	Upper 95% CL for Mean
3	2.6000000	3.2000000	2.8666667	2.8000000	0.3055050	0.1763834	2.1077501	3.6255833

Figure 9b-Body Width by Group;

The NPAR1WAY Procedure

Analysis of Variance for Variable BODY_WIDTH Classified by Variable GROUP

GROUP	N	Mean
**1=Control_with**	6	0.865000
**2=Control_without**	6	0.838333
**3=CNP**	6	0.800000
**4=AgCNP**	3	0.933333

Kruskal-Wallis Test

Chi-Square	DF	Pr > ChiSq
1.0629	3	0.7860

Did not reach formal statistical significance so multiple comparisons not indicated.

GROUP=1=Control_with

Analysis Variable : BODY_WIDTH

N	Minimum	Maximum	Mean	Median	Std Dev	Std Error	Lower 95% CL for Mean	Upper 95% CL for Mean
6	0.7200000	1.0000000	0.8650000	0.8750000	0.1482903	0.0605392	0.7093789	1.0206211

GROUP=2=Control_without

Analysis Variable : BODY_WIDTH								
**N**	**Minimum**	**Maximum**	**Mean**	**Median**	**Std Dev**	**Std Error**	**Lower 95% CL for Mean**	**Upper 95% CL for Mean**
6	0.4000000	1.0000000	0.8383333	0.9400000	0.2366784	0.0966236	0.5899546	1.0867121

GROUP=3=CNP

Analysis Variable : BODY_WIDTH								
**N**	**Minimum**	**Maximum**	**Mean**	**Median**	**Std Dev**	**Std Error**	**Lower 95% CL for Mean**	**Upper 95% CL for Mean**
6	0.6000000	1.0000000	0.8000000	0.8000000	0.2190890	0.0894427	0.5700802	1.0299198

GROUP=4=AgCNP

Analysis Variable : BODY_WIDTH								
**N**	**Minimum**	**Maximum**	**Mean**	**Median**	**Std Dev**	**Std Error**	**Lower 95% CL for Mean**	**Upper 95% CL for Mean**
3	0.8000000	1.0000000	0.9333333	1.0000000	0.1154701	0.0666667	0.6464898	1.2201768

Figure 9b-Wing length by Group;

Analysis of Variance for Variable WING_LENGTH Classified by Variable GROUP

GROUP	N	Mean
**1=Control_with**	6	2.873333
**2=Control_without**	6	2.816667
**3=CNP**	6	3.266667
**4=AgCNP**	3	3.233333

Kruskal-Wallis Test

Chi-Square	DF	Pr > ChiSq
9.8105	3	0.0202

GROUP=1=Control_with

Analysis Variable : WING_LENGTH

N	Minimum	Maximum	Mean	Median	Std Dev	Std Error	Lower 95% CL for Mean	Upper 95% CL for Mean
6	2.6000000	3.5400000	2.8733333	2.7500000	0.3592585	0.1466667	2.4963147	3.2503520

GROUP=2=Control_without

Analysis Variable : WING_LENGTH

N	Minimum	Maximum	Mean	Median	Std Dev	Std Error	Lower 95% CL for Mean	Upper 95% CL for Mean
6	2.5000000	3.0000000	2.8166667	2.9000000	0.2228602	0.0909823	2.5827892	3.0505441

GROUP=3=CNP

Analysis Variable : WING_LENGTH

N	Minimum	Maximum	Mean	Median	Std Dev	Std Error	Lower 95% CL for Mean	Upper 95% CL for Mean
6	3.2000000	3.4000000	3.2666667	3.2500000	0.0816497	0.0333333	3.1809806	3.3523527

GROUP=4=AgCNP

Analysis Variable : WING_LENGTH

N	Minimum	Maximum	Mean	Median	Std Dev	Std Error	Lower 95% CL for Mean	Upper 95% CL for Mean
3	3.0000000	3.5000000	3.2333333	3.2000000	0.2516611	0.1452966	2.6081724	3.8584943

GROUP
1=Control_with
2=Control_without
3=CNP
4=AgCNP

Group sample sizes not equal, or some ranks tied. Performed Dunn's test, alpha=0.05

Comparison group = GROUP_NEW

Compare	Diff	SE	q	q(0.05)	Conclude
3 vs 2	9.5	3.54	2.68	2.638	Reject
3 vs 1	8.17	3.54	2.31	2.638	Do not reject
3 vs 4	Do not reject (within non-sig. comparison)				
4 vs 2	8.08	4.34	1.86	2.638	Do not reject
4 vs 1	Do not reject (within non-sig. comparison)				
1 vs 2	Do not reject (within non-sig. comparison)				

Note: "Do not reject (within non-sig. comparison)" indicates that any comparison within the range of a non-significant comparison must also be non-significant.

Reference: Biostatistical Analysis, 4th Edition, J. Zar, 2010.

Figure 9b-Wing width by Group;

The NPAR1WAY Procedure

Wilcoxon Scores (Rank Sums) for Variable WING_WIDTH Classified by Variable GROUP

GROUP	N	Sum of Scores	Expected Under H0	Std Dev Under H0	Mean Score
1=Control_with	6	63.50	66.0	11.549583	10.583333
2=Control_without	6	40.50	66.0	11.549583	6.750000
3=CNP	6	85.50	66.0	11.549583	14.250000
4=AgCNP	3	41.50	33.0	8.946268	13.833333

Average scores were used for ties.

Kruskal-Wallis Test

Chi-Square	DF	Pr > ChiSq
6.3253	3	0.0968

Did not reach formal statistical significance so multiple comparisons not indicated.

The MEANS Procedure
GROUP=1=Control_with

Analysis Variable : WING_WIDTH

N	Minimum	Maximum	Mean	Median	Std Dev	Std Error	Lower 95% CL for Mean	Upper 95% CL for Mean
6	0.6000000	1.0000000	0.8000000	0.8000000	0.1264911	0.0516398	0.6672557	0.9327443

GROUP=2=Control_without

Analysis Variable : WING_WIDTH

N	Minimum	Maximum	Mean	Median	Std Dev	Std Error	Lower 95% CL for Mean	Upper 95% CL for Mean
6	0.7000000	0.8000000	0.7500000	0.7500000	0.0547723	0.0223607	0.6925200	0.8074800

GROUP=3=CNP

Analysis Variable : WING_WIDTH

N	Minimum	Maximum	Mean	Median	Std Dev	Std Error	Lower 95% CL for Mean	Upper 95% CL for Mean
6	0.8000000	0.9000000	0.8500000	0.8500000	0.0547723	0.0223607	0.7925200	0.9074800

GROUP=4=AgCNP

Analysis Variable : WING_WIDTH

N	Minimum	Maximum	Mean	Median	Std Dev	Std Error	Lower 95% CL for Mean	Upper 95% CL for Mean
3	0.8000000	1.0000000	0.8666667	0.8000000	0.1154701	0.0666667	0.5798232	1.1535102
