# Supplementary material for: Exposure to nanoceria impacts larval survival, life history traits and fecundity of Aedes aegypti
Source: PLoS Negl Trop Dis. 2020 Sep 25;14(9):e0008654. doi: 10.1371/journal.pntd.0008654 (PMC7540862; doi:10.1371/journal.pntd.0008654)
Supplement: S6 File — (PDF) [file pntd.0008654.s010.pdf]

Figure 10a- Egg Weight by Group;

Egg Weight by Group

The GLM Procedure

Dependent Variable: EGG_WT

Source	DF	Sum of Squares	Mean Square	F Value	Pr > F
**Model**	4	460.208571	115.052143	5.73	0.0015
**Error**	30	601.851429	20.061714		
**Corrected Total**	34	1062.060000			

The MEANS Procedure
GROUP=1=Control

Analysis Variable : EGG_WT

N	Minimum	Maximum	Mean	Median	Std Dev	Std Error	Lower 95% CL for Mean	Upper 95% CL for Mean
7	6.7000000	27.9000000	18.3571429	17.6000000	7.3011089	2.7595598	11.6047434	25.1095423

GROUP=2=CNP

Analysis Variable : EGG_WT

N	Minimum	Maximum	Mean	Median	Std Dev	Std Error	Lower 95% CL for Mean	Upper 95% CL for Mean
7	8.7000000	25.2000000	15.2857143	13.5000000	5.6212183	2.1246208	10.0869544	20.4844741

GROUP=3=AgCNP

Analysis Variable : EGG_WT

N	Minimum	Maximum	Mean	Median	Std Dev	Std Error	Lower 95% CL for Mean	Upper 95% CL for Mean
7	4.4000000	9.5000000	7.4714286	7.9000000	1.6790020	0.6346031	5.9186107	9.0242464

GROUP=4=CNP+AgNO3

Analysis Variable : EGG_WT

N	Minimum	Maximum	Mean	Median	Std Dev	Std Error	Lower 95% CL for Mean	Upper 95% CL for Mean
7	8.1000000	15.8000000	12.2285714	12.5000000	2.8388462	1.0729830	9.6030766	14.8540662

GROUP=5=AgNO3

Analysis Variable : EGG_WT

N	Minimum	Maximum	Mean	Median	Std Dev	Std Error	Lower 95% CL for Mean	Upper 95% CL for Mean
7	8.0000000	14.2000000	12.1571429	12.1000000	2.1274845	0.8041136	10.1895478	14.1247379

Egg Weight by Group

The GLM Procedure
Least Squares Means
Adjustment for Multiple Comparisons: Tukey

GROUP	EGG_WT LSMEAN	LSMEAN Number
**1=Control**	18.3571429	1
**2=CNP**	15.2857143	2
**3=AgCNP**	7.4714286	3
**4=CNP+AgNO3**	12.2285714	4
**5=AgNO3**	12.1571429	5

Least Squares Means for effect GROUP
Pr > |t| for H0: LSMean(i)=LSMean(j)
Dependent Variable: EGG_WT

i/j	1	2	3	4	5
**1**		0.7034	0.0007	0.1041	0.0979
**2**	0.7034		0.0213	0.7069	0.6892
**3**	0.0007	0.0213		0.2965	0.3107
**4**	0.1041	0.7069	0.2965		1.0000
**5**	0.0979	0.6892	0.3107	1.0000	

Figure 10c-% Eggs hatched

The FREQ Procedure

Table of EGGS_HATCHED_YES by GROUP

Frequency<br>Col Pct	EGGS_HATCHED_YES	GROUP					
		1=Control	2=CNP	3=AgCNP	4=CNP+AgNO3	5=AgNO3	Total
	Yes	527<br>87.83	586<br>83.71	492<br>82.00	577<br>82.43	527<br>87.83	2709
	No	73<br>12.17	114<br>16.29	108<br>18.00	123<br>17.57	73<br>12.17	491
	Total	600	700	600	700	600	3200

Statistics for Table of EGGS_HATCHED_YES by GROUP

Statistic	DF	Value	Prob
Chi-Square	4	15.7366	0.0034
Likelihood Ratio Chi-Square	4	16.0806	0.0029
Mantel-Haenszel Chi-Square	1	0.1005	0.7512
Phi Coefficient		0.0701	
Contingency Coefficient		0.0700	
Cramer's V		0.0701	

Sample Size = 3200

Proportion Eggs Hatched with SE by Group

The FREQ Procedure
GROUP=1=Control

EGGS_HATCHED_YES	Frequency	Percent	Cumulative Frequency	Cumulative Percent
**Yes**	527	87.83	527	87.83
**No**	73	12.17	600	100.00

Binomial Proportion	
**EGGS_HATCHED_YES = Yes**	
**Proportion**	0.8783
**ASE**	0.0133
**95% Lower Conf Limit**	0.8522
**95% Upper Conf Limit**	0.9045
**Exact Conf Limits**	
**95% Lower Conf Limit**	0.8495
**95% Upper Conf Limit**	0.9034

Test of H0: Proportion = 0.5	
**ASE under H0**	0.0204
**Z**	18.5345
**One-sided Pr > Z**	<.0001
**Two-sided Pr > |Z|**	<.0001

Sample Size = 600

Proportion Eggs Hatched with SE by Group

The FREQ Procedure
GROUP=2=CNP

EGGS_HATCHED_YES	Frequency	Percent	Cumulative Frequency	Cumulative Percent
Yes	586	83.71	586	83.71
No	114	16.29	700	100.00

Binomial Proportion	
EGGS_HATCHED_YES = Yes	
Proportion	0.8371
ASE	0.0140
95% Lower Conf Limit	0.8098
95% Upper Conf Limit	0.8645
Exact Conf Limits	
95% Lower Conf Limit	0.8077
95% Upper Conf Limit	0.8637

Test of H0: Proportion = 0.5	
ASE under H0	0.0189
Z	17.8399
One-sided Pr > Z	<.0001
Two-sided Pr > |Z|	<.0001

Sample Size = 700

Proportion Eggs Hatched with SE by Group

The FREQ Procedure
GROUP=3=AgCNP

EGGS_HATCHED_YES	Frequency	Percent	Cumulative Frequency	Cumulative Percent
Yes	492	82.00	492	82.00
No	108	18.00	600	100.00

Binomial Proportion	
EGGS_HATCHED_YES = Yes	
Proportion	0.8200
ASE	0.0157
95% Lower Conf Limit	0.7893
95% Upper Conf Limit	0.8507
Exact Conf Limits	
95% Lower Conf Limit	0.7869
95% Upper Conf Limit	0.8499

Test of H0: Proportion = 0.5	
ASE under H0	0.0204
Z	15.6767
One-sided Pr > Z	<.0001
Two-sided Pr > |Z|	<.0001

Sample Size = 600

Proportion Eggs Hatched with SE by Group

The FREQ Procedure
GROUP=4=CNP+AgNO3

EGGS_HATCHED_YES	Frequency	Percent	Cumulative Frequency	Cumulative Percent
**Yes**	577	82.43	577	82.43
**No**	123	17.57	700	100.00

Binomial Proportion	
**EGGS_HATCHED_YES = Yes**	
**Proportion**	0.8243
**ASE**	0.0144
**95% Lower Conf Limit**	0.7961
**95% Upper Conf Limit**	0.8525
**Exact Conf Limits**	
**95% Lower Conf Limit**	0.7940
**95% Upper Conf Limit**	0.8518

Test of H0: Proportion = 0.5	
**ASE under H0**	0.0189
**Z**	17.1596
**One-sided Pr > Z**	<.0001
**Two-sided Pr > |Z|**	<.0001

Sample Size = 700

Proportion Eggs Hatched with SE by Group

The FREQ Procedure
GROUP=5=AgNO3

EGGS_HATCHED_YES	Frequency	Percent	Cumulative Frequency	Cumulative Percent
Yes	527	87.83	527	87.83
No	73	12.17	600	100.00

Binomial Proportion

EGGS_HATCHED_YES = Yes	
Proportion	0.8783
ASE	0.0133
95% Lower Conf Limit	0.8522
95% Upper Conf Limit	0.9045
Exact Conf Limits	
95% Lower Conf Limit	0.8495
95% Upper Conf Limit	0.9034

Test of H0: Proportion = 0.5	
ASE under H0	0.0204
Z	18.5345
One-sided Pr > Z	<.0001
Two-sided Pr > |Z|	<.0001

Sample Size = 600

Chi-Square Analysis for a 2x5 table

Tukey Style Multiple Comparisons of Proportions

Compare	Diff	SE	q	q(.05)	Conclude
5 vs 3	4.68	1.17	4	3.585	R
5 vs 4	4.35	1.13	3.85	3.585	R
5 vs 2	3.37	1.13	2.98	3.585	A
5 vs 1	0	1.17	0	3.585	A
1 vs 3	4.68	1.17	4	3.585	R
1 vs 4	4.35	1.13	3.85	3.585	R
1 vs 2	3.37	1.13	2.98	3.585	A
2 vs 3	1.31	1.13	1.16	3.585	A
2 vs 4	0.98	1.08	0.91	3.585	A
4 vs 3	0.33	1.13	0.29	3.585	A

Reference: Biostatistical Analysis, Fourth Edition, Jerrold Zar, 1999, p564.

Tukey Style Multiple Comparisons of Proportions

Compare	Diff	SE	q	q(.01)	Conclude
5 vs 3	4.68	1.17	4	4.603	A
5 vs 4	4.35	1.13	3.85	4.603	A
5 vs 2	3.37	1.13	2.98	4.603	A
5 vs 1	0	1.17	0	4.603	A
1 vs 3	4.68	1.17	4	4.603	A
1 vs 4	4.35	1.13	3.85	4.603	A
1 vs 2	3.37	1.13	2.98	4.603	A
2 vs 3	1.31	1.13	1.16	4.603	A
2 vs 4	0.98	1.08	0.91	4.603	A
4 vs 3	0.33	1.13	0.29	4.603	A

Reference: Biostatistical Analysis, Fourth Edition, Jerrold Zar, 1999, p564.

Figure 10c-% Adults Emerged

The FREQ Procedure

Frequency<br>Col Pct	Table of ADULTS_EMERGED_YES by GROUP						
	ADULTS_EMERGED_YES	GROUP					
		1=Control	2=CNP	3=AgCNP	4=CNP+AgNO3	5=AgNO3	Total
	Yes	458<br>76.33	518<br>74.00	394<br>65.67	492<br>70.29	464<br>77.33	2326
	No	142<br>23.67	182<br>26.00	206<br>34.33	208<br>29.71	136<br>22.67	874
	Total	600	700	600	700	600	3200

Statistics for Table of ADULTS_EMERGED_YES by GROUP

Statistic	DF	Value	Prob
Chi-Square	4	28.0790	<.0001
Likelihood Ratio Chi-Square	4	27.7424	<.0001
Mantel-Haenszel Chi-Square	1	0.1592	0.6899
Phi Coefficient		0.0937	
Contingency Coefficient		0.0933	
Cramer's V		0.0937	

Sample Size = 3200

Proportion Adults Emerged with SE by Group

The FREQ Procedure
GROUP=1=Control

ADULTS_EMERGED_YES	Frequency	Percent	Cumulative Frequency	Cumulative Percent
**Yes**	458	76.33	458	76.33
**No**	142	23.67	600	100.00

Binomial Proportion	
**ADULTS_EMERGED_YES = Yes**	
**Proportion**	0.7633
**ASE**	0.0174
**95% Lower Conf Limit**	0.7293
**95% Upper Conf Limit**	0.7973
**Exact Conf Limits**	
**95% Lower Conf Limit**	0.7272
**95% Upper Conf Limit**	0.7968

Test of H0: Proportion = 0.5	
**ASE under H0**	0.0204
**Z**	12.9006
**One-sided Pr > Z**	<.0001
**Two-sided Pr > |Z|**	<.0001

Sample Size = 600

Proportion Adults Emerged with SE by Group

The FREQ Procedure
GROUP=2=CNP

ADULTS_EMERGED_YES	Frequency	Percent	Cumulative Frequency	Cumulative Percent
**Yes**	518	74.00	518	74.00
**No**	182	26.00	700	100.00

Binomial Proportion	
**ADULTS_EMERGED_YES = Yes**	
**Proportion**	0.7400
**ASE**	0.0166
**95% Lower Conf Limit**	0.7075
**95% Upper Conf Limit**	0.7725
**Exact Conf Limits**	
**95% Lower Conf Limit**	0.7058
**95% Upper Conf Limit**	0.7721

Test of H0: Proportion = 0.5	
**ASE under H0**	0.0189
**Z**	12.6996
**One-sided Pr > Z**	<.0001
**Two-sided Pr > |Z|**	<.0001

Sample Size = 700

Proportion Adults Emerged with SE by Group

The FREQ Procedure
GROUP=3=AgCNP

ADULTS_EMERGED_YES	Frequency	Percent	Cumulative Frequency	Cumulative Percent
**Yes**	394	65.67	394	65.67
**No**	206	34.33	600	100.00

Binomial Proportion	
**ADULTS_EMERGED_YES = Yes**	
**Proportion**	0.6567
**ASE**	0.0194
**95% Lower Conf Limit**	0.6187
**95% Upper Conf Limit**	0.6947
**Exact Conf Limits**	
**95% Lower Conf Limit**	0.6171
**95% Upper Conf Limit**	0.6946

Test of H0: Proportion = 0.5	
**ASE under H0**	0.0204
**Z**	7.6751
**One-sided Pr > Z**	<.0001
**Two-sided Pr > |Z|**	<.0001

Sample Size = 600

Proportion Adults Emerged with SE by Group

The FREQ Procedure
GROUP=4=CNP+AgNO3

ADULTS_EMERGED_YES	Frequency	Percent	Cumulative Frequency	Cumulative Percent
**Yes**	492	70.29	492	70.29
**No**	208	29.71	700	100.00

Binomial Proportion	
**ADULTS_EMERGED_YES = Yes**	
**Proportion**	0.7029
**ASE**	0.0173
**95% Lower Conf Limit**	0.6690
**95% Upper Conf Limit**	0.7367
**Exact Conf Limits**	
**95% Lower Conf Limit**	0.6675
**95% Upper Conf Limit**	0.7365

Test of H0: Proportion = 0.5	
**ASE under H0**	0.0189
**Z**	10.7342
**One-sided Pr > Z**	<.0001
**Two-sided Pr > |Z|**	<.0001

Sample Size = 700

Proportion Adults Emerged with SE by Group

The FREQ Procedure
GROUP=5=AgNO3

ADULTS_EMERGED_YES	Frequency	Percent	Cumulative Frequency	Cumulative Percent
Yes	464	77.33	464	77.33
No	136	22.67	600	100.00

Binomial Proportion	
ADULTS_EMERGED_YES = Yes	
Proportion	0.7733
ASE	0.0171
95% Lower Conf Limit	0.7398
95% Upper Conf Limit	0.8068
Exact Conf Limits	
95% Lower Conf Limit	0.7377
95% Upper Conf Limit	0.8063

Test of H0: Proportion = 0.5	
ASE under H0	0.0204
Z	13.3905
One-sided Pr > Z	<.0001
Two-sided Pr > |Z|	<.0001

Sample Size = 600

Tukey Style Multiple Comparisons of Proportions

Compare	Diff	SE	q	q(.01)	Conclude
5 vs 3	7.43	1.17	6.35	4.603	R
5 vs 4	4.59	1.13	4.06	4.603	A
5 vs 2	2.22	1.13	1.96	4.603	A
5 vs 1	0.68	1.17	0.58	4.603	A
1 vs 3	6.75	1.17	5.77	4.603	R
1 vs 4	3.91	1.13	3.46	4.603	A
1 vs 2	1.54	1.13	1.36	4.603	A
2 vs 3	5.21	1.13	4.61	4.603	R
2 vs 4	2.37	1.08	2.2	4.603	A
4 vs 3	2.84	1.13	2.51	4.603	A

Tukey Style Multiple Comparisons of Proportions

Compare	Diff	SE	q	q(.001)	Conclude
5 vs 3	7.43	1.17	6.35	5.484	R
5 vs 4	4.59	1.13	4.06	5.484	A
5 vs 2	2.22	1.13	1.96	5.484	A
5 vs 1	0.68	1.17	0.58	5.484	A
1 vs 3	6.75	1.17	5.77	5.484	R
1 vs 4	3.91	1.13	3.46	5.484	A
1 vs 2	1.54	1.13	1.36	5.484	A
2 vs 3	5.21	1.13	4.61	5.484	A
2 vs 4	2.37	1.08	2.2	5.484	A
4 vs 3	2.84	1.13	2.51	5.484	A

Reference: Biostatistical Analysis, Fourth Edition, Jerrold Zar, 1999, p564.

Figure 10c-% Larvae Pupae

Chi-Square Analysis for a 2x5 table

The FREQ Procedure

Frequency / Col Pct	Table of LARVAE_PUPAE_YES by GROUP						
	LARVAE_PUPAE_YES	GROUP					
		1=Control	2=CNP	3=AgCNP	4=CNP+AgNO3	5=AgNO3	Total
	Yes	69	68	98	85	63	383
		11.50	9.71	16.33	12.14	10.50	
	No	531	632	502	615	537	2817
		88.50	90.29	83.67	87.86	89.50	
	Total	600	700	600	700	600	3200

Statistics for Table of LARVAE_PUPAE_YES by GROUP

Statistic	DF	Value	Prob
Chi-Square	4	15.5985	0.0036
Likelihood Ratio Chi-Square	4	14.8848	0.0049
Mantel-Haenszel Chi-Square	1	0.0383	0.8449
Phi Coefficient		0.0698	
Contingency Coefficient		0.0696	
Cramer's V		0.0698	

Sample Size = 3200

Proportion Larvae-Pupae with SE by Group

The FREQ Procedure
GROUP=1=Control

LARVAE_PUPAE_YES	Frequency	Percent	Cumulative Frequency	Cumulative Percent
**Yes**	69	11.50	69	11.50
**No**	531	88.50	600	100.00

Binomial Proportion	
**LARVAE_PUPAE_YES = Yes**	
**Proportion**	0.1150
**ASE**	0.0130
**95% Lower Conf Limit**	0.0895
**95% Upper Conf Limit**	0.1405
**Exact Conf Limits**	
**95% Lower Conf Limit**	0.0906
**95% Upper Conf Limit**	0.1433

Test of H0: Proportion = 0.5	
**ASE under H0**	0.0204
**Z**	-18.8611
**One-sided Pr < Z**	<.0001
**Two-sided Pr > |Z|**	<.0001

Sample Size = 600

Proportion Larvae-Pupae with SE by Group

The FREQ Procedure
GROUP=2=CNP

LARVAE_PUPAE_YES	Frequency	Percent	Cumulative Frequency	Cumulative Percent
Yes	68	9.71	68	9.71
No	632	90.29	700	100.00

Binomial Proportion	
LARVAE_PUPAE_YES = Yes	
Proportion	0.0971
ASE	0.0112
95% Lower Conf Limit	0.0752
95% Upper Conf Limit	0.1191
Exact Conf Limits	
95% Lower Conf Limit	0.0762
95% Upper Conf Limit	0.1215

Test of H0: Proportion = 0.5	
ASE under H0	0.0189
Z	-21.3172
One-sided Pr < Z	<.0001
Two-sided Pr > |Z|	<.0001

Sample Size = 700

Proportion Larvae-Pupae with SE by Group

The FREQ Procedure
GROUP=3=AgCNP

LARVAE_PUPAE_YES	Frequency	Percent	Cumulative Frequency	Cumulative Percent
Yes	98	16.33	98	16.33
No	502	83.67	600	100.00

Binomial Proportion

LARVAE_PUPAE_YES = Yes	
Proportion	0.1633
ASE	0.0151
95% Lower Conf Limit	0.1338
95% Upper Conf Limit	0.1929
Exact Conf Limits	
95% Lower Conf Limit	0.1346
95% Upper Conf Limit	0.1954

Test of H0: Proportion = 0.5	
ASE under H0	0.0204
Z	-16.4932
One-sided Pr < Z	<.0001
Two-sided Pr > |Z|	<.0001

Sample Size = 600

Proportion Larvae-Pupae with SE by Group

The FREQ Procedure
GROUP=4=CNP+AgNO3

LARVAE_PUPAE_YES	Frequency	Percent	Cumulative Frequency	Cumulative Percent
**Yes**	85	12.14	85	12.14
**No**	615	87.86	700	100.00

Binomial Proportion	
**LARVAE_PUPAE_YES = Yes**	
**Proportion**	0.1214
**ASE**	0.0123
**95% Lower Conf Limit**	0.0972
**95% Upper Conf Limit**	0.1456
**Exact Conf Limits**	
**95% Lower Conf Limit**	0.0982
**95% Upper Conf Limit**	0.1479

Test of H0: Proportion = 0.5	
**ASE under H0**	0.0189
**Z**	-20.0321
**One-sided Pr < Z**	<.0001
**Two-sided Pr > |Z|**	<.0001

Sample Size = 700

Proportion Larvae-Pupae with SE by Group

The FREQ Procedure
GROUP=5=AgNO3

LARVAE_PUPAE_YES	Frequency	Percent	Cumulative Frequency	Cumulative Percent
Yes	63	10.50	63	10.50
No	537	89.50	600	100.00

Binomial Proportion

LARVAE_PUPAE_YES = Yes	
Proportion	0.1050
ASE	0.0125
95% Lower Conf Limit	0.0805
95% Upper Conf Limit	0.1295
Exact Conf Limits	
95% Lower Conf Limit	0.0816
95% Upper Conf Limit	0.1323

Test of H0: Proportion = 0.5	
ASE under H0	0.0204
Z	-19.3510
One-sided Pr < Z	<.0001
Two-sided Pr > |Z|	<.0001

Sample Size = 600

Tukey Style Multiple Comparisons of Proportions

Compare	Diff	SE	q	q(.05)	Conclude
3 vs 2	5.67	1.13	5.01	3.585	R
3 vs 5	4.91	1.17	4.2	3.585	R
3 vs 1	4	1.17	3.42	3.585	A
3 vs 4	3.44	1.13	3.04	3.585	A
4 vs 2	2.23	1.08	2.06	3.585	A
4 vs 5	1.47	1.13	1.3	3.585	A
4 vs 1	0.56	1.13	0.5	3.585	A
1 vs 2	1.66	1.13	1.47	3.585	A
1 vs 5	0.91	1.17	0.78	3.585	A
5 vs 2	0.75	1.13	0.67	3.585	A

Tukey Style Multiple Comparisons of Proportions

Compare	Diff	SE	q	q(.01)	Conclude
3 vs 2	5.67	1.13	5.01	4.603	R
3 vs 5	4.91	1.17	4.2	4.603	A
3 vs 1	4	1.17	3.42	4.603	A
3 vs 4	3.44	1.13	3.04	4.603	A
4 vs 2	2.23	1.08	2.06	4.603	A
4 vs 5	1.47	1.13	1.3	4.603	A
4 vs 1	0.56	1.13	0.5	4.603	A
1 vs 2	1.66	1.13	1.47	4.603	A
1 vs 5	0.91	1.17	0.78	4.603	A
5 vs 2	0.75	1.13	0.67	4.603	A

Compare	Diff	SE	q	q(.001)	Conclude
3 vs 2	5.67	1.13	5.01	5.484	A
3 vs 5	4.91	1.17	4.2	5.484	A
3 vs 1	4	1.17	3.42	5.484	A
3 vs 4	3.44	1.13	3.04	5.484	A
4 vs 2	2.23	1.08	2.06	5.484	A
4 vs 5	1.47	1.13	1.3	5.484	A
4 vs 1	0.56	1.13	0.5	5.484	A
1 vs 2	1.66	1.13	1.47	5.484	A
1 vs 5	0.91	1.17	0.78	5.484	A
5 vs 2	0.75	1.13	0.67	5.484	A

Figure 10d-% Female

Proportion Female with SE by Group

The FREQ Procedure

**For FEMALES_YES * GROUP
all data are missing because
all levels of variable FEMALES_YES are
missing.**

Proportion Female with SE by Group

The FREQ Procedure

Frequency<br>Col Pct	Table of FEMALES_YES by GROUP						
	**FEMALES_YES**	**GROUP**					
		**1=Control**	**2=CNP**	**3=AgCNP**	**4=CNP+AgNO3**	**5=AgNO3**	**Total**
	**Yes**	245<br>53.49	259<br>50.00	190<br>48.22	234<br>47.56	213<br>45.91	1141
	**No**	213<br>46.51	259<br>50.00	204<br>51.78	258<br>52.44	251<br>54.09	1185
	**Total**	458	518	394	492	464	2326

Statistics for Table of FEMALES_YES by GROUP

Statistic	DF	Value	Prob
**Chi-Square**	4	6.1860	0.1857
**Likelihood Ratio Chi-Square**	4	6.1896	0.1854
**Mantel-Haenszel Chi-Square**	1	5.7432	0.0166
**Phi Coefficient**		0.0516	
**Contingency Coefficient**		0.0515	
**Cramer's V**		0.0516	

Sample Size = 2326

Proportion Female with SE by Group

The FREQ Procedure
GROUP=1=Control

FEMALES_YES	Frequency	Percent	Cumulative Frequency	Cumulative Percent
**Yes**	245	53.49	245	53.49
**No**	213	46.51	458	100.00

Binomial Proportion	
**FEMALES_YES = Yes**	
**Proportion**	0.5349
**ASE**	0.0233
**95% Lower Conf Limit**	0.4893
**95% Upper Conf Limit**	0.5806
**Exact Conf Limits**	
**95% Lower Conf Limit**	0.4881
**95% Upper Conf Limit**	0.5814

Test of H0: Proportion = 0.5	
**ASE under H0**	0.0234
**Z**	1.4953
**One-sided Pr > Z**	0.0674
**Two-sided Pr > |Z|**	0.1348

Sample Size = 458

Proportion Female with SE by Group

The FREQ Procedure
GROUP=2=CNP

FEMALES_YES	Frequency	Percent	Cumulative Frequency	Cumulative Percent
Yes	259	50.00	259	50.00
No	259	50.00	518	100.00

Binomial Proportion	
FEMALES_YES = Yes	
Proportion	0.5000
ASE	0.0220
95% Lower Conf Limit	0.4569
95% Upper Conf Limit	0.5431
Exact Conf Limits	
95% Lower Conf Limit	0.4561
95% Upper Conf Limit	0.5439

Test of H0: Proportion = 0.5	
ASE under H0	0.0220
Z	0.0000
One-sided Pr < Z	0.5000
Two-sided Pr > |Z|	1.0000

Sample Size = 518

Proportion Female with SE by Group

The FREQ Procedure
GROUP=3=AgCNP

FEMALES_YES	Frequency	Percent	Cumulative Frequency	Cumulative Percent
**Yes**	190	48.22	190	48.22
**No**	204	51.78	394	100.00

**Binomial Proportion**	
**FEMALES_YES = Yes**	
**Proportion**	0.4822
**ASE**	0.0252
**95% Lower Conf Limit**	0.4329
**95% Upper Conf Limit**	0.5316
**Exact Conf Limits**	
**95% Lower Conf Limit**	0.4319
**95% Upper Conf Limit**	0.5328

**Test of H0: Proportion = 0.5**	
**ASE under H0**	0.0252
**Z**	-0.7053
**One-sided Pr < Z**	0.2403
**Two-sided Pr > |Z|**	0.4806

Sample Size = 394

Proportion Female with SE by Group

The FREQ Procedure
GROUP=4=CNP+AgNO3

FEMALES_YES	Frequency	Percent	Cumulative Frequency	Cumulative Percent
Yes	234	47.56	234	47.56
No	258	52.44	492	100.00

Binomial Proportion	
FEMALES_YES = Yes	
Proportion	0.4756
ASE	0.0225
95% Lower Conf Limit	0.4315
95% Upper Conf Limit	0.5197
Exact Conf Limits	
95% Lower Conf Limit	0.4307
95% Upper Conf Limit	0.5208

Test of H0: Proportion = 0.5	
ASE under H0	0.0225
Z	-1.0820
One-sided Pr < Z	0.1396
Two-sided Pr > |Z|	0.2793

Sample Size = 492

Proportion Female with SE by Group

The FREQ Procedure
GROUP=5=AgNO3

FEMALES_YES	Frequency	Percent	Cumulative Frequency	Cumulative Percent
**Yes**	213	45.91	213	45.91
**No**	251	54.09	464	100.00

**Binomial Proportion**	
**FEMALES_YES = Yes**	
**Proportion**	0.4591
**ASE**	0.0231
**95% Lower Conf Limit**	0.4137
**95% Upper Conf Limit**	0.5044
**Exact Conf Limits**	
**95% Lower Conf Limit**	0.4130
**95% Upper Conf Limit**	0.5056

**Test of H0: Proportion = 0.5**	
**ASE under H0**	0.0232
**Z**	-1.7641
**One-sided Pr < Z**	0.0389
**Two-sided Pr > |Z|**	0.0777

Sample Size = 464
