# Supplementary material for: Exposure to nanoceria impacts larval survival, life history traits and fecundity of Aedes aegypti
Source: PLoS Negl Trop Dis. 2020 Sep 25;14(9):e0008654. doi: 10.1371/journal.pntd.0008654 (PMC7540862; doi:10.1371/journal.pntd.0008654)
Supplement: S7 File — (PDF) [file pntd.0008654.s011.pdf]

Figure 11a- Eggs per Female by Group

FIRST, we'll do the analysis using zeros in the data.

All groups failed to meet normality assumption via a number of tests.

Chi-Square Analysis for a 2x4 table

The NPAR1WAY Procedure

Wilcoxon Scores (Rank Sums) for Variable EGGS Classified by Variable GROUP

GROUP	N	Sum of Scores	Expected Under H0	Std Dev Under H0	Mean Score
**1=Blood**	83	19955.00	18094.0	1030.10798	240.421687
**2=CNP**	88	18448.50	19184.0	1053.12142	209.642045
**3=AgCNP**	88	15900.00	19184.0	1053.12142	180.681818
**4=AgNP**	86	19329.50	18748.0	1044.08128	224.761628
**5=AgNO3**	90	21197.00	19620.0	1061.94781	235.522222

Average scores were used for ties.

Kruskal-Wallis Test

Chi-Square	DF	Pr > ChiSq
12.7849	4	0.0124

GROUP=1=Blood

Analysis Variable : EGGS

N	Minimum	Maximum	Mean	Median	Std Dev	Std Error	Lower 95% CL for Mean	Upper 95% CL for Mean
83	0	135.0000000	84.0361446	84.0000000	25.7452083	2.8259037	78.4145218	89.6577673

GROUP=2=CNP

Analysis Variable : EGGS

N	Minimum	Maximum	Mean	Median	Std Dev	Std Error	Lower 95% CL for Mean	Upper 95% CL for Mean
88	0	132.0000000	77.5795455	78.0000000	27.3532539	2.9158667	71.7839447	83.3751462

GROUP=3=AgCNP

Analysis Variable : EGGS

N	Minimum	Maximum	Mean	Median	Std Dev	Std Error	Lower 95% CL for Mean	Upper 95% CL for Mean
88	0	121.0000000	69.5681818	74.5000000	29.4613698	3.1405926	63.3259140	75.8104496

GROUP=4=AgNP

Analysis Variable : EGGS

N	Minimum	Maximum	Mean	Median	Std Dev	Std Error	Lower 95% CL for Mean	Upper 95% CL for Mean
86	0	127.0000000	79.8488372	82.0000000	27.9909723	3.0183442	73.8475604	85.8501140

GROUP=5=AgNO3

Analysis Variable : EGGS

N	Minimum	Maximum	Mean	Median	Std Dev	Std Error	Lower 95% CL for Mean	Upper 95% CL for Mean
90	0	132.0000000	80.6444444	85.0000000	32.0867193	3.3822372	73.9240112	87.3648777

Group sample sizes not equal, or some ranks tied. Performed Dunn's test, alpha=0.05

Comparison group = GROUP_New

Compare	Diff	SE	q	q(0.05)	Conclude
1 vs 3	59.74	19.23	3.11	2.807	Reject
1 vs 2	30.78	19.23	1.6	2.807	Do not reject
1 vs 4	Do not reject (within non-sig. comparison)				
1 vs 5	Do not reject (within non-sig. comparison)				
5 vs 3	54.84	18.84	2.91	2.807	Reject
5 vs 2	Do not reject (within non-sig. comparison)				
5 vs 4	Do not reject (within non-sig. comparison)				
4 vs 3	44.08	19.06	2.31	2.807	Do not reject
4 vs 2	Do not reject (within non-sig. comparison)				
2 vs 3	Do not reject (within non-sig. comparison)				

Note: "Do not reject (within non-sig. comparison)" indicates that any comparison within the range of a non-significant comparison must also be non-significant.

Reference: Biostatistical Analysis, 4th Edition, J. Zar, 2010.

Removing zeros from the data results in normally distributed values for Eggs.

The MEANS Procedure

GROUP=1=Blood

Analysis Variable : EGGS

N	Minimum	Maximum	Mean	Median	Std Dev	Std Error	Lower 95% CL for Mean	Upper 95% CL for Mean
82	13.0000000	135.0000000	85.0609756	84.5000000	24.1402014	2.6658393	79.7567917	90.3651596

GROUP=2=CNP

Analysis Variable : EGGS

N	Minimum	Maximum	Mean	Median	Std Dev	Std Error	Lower 95% CL for Mean	Upper 95% CL for Mean
85	18.0000000	132.0000000	80.3176471	80.0000000	23.5028933	2.5492467	75.2481904	85.3871037

GROUP=3=AgCNP

Analysis Variable : EGGS

N	Minimum	Maximum	Mean	Median	Std Dev	Std Error	Lower 95% CL for Mean	Upper 95% CL for Mean
80	3.0000000	121.0000000	76.5250000	77.0000000	20.4146727	2.2824298	71.9819371	81.0680629

GROUP=4=AgNP

Analysis Variable : EGGS

N	Minimum	Maximum	Mean	Median	Std Dev	Std Error	Lower 95% CL for Mean	Upper 95% CL for Mean
82	27.0000000	127.0000000	83.7439024	84.5000000	22.1804112	2.4494167	78.8703316	88.6174733

GROUP=5=AgNO3

Analysis Variable : EGGS

N	Minimum	Maximum	Mean	Median	Std Dev	Std Error	Lower 95% CL for Mean	Upper 95% CL for Mean
82	44.0000000	132.0000000	88.5121951	88.5000000	20.6458939	2.2799576	83.9757947	93.0485955

Eggs Produced by Group

The GLM Procedure

Dependent Variable: EGGS

Source	DF	Sum of Squares	Mean Square	F Value	Pr > F
**Model**	4	6840.8557	1710.2139	3.46	0.0086
**Error**	406	200903.1784	494.8354		
**Corrected Total**	410	207744.0341			

The GLM Procedure
Least Squares Means

GROUP	EGGS LSMEAN	95% Confidence Limits	
**1=Blood**	85.060976	80.231854	89.890097
**2=CNP**	80.317647	75.574511	85.060783
**3=AgCNP**	76.525000	71.635887	81.414113
**4=AgNP**	83.743902	78.914781	88.573024
**5=AgNO3**	88.512195	83.683074	93.341317

1=Blood vs. 2=CNP, t-test

The TTEST Procedure

Variable: EGGS

GROUP	Method	N	Mean	Std Dev	Std Err	Minimum	Maximum
**1=Blood**		82	85.0610	24.1402	2.6658	13.0000	135.0
**2=CNP**		85	80.3176	23.5029	2.5492	18.0000	132.0
**Diff (1-2)**	**Pooled**		4.7433	23.8179	3.6868		
**Diff (1-2)**	**Satterthwaite**		4.7433		3.6885		

GROUP	Method	Mean	95% CL Mean		Std Dev	95% CL Std Dev	
**1=Blood**		85.0610	79.7568	90.3652	24.1402	20.9270	28.5283
**2=CNP**		80.3176	75.2482	85.3871	23.5029	20.4233	27.6847
**Diff (1-2)**	**Pooled**	4.7433	-2.5360	12.0226	23.8179	21.5019	26.6975
**Diff (1-2)**	**Satterthwaite**	4.7433	-2.5397	12.0264			

Method	Variances	DF	t Value	Pr > |t|
**Pooled**	Equal	165	1.29	0.2000
**Satterthwaite**	Unequal	164.35	1.29	0.2003

Equality of Variances

Method	Num DF	Den DF	F Value	Pr > F
**Folded F**	81	84	1.05	0.8075

=Blood vs. 3=AgCNP, t-test

The TTEST Procedure

Variable: EGGS

GROUP	Method	N	Mean	Std Dev	Std Err	Minimum	Maximum
**1=Blood**		82	85.0610	24.1402	2.6658	13.0000	135.0
**3=AgCNP**		80	76.5250	20.4147	2.2824	3.0000	121.0
**Diff (1-2)**	**Pooled**		8.5360	22.3784	3.5167		
**Diff (1-2)**	**Satterthwaite**		8.5360		3.5094		

GROUP	Method	Mean	95% CL Mean		Std Dev	95% CL Std Dev	
**1=Blood**		85.0610	79.7568	90.3652	24.1402	20.9270	28.5283
**3=AgCNP**		76.5250	71.9819	81.0681	20.4147	17.6679	24.1806
**Diff (1-2)**	**Pooled**	8.5360	1.5909	15.4811	22.3784	20.1720	25.1310
**Diff (1-2)**	**Satterthwaite**	8.5360	1.6041	15.4678			

Method	Variances	DF	t Value	Pr > |t|
**Pooled**	Equal	160	2.43	0.0163
**Satterthwaite**	Unequal	156.86	2.43	0.0161

Equality of Variances

Method	Num DF	Den DF	F Value	Pr > F
**Folded F**	81	79	1.40	0.1365

1=Blood vs. 4=AgNP, t-test

The TTEST Procedure

Variable: EGGS

GROUP	Method	N	Mean	Std Dev	Std Err	Minimum	Maximum
**1=Blood**		82	85.0610	24.1402	2.6658	13.0000	135.0
**4=AgNP**		82	83.7439	22.1804	2.4494	27.0000	127.0
**Diff (1-2)**	**Pooled**		1.3171	23.1810	3.6203		
**Diff (1-2)**	**Satterthwaite**		1.3171		3.6203		

GROUP	Method	Mean	95% CL Mean		Std Dev	95% CL Std Dev	
**1=Blood**		85.0610	79.7568	90.3652	24.1402	20.9270	28.5283
**4=AgNP**		83.7439	78.8703	88.6175	22.1804	19.2281	26.2122
**Diff (1-2)**	**Pooled**	1.3171	-5.8319	8.4661	23.1810	20.9082	26.0126
**Diff (1-2)**	**Satterthwaite**	1.3171	-5.8323	8.4665			

Method	Variances	DF	t Value	Pr > |t|
**Pooled**	Equal	162	0.36	0.7165
**Satterthwaite**	Unequal	160.85	0.36	0.7165

Equality of Variances

Method	Num DF	Den DF	F Value	Pr > F
**Folded F**	81	81	1.18	0.4477

1=Blood vs. 5=AgNO3, t-test

The TTEST Procedure

Variable: EGGS

GROUP	Method	N	Mean	Std Dev	Std Err	Minimum	Maximum
**1=Blood**		82	85.0610	24.1402	2.6658	13.0000	135.0
**5=AgNO3**		82	88.5122	20.6459	2.2800	44.0000	132.0
**Diff (1-2)**	**Pooled**		-3.4512	22.4611	3.5078		
**Diff (1-2)**	**Satterthwaite**		-3.4512		3.5078		

GROUP	Method	Mean	95% CL Mean		Std Dev	95% CL Std Dev	
**1=Blood**		85.0610	79.7568	90.3652	24.1402	20.9270	28.5283
**5=AgNO3**		88.5122	83.9758	93.0486	20.6459	17.8978	24.3988
**Diff (1-2)**	**Pooled**	-3.4512	-10.3782	3.4758	22.4611	20.2589	25.2047
**Diff (1-2)**	**Satterthwaite**	-3.4512	-10.3795	3.4770			

Method	Variances	DF	t Value	Pr > |t|
**Pooled**	Equal	162	-0.98	0.3267
**Satterthwaite**	Unequal	158.19	-0.98	0.3267

Equality of Variances

Method	Num DF	Den DF	F Value	Pr > F
**Folded F**	81	81	1.37	0.1615
